# Integrative analysis of multi-omics data to detect the underlying molecular mechanisms for obesity in vivo in humans

**DOI:** 10.1186/s40246-022-00388-x

**Published:** 2022-05-14

**Authors:** Qiang Zhang, Xiang-He Meng, Chuan Qiu, Hui Shen, Qi Zhao, Lan-Juan Zhao, Qing Tian, Chang-Qing Sun, Hong-Wen Deng

**Affiliations:** 1 Department of Community Nursing, School of Nursing and Health, Zhengzhou University, High-Tech Development Zone of States, Zhengzhou, 450001 Henan People’s Republic of China; 2 Tulane Center for Biomedical Informatics and Genomics, School of Medicine, Tulane University, New Orleans, LA 70112 USA; 3 Center for System Biology, Data Sciences, and Reproductive Health, School of Basic Medical Science, Central South University, Changsha, 410013 Hunan People’s Republic of China; 4 Department of Preventive Medicine, College of Medicine, University of Tennessee Health Science Center, Memphis, TN 38163 USA; 5 Department of Social Medicine and Health Management, College of Public Health, Zhengzhou University, High-Tech Development Zone of States, Zhengzhou, 450001 Henan People’s Republic of China

**Keywords:** Multi-omics integration, Genomic, Epigenomic, Transcriptomic, Metabolomic

## Abstract

**Background:**

Obesity is a complex, multifactorial condition in which genetic play an important role. Most of the systematic studies currently focuses on individual omics aspect and provide insightful yet limited knowledge about the comprehensive and complex crosstalk between various omics levels.

**Subjects and methods:**

Therefore, we performed a most comprehensive trans-omics study with various omics data from 104 subjects, to identify interactions/networks and particularly causal regulatory relationships within and especially those between omic molecules with the purpose to discover molecular genetic mechanisms underlying obesity etiology in vivo in humans.

**Results:**

By applying differentially analysis, we identified 8 differentially expressed hub genes (DEHGs), 14 differentially methylated regions (DMRs) and 12 differentially accumulated metabolites (DAMs) for obesity individually. By integrating those multi-omics biomarkers using Mendelian Randomization (MR) and network MR analyses, we identified 18 causal pathways with mediation effect. For the 20 biomarkers involved in those 18 pairs, 17 biomarkers were implicated in the pathophysiology of obesity or related diseases.

**Conclusions:**

The integration of trans-omics and MR analyses may provide us a holistic understanding of the underlying functional mechanisms, molecular regulatory information flow and the interactive molecular systems among different omic molecules for obesity risk and other complex diseases/traits.

**Supplementary Information:**

The online version contains supplementary material available at 10.1186/s40246-022-00388-x.

## Introduction

Obesity is a chronic metabolic disorder mainly characterized by excessive body fat. Body mass index (BMI) is widely used in obesity research and clinical diagnosis to quantify an individual’s tissue mass. Epidemiological studies estimate that the elevated BMI level is a driving force for the similarly rapid increase of cardiovascular diseases, insulin resistance, type 2 diabetes (T2D), and certain types of cancer [1]. Heritability studies have demonstrated a substantial genetic contribution to obesity risk (h^2^ ~ 40–70%) [2]. Identification of the genetic determinants for BMI could lead to a better understanding of the biological basis of obesity.

Most systematic studies currently are focused on single to two omics measurements such as at DNA, RNA, metabolite levels. Although useful, little knowledge has been obtained about the cross-talks between molecules of various omics levels and the underlying biological networks that drive complex phenotypes. With the advance of emerging high-throughput sequencing technology such as whole genome sequencing (WGS), RNA sequencing (RNA-Seq), reduced-representation bisulfite sequencing (RRBS) and liquid chromatography–mass spectrometry (LC–MS), multi-omics data including genomics, transcriptomics, epigenomics and metabolomics are rapidly generated and accumulated [3]. As a result, more and more researchers are currently working on the integration of comprehensive multi-omics data to discovery new and meaningful biological knowledge [4, 5], but those studies focused on obesity are rare.

Peripheral blood monocytes (PBMs) or whole blood cells are emerging as a potent source of transcriptomic and epigenetic biomarkers of diabetes and obesity and their related metabolic alterations [6–8]. Therefore, we will use PBMs as an example cell type for illustration for this integrative multi-omics study for obesity.

In the current study, we intend to perform systematic genetics analysis which integrates genomic, transcriptomic (from PBM), epigenomic (from PBM) and metabolomic (serum) data of BMI—a major index for obesity, to identify potential molecular and genomic factors/mechanisms underlying the pathogenesis of obesity at different omics level and to re-construct functional module networks to discover the potential regulatory patterns for obesity. Additionally, we seek to identify the significant interactions/networks/causal regulatory relationships within and especially those between omics molecules, shedding lights into the in vivo functional mechanisms for obesity etiology in humans.

### Subjects and methods

The study was approved by Tulane University (New Orleans, USA) Institutional Review Board and all participants were required to sign informed consent documents before taking part in the study. A total of 104 premenopausal Caucasian females aged 25–40 years were derived from Louisiana Osteoporosis Study (LOS), an ongoing cohort recruitment since 2011 [9]. The inclusion and exclusion criteria were detailed in our previous studies [10, 11]. All the participants completed an interviewer-assisted comprehensive questionnaire to collect their baseline information including demographic characteristics (age, weight, and height) and life factors (smoking, drinking and exercise, etc.). Weight was measured in light indoor clothing using a calibrated balance beam scale, and height was measured using a calibrated stadiometer without shoes. BMI was calculated as weight (kg) divided by height squared (m^2^). For the ease of differential analysis, the subjects were categorized into normal weight group and overweight/obesity group according to the WHO criteria.

Peripheral blood mononuclear cells (PBMCs) were isolated from fresh blood from each subject using Lymphoprep™ (Axis-Shield, Oslo, Norway), PBMs were then isolated from PBMCs with Dynabeads® Untouched™ Human Monocytes kit (Life Technologies, CA, USA) using a previously established and routinely performed protocol [12, 13]. Then genomic DNA and total RNA were extracted from the freshly isolated PBMs with the AllPrep® DNA/RNA/ miRNA Universal Kit (Qiagen, CA, USA) and kept at -80℃ for further use. After the collection of whole blood sample, the blood was left undisturbed in room temperature for 15–30 min for coagulation. Then we centrifuged the blood at 1000–2000×*g* for 10 min to remove the clot, the resulting supernatant yielded the needed serum. Following the centrifugation, the serum was immediately transferred into a clean polypropylene tube and stored at − 80 ℃ or lower. To systematically illuminate the underlying functional mechanisms of obesity, WGS, RRBS (PBM), RNA-seq (PBM), and LC–MS (serum) were performed on the DNA, RNA, and metabolites, respectively.

### WGS, RNA-seq, DNA methylation and metabolomic analysis

The WGS, RNA-Seq and epigenome-wide DNA methylation profiling (identified by RRBS) were performed by Technology Center for Genomics & Bioinformatics (TCGB) at University of California, Los Angeles (UCLA). Libraries for WGS were prepared with KAPA DNA LTP library preparation kit (KAPA Biosystem) on Biomek FX Laboratory Automation Workstation (Beckman Coulter). Libraries for RNA-Seq were prepared with KAPA RNA Hyper kit with RiboErase (KAPA Biosystem, USA) according to manufacturer’s instructions. The detailed library construction procedures, library concentration and quality measurement, sequencing protocols and epigenomic analysis methods were described in our previous studies [11, 14, 15].

The LC–MS based metabolomics platform developed by Dr. Garrett’s lab in the Southeast Center for Integrated Metabolomics at University of Florida was used to perform the metabolomic analysis of the study. The detailed laboratory protocols and metabolomics analyses were described in our previous studies [11, 14, 16, 17]. The detailed quality control information for the three omics were also described in our previous study [11].

### Statistical analysis

For RNA-seq data, we first performed data filtering and normalization [18], then empirical Bayes method [19] was used to fit linear models to identify the differentially expressed genes (DEGs) between two groups. Genes with adjusted *P* value ≤ 0.01 and absolute values of log transformed fold changes (|logFC|) ≥ 5 were considered as significant genes. Finally, those significant DEGs were subject to the Multiscale Embedded Gene Co-expression Network Analysis (MEGENA) [20] to identify functional co-expressed gene modules and DE hub genes (DEHGs) associated with obesity. MEGENA were implemented in four steps: Fast Planar Filtered Network construction (FPFNC), Multi-scale Clustering analysis (MCA), Multiscale Hub Analysis (MHA) and Cluster-Trait Association Analysis (CTA). For detailed information about the steps, please refer to the method of original MEGENA paper. Functional enrichment analysis was performed on those significant DEGs to using GO enrichment analysis (http://geneontology.org/docs/go-enrichment-analysis/).

For the RRBS data, we adopted logistic regression to identify the differentially methylated regions/bases (DMRs) of multiple CpG sites between the two groups (normal weight group vs overweight/obesity group). Logistic *P*-values were adjusted to FDR-based *Q*-values using the Sliding Linear Model (SLIM) method [21]. Methylation regions with *Q*-value < 0.01 and percent methylation difference (PMD) more than 10% were considered as significant DMRs. Functional enrichment analysis was performed on the genes those DMRs annotated to using DAVID Bioinformatics Resources 6.8 (https://david.ncifcrf.gov/), which provides a comprehensive set of functional annotation tools for investigators to understand biological meaning behind large list of genes.

For the LC–MS data, we conducted both partial least squares regression-discriminant analysis (PLS-DA) [22] and Logistic regression analysis to detect the differentially accumulated metabolites (DAMs) between two groups. Metabolites with variable importance in projection (VIP) more than 1 and logistic *P* values less than 0.05 were considered as significant DAMs. Functional enrichment analysis was performed on the identified significant DAMs using Metabolites Biological Role (MBROLE) 2.0 (http://csbg.cnb.csic.es/mbrole2) [23], which has been widely used to perform metabolites functional annotation and metabolite–protein and drug–protein interactions.

### Quantitative trait loci (QTL) analysis to generate the datasets for mendelian randomization (MR) analysis

To identify genetic variants underlying the variation of various omics profiles, we performed QTL analysis by “Matrix eQTL” R package [24] to identify the expression quantitative loci (eQTL), methylation QTL (meQTL) and metabolomic QTL (metaQTL) for multi-omics data individually. By performing the association analysis between genotype data and RNA expression/DNA methylation/metabolite data individually, eQTL/meQTL/metaQTL datasets were generated for further MR analysis. To reduce the computational burden, we only included DEHGs, DMRs with *Q*-value < 0.01 and PMD larger than 15%, and the significant DAMs for QTL and further MR analysis. Given the moderate sample size (*n* = 104), we defined the QTLs that achieve *P* < 1E−5 as significant QTLs.

### MR analysis among multi-omics data

To better understand the crosstalk among the multi-omics data, we first performed Spearman correlation analysis among DEHGs, DMRs and DAMs, and heatmap was generated to represent and visualize their correlation patterns.

Then we applied the bi-directional MR approach to DEHGs and DMRs, DMRs and DAMs, DEHGs and DAMs to detect the potential casual pairs among multi-omics data. To detect the putative causal pairs between gene expression and DNA methylation, we analyzed each gene-methylation pair twice, defining the exposure as either gene expression or methylation. Specifically, we first selected eQTLs with *P* < 1E−5 as IVs, then the effect estimates of these eQTLs on DNA methylation were extract from the meQTL datasets. When target SNPs were not available in the methylation datasets, we used proxy SNPs that were in high LD (*r*^2^ > 0.8) with the SNPs of interest.

Standard inverse-variance weighted (IVW), simple median and weighted median [25] approaches were utilized to assess the effect estimates of gene expression on DNA methylation. We also applied MR-Egger regression [26] and mendelian randomization pleiotropy residual sum and outlier (MR-PRESSO) analysis [27] to evaluate the overall horizontal pleiotropy among all the IVs. MR analysis was then performed on DNA methylation to gene expression as in the bi-directional MR analyses. Similar analysis was repeatedly performed on gene expression and metabolites, DNA methylation and metabolites. To prioritize the sets of the results, any test pairs with at least two MR methods showed *P* < 0.01 was considered as significant potential causal signals, and 0.01 < *P* < 0.05 was considered as suggestive evidence for potential causal association.

### Network MR analysis

We then applied the network MR analysis [28] to investigate whether there was mediation effect in those causal pathways. Network MR assumes that the causal effect of the exposure (X) on outcome (Y) is partially mediated through mediator (M). Therefore, the total effect of exposure on outcome are composed of direct effect and indirect effect. Genetic association between genetic variables with exposure (IV_X_), mediator (IV_M_), and outcome (IV_Y_) could be derived from the linear regression performed previously. The significant difference (*P* < 0.05) between indirect effect and total effect suggests the existence of mediation effect.

## Results

**Fig. 1:**
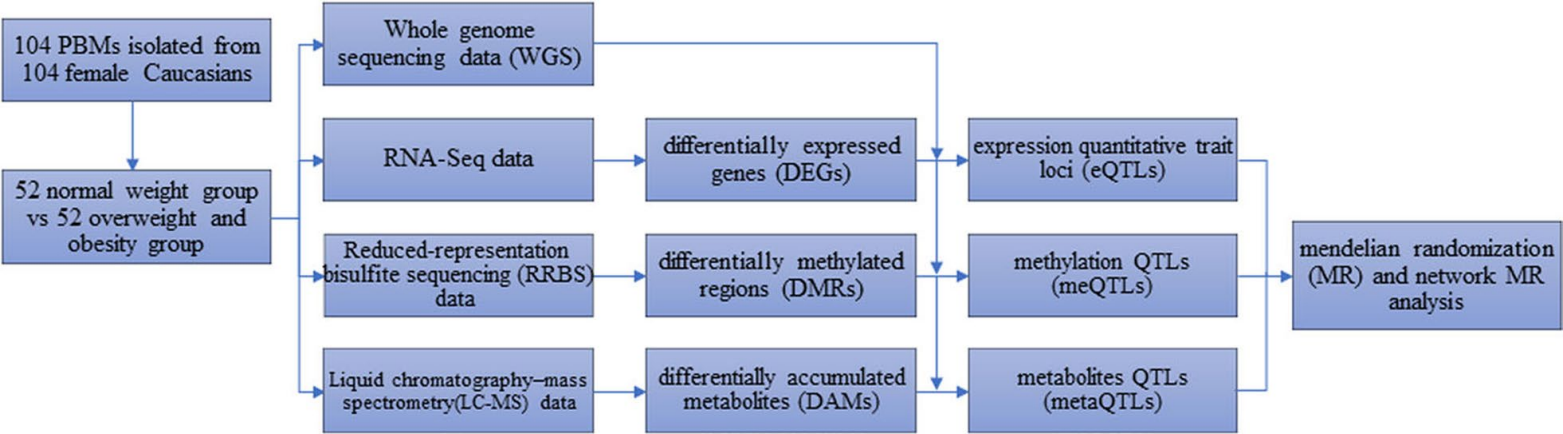
Workflow of the multi-omics analysis

**Fig. 2:**
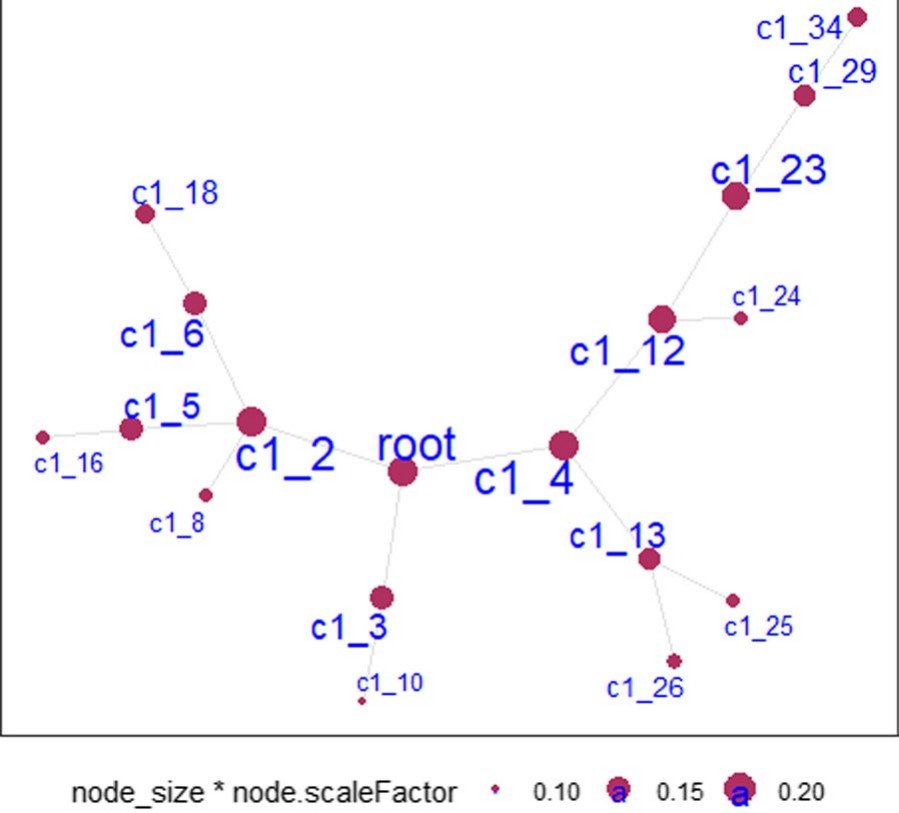
Modules hierarchy plot. Notes: Each node is a cluster identified by multiscale clustering in PFN. ‘c1_’ means the root node. Node_size: the size of the node. node.scaleFactor: scale number to adjust node sizes. Node size and label size are proportional to node degree. For detailed module clusters and complete list of genes in each module, please refer to Additional file 2: Table S2 and Additional file 1: Figs. S1–S4

Our omics workflow was demonstrated in Fig. 1. We observed significant difference in BMI and exercise between the two groups (*P* < 0.05), and the detailed information was shown in Table 1. Therefore, the following analysis for different omics all adjusted for “exercise”. For the RNA-Seq data, by fitting the gene expression data and BMI group into the linear regression model (adjusted for exercise), we identified 214 DEGs (adj*P* < 0.01, Additional file 2: Table S1). By using MEGENA, three scales groups (S1, S2, and S3) were identified that had similar interaction patterns and shared highly connected hubs across different scales. These genes were clustered into 17 gene modules (Table 2 and Additional file 2: Table S2) and 8 genes were identified as DEHGs. The module subnetwork figures (Additional file 1: Figs. S1–S4) were used to present the DEHGs of the specific module interconnected with obesity related genes. Modules hierarchy plot (Fig. 2) was generated to visualize the module hierarchical structure. These genes were enriched in obesity-related terms such as “Glycolysis”, “T cell activation”, “Blood coagulation” and “Integrin signaling pathway”. The results of GO term enrichment analysis were detailed in Additional file 2: Table S3.

**Table 1:** Characteristics of baseline information for all the individuals

Baseline information	Normal weight (*n* = 52)	Overweight/obesity (*n* = 52)	*P* value
Age (years)	30.98 ± 4.87	32.36 ± 5.42	0.396
BMI (kg/m^2^)	21.98 ± 1.68	32.85 ± 8.36	< 0.001
smoking (*n*/%)	17 (32.69)	22 (42.31)	0.198
Drinking (*n*/%)	47 (90.38)	42 (80.77)	0.195
Exercise (*n*/%)	44 (84.62)	35 (67.31)	0.029
Milk consumption (*n*/%)	37 (71.15)	39 (75.00)	0.549
Cheese consumption (*n*/%)	47 (90.38)	47 (90.38)	0.767

Quantitative data (age and BMI) was expressed as mean ± standard error(SE), student t test was performed to compare the difference between two groups

Enumeration data was expressed as number/percentage, *χ*^2^ test was performed to compare the difference between two groups

**Table 2:** Gene modules identified by MEGENA

ID	Module size	Module parent	Module hub	Module scale	Module *p* value
c1_2	95	c1_1	()	S3	< 1e−5
c1_3	29	c1_1	UGGT1 (13)	NA	< 1e−5
c1_4	90	c1_1	MPEG1 (21), IQGAP1 (19)	S3	< 1e−5
c1_5	30	c1_2	LUZP6*(13), ANO6 (12)	S3	< 1e−5
c1_6	35	c1_2	PTGS1 (15)	S3	< 1e−5
c1_8	12	c1_2	()	S3	< 1e−5
c1_10	10	c1_3	()	NA	< 1e−5
c1_12	64	c1_4	MPEG1 (21), IQGAP1 (19)	S2	< 1e−5
c1_13	26	c1_4	()	S2	0.04
c1_16	12	c1_5	ANO6(9)	S2	< 1e−5
c1_18	18	c1_6	PTGS1 (11), CLU (9)	NA	< 1e−5
c1_23	52	c1_12	MPEG1 (21), IQGAP1 (17)	NA	< 1e−5
c1_24	12	c1_12	()	S1	< 1e−5
c1_25	12	c1_13	PLCB2*(9)	S1	< 1e−5
c1_26	14	c1_13	()	S1	< 1e−5
c1_29	25	c1_23	MPEG1 (18)	NA	< 1e−5
c1_34	21	c1_29	MPEG1 (18)	S1	< 1e−5

Module size means the number of genes in each module, module parent means the beginning from connected components of the initial networks, module hub means the hub gene in this module, module scale demonstrates that three scales groups (S1, S2 and S3) were identified that had similar interaction patterns and shared highly connected hubs across different scales. The numbers inside the parenthesis in column 'module hub' mean the number of genes directed connected to the hub gene

By using the threshold of *Q*-value < 0.01 and PMD larger than 10%, we identified 95 DMRs (Additional file 2: Table S4), 35 of them were hyper-methylated regions/bases (Additional file 2: Table S5), and 60 were hypo-methylated regions/bases (Additional file 2: Table S6) when compared with the control group. Then those 95 DMRs were annotated to 67 nearest genes according to the distance to transcriptome start site (TSS) (Additional file 2: Table S7). After using a more stringent threshold of *Q*-value < 0.01 and PMD larger than 15%, there were 14 DMRs remained (Table 3), and they were annotated to 12 nearest genes. These genes were enriched in obesity-related terms such as “Cytoplasm”, and “transcription, DNA-templated”. The results of were detailed in Additional file 2: Table S8.

**Table 3:** DMRs with *Q*-value < 0.01 and PMD larger than 15%

Chr	Start	End	Strand	*P* value	*Q* value	Meth diff
17	75,284,971	75,284,971	+	1.76E−126	5.21E−123	18.64026
6	163,743,051	163,743,051	−	2.32E−55	1.54E−52	17.65037
11	129,594,021	129,594,021	−	1.30E−103	2.50E−100	17.05906
19	54,545,186	54,545,186	+	8.37E−79	9.76E−76	17.03821
9	137,131,610	137,131,610	+	2.93E−138	1.04E−134	15.9169
6	110,721,154	110,721,154	−	4.40E−55	2.84E−52	15.36733
6	110,721,178	110,721,178	−	1.65E−48	7.75E−46	15.18883
6	11,072,119	110,721,139	−	5.45E−55	3.46E−52	15.10461
8	28,227,281	28,227,281	−	2.27E−44	8.99E−42	− 15.2398
20	47,132,784	47,132,784	−	< 0	< 0	− 15.9148
X	86,937,439	86,937,439	−	8.41E−104	1.66E−100	− 16.45169
12	12,226,467	122,264,647	+	< 0	< 0	− 20.04055
5	355,049	355,049	−	1.31E−185	7.78E−182	− 21.24946
X	6,608,536	6,608,536	+	8.05E−205	7.16E−201	− 21.88445

By performing PLS-DA and logistic regression analysis, we identified 12 DAMs for obesity (Table 4). These metabolites were enriched in obesity-related terms such as “Amino sugar and nucleotide sugar metabolism”, “Amino Sugar Metabolism”, “Insulin signaling pathway” and “Type 2 diabetes mellitus”. The results of MBROLE term enrichment analysis were detailed in Additional file 2: Table S9.

**Table 4:** Differentially expressed metabolites between two groups

Metabolites	Estimate	SE	*P* value	VIP value
Glucosamine/Mannosamine	0.93	0.33	0.00	1.07
Ursodeoxycholic Acid (UDCA)	0.61	0.21	0.00	1.82
3-(2-hydroxyphenyl) propanoate	0.58	0.22	0.01	1.67
Plasmenyl-LysoPE (P-18:1)	− 0.63	0.25	0.01	1.56
N-methyl-D-aspartic acid*	− 0.55	0.22	0.01	1.43
Sphingosine (d18:1)	0.53	0.21	0.01	2.12
2-deoxy-D-galactose (fructose/glucose)	− 0.65	0.26	0.01	1.35
Phenylalanyl-Threonine/Threoninyl-Phenylalanine	0.52	0.22	0.02	1.37
Indole-3-acetate*	− 0.51	0.22	0.02	1.07
Isobutyrylcarnitine	− 0.51	0.24	0.03	1.07
N-Acetylneuraminate	0.55	0.26	0.04	1.35
Aspartate	− 0.54	0.26	0.04	1.17

^*^Denotes the novel metabolites discovered in the current study

### Bi-directional mendelian randomization analysis among multi-omics data

**Fig. 3:**
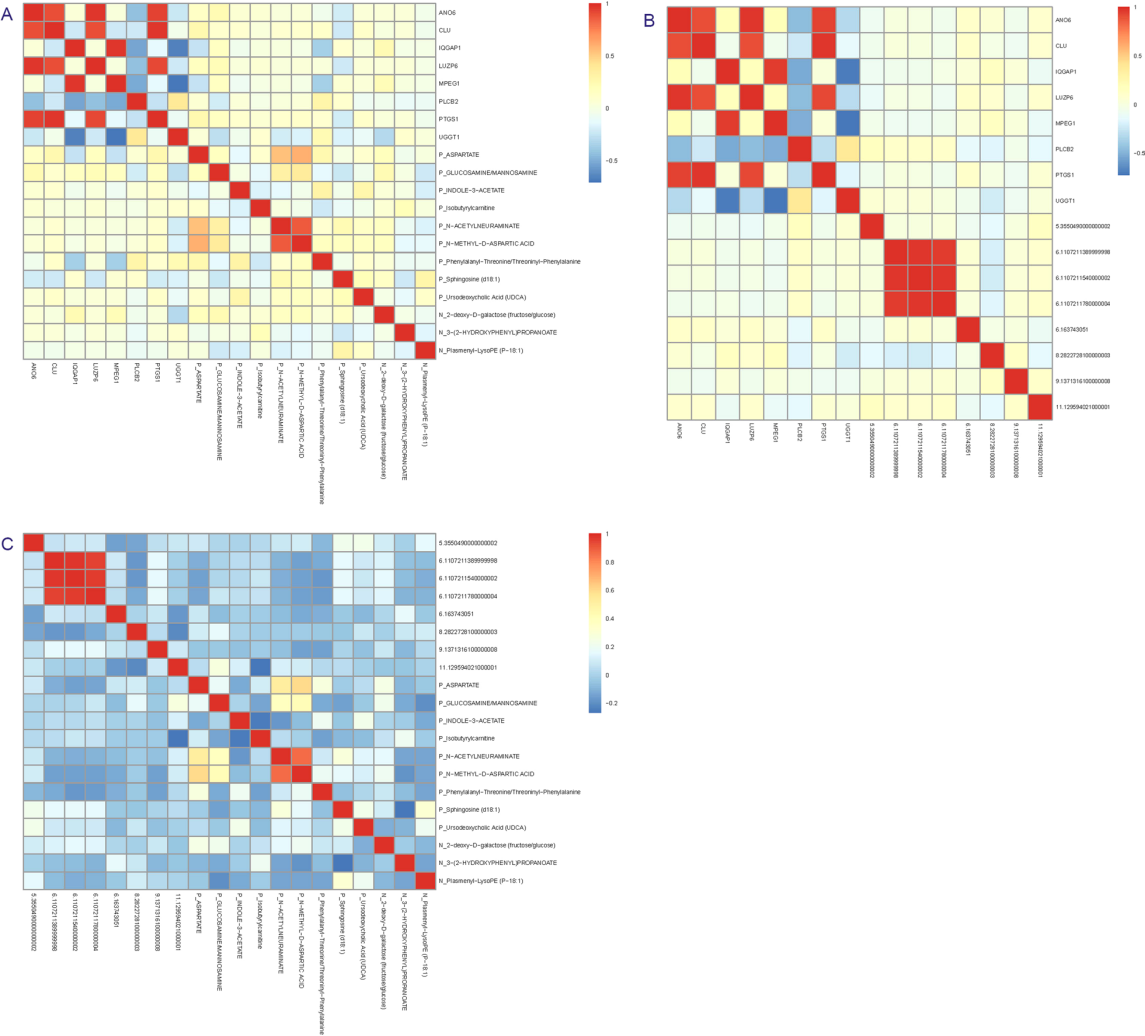
Correlation pattern between **A** DEHGs and DAMs, **B** DEHGs and DMRs, and **C** DMRs and DAMs

Spearman correlation analysis demonstrates significant correlation patterns among gene expression, DNA methylation and metabolites (Fig. 3). By performing association analysis between gene expression, DNA methylation, metabolites and genotype data separately, we successfully identified 3560 eQTLs (*P* < 1E−5) for the DEHGs, 734 meQTLs (*P* < 1E−5) for the DMRs, and 9055 metaQTLs (*P* < 1E−5) for DAMs.

We identified 7 of the 112 (8 × 14) DEHG-DMR site pairs with predicted causal direction from the bi-directional MR analyses. Within our 7 predicted causal pairs, five predicted DEHG to causally influence DMR (Additional file 2: Table S10 and Additional file 1: Fig. S5) and two predict DMR to causally influence DEHG. As an example, we highlighted one predicted gene → methylation causal pair ANO6 → 6.110721178. Elevated ANO6 expression was significantly associated with increasing 6.110721178 methylation [*β* = 2.324, 95% CI (0.691, 3.957), *P*_IVW_ = 0.005], while 6.110721178 methylation was not associated with ANO6 expression (Additional file 2: Table S10).

We found 40 of the 168 (12 × 14) DMR-DAM site pars with predicted causal direction. Within those 40 observed causal pairs, 31 predicted DAMs were significantly associated with DMRs, 8 suggested DAMs have suggestively causal association with DMRs, and one predicted DMR was causally associated with DAM (Additional file 2: Table S11 and Additional file 1: Figs. S6–S11). We exemplified one predicted causal pair from metabolite to methylation here: isobutyrylcarnitine → 6.110721178. Increased isobutyrylcarnitine was significantly causally associated with increasing 6.110721178 methylation [*β* = 3.25E−06, 95% CI (1.464E−06, 5.032E−06), *P*_IVW_ = 0.0003], while 6.110721178 methylation did not show any reverse causal association with isobutyrylcarnitine (Additional file 2: Table S11).

We detected 42 of the 96 (8 × 12) DEHG-DAM site pairs with precited causal direction. Within our 42 predicted causal relationships, 12 predicted DEHG to causally influence DAM and 30 predicted DAM to causally influence DEHG (Additional file 2: Table S12 and Additional file 1: Figs. S12–S17). We demonstrated one of the gene and metabolite causal pairs as an illustration of the causal relationships. Increased ANO6 expression was significantly associated decreasing fructose [*β* =  − 13.732, 95% CI (− 23.070, − 4.394), *P*_IVW_ = 0.004], though there was no causal association from fructose to ANO6 expression (Additional file 2: Table S12).

### Network MR analysis result

Our network MR identified 18 causal pairs with mediation effect (Table 5), including eight causal pathways DAMs → DEHGs → DMRs, four DAMs → DMRs → DEHGs causal pathways and six causal pairs pathways DEHGs → DAMs → DMRs. There were 20 biomarkers involved in those pathways. We highlighted one of those causal pathways: isobutyrylcarnitine_ANO6_6.110721178. We first assessed the causal association between metabolite level of isobutyrylcarnitine and expression level of gene ANO6, it turned out higher isobutyrylcarnitine level was causally associated with the decreasing expression of gene ANO6 (*P* = 0.003), however the reverse did not suggest any causal association (*P* = 0.447). Then we tested the association status between expression level of gene ANO6 and methylation level of the region 6.110721178, and the results showed that elevated ANO6 expression level was causally associated with increasing methylation of region 6.110721178 (*P* = 0.005), but the reverse MR showed no association. Additionally, the mediation analysis showed significant difference between indirect and total effect (*P* = 0.012), which suggests that the causal association between isobutyrylcarnitine and 6.110721178 was partially mediated by gene ANO6.

**Table 5:** Network MR results for the causal pathways

Causal pairs among different omics			*β*	Se	*P* value
Metabolomic → Transcriptomic → Epigenomic					
Isobutyrylcarnitine	ANO6	6.110721178	− 3.486E−05	1.555E−05	0.012
Plasmenyl-LysoPE	ANO6	6.110721178	− 0.007	0.003	0.013
3-(2-Hydroxyphenyl) Propanoate	ANO6	6.110721178	− 3.811E−05	2.146E−05	0.038
Ursodeoxycholic Acid (UDCA)	ANO6	6.110721178	− 7E−4	3E−04	0.013
Isobutyrylcarnitine	CLU	9.13713161	− 3.04E−07	1.730E−07	0.040
Plasmenyl-LysoPE	CLU	9.13713161	− 2E−05	1E−5	0.023
Indole-3acetate	MPEG1	6.163743051	− 9.1E−06	4.360E−06	0.018
3-(2-Hydroxyphenyl) Propanoate	MPEG1	6.163743051	− 6.5E−07	3.155E−07	0.020
Metabolomic → Epigenomic → Transcriptomic					
UDCA	6.163743051	ANO6	− 2.185E−4	9.377E−05	0.010
Glucosamine	6.163743051	ANO6	− 2.951E−05	1.650E−05	0.037
Glucosamine	6.110721139	PTGS1	− 6.491E−05	3.219E−05	0.022
3-(2-Hydroxyphenyl) Propanoate	6.110721139	PTGS1	− 1.774E−05	9.524E−06	0.031
Transcriptomic → Metabolomic → Epigenomic					
CLU	Aspartate	9.13713161	8.439E−4	4.23E−4	0.023
CLU	N-Acetylneuraminate	9.13713161	− 3.87E−4	2.0E−4	0.031
MPEG1	Phenylalanyl-Threonine	6.163743051	− 6.41E−4	3.61E−4	0.038
UGGT1	N-methyl-D-asparticacid	6.110721154	− 2.29E−4	1.33E−4	0.042
UGGT1	UDCA	6.110721178	− 1.60E−4	6.914E−05	0.010
UGGT1	UDCA	6.110721154	− 1.00E−4	4.77E−05	0.018

## Discussion

In the current study, we first identified significant DEGs, DMRs, and DAMs for obesity in single omics individually. Then, by integration of the multi-omics data (DEHGs, DMRs and DAMs) for obesity using MR analysis, we observed significant correlation among gene expression, DNA methylation and metabolites and identified several putative causal pathways among the various omics molecules. Finally, the application of network MR enabled us to detect 18 causal pathways with mediation effect among different omics.

### Implications of the identified DEHGs on obesity

For the identified DEHGs for obesity, there were six previously reported genes (*UGGT1*, *ANO6*, *MPEG1*, *PTGS1*, *CLU* and *IQGAP1*) and two novel genes (*LUZP6* and *PLCB2*) for obesity. Genes *UGGT1* and *ANO6* were previously reported to be associated with BMI in gluteal subcutaneous adipose tissue (GSAT) [29], *UGGT1* was also reported as hip-associated gene in GSAT [29]. Studies in animal model showed significant difference in gene expression of *MPEG1* between normal and obese mice [30]. Expression of gene *PTGS1* was reported up-regulated in the intestinal mucosa of the obese rats compared to lean rats [31], and study reported that *PTGS1* expression showed a significant positive correlation with BMI [32] in human subcutaneous tissue. Microarray analysis in female subcutaneous adipocytes found that *CLU* gene expression was upregulated in obese versus lean patients [33], and serum levels of gene *CLU* was elevated during T2D and coronary heart disease [34]. Studies reported that knockdown of *IQGAP1* significantly reduced the ability of glucose to stimulate insulin secretion from β-cell [35]. The rest two genes *LUZP6* and *PLCB2* were not implicated earlier in obesity or related diseases. However, an extracellular transcriptome study in saliva demonstrated that four extracellular RNA biomarkers including *LUZP6* had the potential to distinguish high and low insulin resistance participants [36]. Gene *PLCB2* was shown to exhibit diagnostic value for hepatocellular carcinoma [37], *PLCB2* also have an important role in the progression of Alzheimer’s disease and enriched in another neurodegenerative disorder Huntington’s disease [38]. Furthermore, studies with established evidence have reported the associations between cognitive dysfunction, insulin resistance, hepatocellular carcinoma and obesity [39, 40].

### Implications of the identified DMRs on obesity

For the identified DMRs, we focused on the 12 nearest genes they were annotated to. For those 12 genes, six of them (*DDO*, *SEPT9*, *TMEM45B*, *RXRα*, *ZNF395* and *AHRR*) were previously reported to be implicated in the obesity or related diseases and the rest six were novel (*PACRG*, *LINC00494*, *KLHL4*, *DTX1*, *VCX3A* and *VSTM1*). Specifically, according to the genotype–phenotype association of dbGap in Harmonize dataset (http://amp.pharm.mssm.edu/Harmonizome/), *DDO* was one of the genes that were associated with obesity. A GWAS in a cohort of 1,895 females reported that the variation in gene *SEPT9* was correlated with BMI [41]. Gene *TMEM45B* was proved differentially expressed in white adipose tissue between autism mouse model and wild type mouse model [42]. Also, research suggested that the methylation of gene *RXRα* was a diagnose biomarker for childhood obesity [43]. While the rest genes were not implicated earlier in the obesity or related diseases, previous research suggested their roles in other complex diseases. Genes *PACRG* and *VSTM1* were reported to be involved in the Parkinson [44] and rheumatoid arthritis [45], respectively. Studies already showed that overweight and obesity might be potential risk factors for Parkinson disease [46] and rheumatoid arthritis [47]. SNPs located in intron of *DTX1* were implicated in the process of immune response to HBV vaccination. Studies demonstrated *LINC00494* (long intergenic non-protein coding RNA 494) was enriched in prognosis-related long non-coding RNAs (lncRNAs) module that were involved in chemokine signaling pathway, acute myeloid leukemia and pathways in cancer [48]. SNP variants in gene *KLHL4* were associated with bone density [49] and HDL cholesterol [50]. *VCX3A* was reported to be associated with an abnormal neurocognitive phenotype, which plays a role in neurogenesis regulation [51]. Meanwhile, another gene *PNPLA4* in the same region was related to human obesity [52]. Although those genes were not directly implicated in obesity, previous studies showed their associations with other complex diseases that may be related to obesity risk [39, 53].

### Implications of the identified DAMs on obesity

As for the identified metabolites for obesity, previous studies reported ten of them (Table 5) were related to obesity, two were novel (Indole-3-acetate and N-methyl-D-aspartic acid (NMDA)). Oral treatment by glucosamine (GLC) in high-fat diet-induced obese rats demonstrated GLC’s effect in preventing body weight gains [54]. Studies of the pathways involved in the obesity and metabolic disorders have showed that ursodeoxycholic acid (UDCA) is used for the treatment of diseases related to liver disorders, especially cholestasis, obesity and lipemic frames [55], and studies in mouse model of diet-induced obesity also illustrated the effective of UDCA in the treatment of obesity by alleviating metabolic dysfunction [56, 57]. Previous studies involving LC–MS revealed that the sphingosine level in the adipose tissue was increased in obese mice compared to lean mice, furthermore, the plasma level of sphingosine was also indicated elevated in obese mice [58]. Established evidence reported that high consumption of beverage rich in fructose was directly associated with the obesity development and its complications [59, 60]. A study performed in Turkish population illustrated that the obesity prevalence in children with phenylketonuria and hyperphenylalaninaemia who received phenylalanine-restricted diet treatment was higher than that in the normal population [61]. Metabolomic profiling in both obese adults and children reported elevated isobutyrylcarnitine level in plasma in obese than that in lean subjects [62]. Metabolite aspartate was also reported as BMI-associated metabolite [63]. N-acetylneuraminate belongs to sialic acids (SAs) and takes up the highest content of them, SAs were widely common in human tissues and fluids, and research showed that increased level of SA was positively associated with coronary artery disease (CAD) [64]. While for metabolite 3-(2-hydroxyphenyl)propanoate, microbiome study in obese children and adults showed that propanoate was one of the metabolites that were associated with obese individuals [65], meanwhile, randomized control study of human diet revealed that propanoate level in plasma decreased with the decrease of the weight and it could be used as an independent predictor for insulin sensitivity [66]. As for plasmenyl-LysoPE, plasmenyl was highly accumulated in nerve, immune and cardiovascular systems, study reported it has the potential to protect the cells from reactive oxygen damage [67], and metabolomic profiling in obese males reported LysoPE was one of the DAMs [68]. To our acknowledge, this study for the first time reported the association of indole-3-acetate and NMDA with obesity. Metabolomics analysis demonstrated that indole-3-acetate was reported associated with carotid intima-media thickness, a validated surrogate marker of atherosclerotic vascular disease [69]. Furthermore, indole-3-acetate was reported could attenuate indicators of inflammation in macrophages and cytokine-mediated lipogenesis in hepatocytes [70]. NMDA receptors are responsible for the majority of excitatory synaptic transmission in the central nervous system, which have been implicated as mediators of neuronal damage caused by excess glutamate in multiple neurologic disorders, including stroke, epilepsy, trauma, and neurodegenerative disorders [71].

### Potential causal regulatory relationship between significant omics profiles

In this analysis, by innovatively using the bi-directional MR principle, we identified significant causal pairs between DEHGs and DMRs, DMRs and DAMs, DEHGs and DAMs. For these gene expression-methylation causal pairs, five of them have evidence of gene driving methylation and the rest two the reverse, which demonstrates that we cannot simply assume methylation always drives changes in gene expression in a model, this findings may represent true causal relationships of gene expression on methylation or methylation on gene expression, but are in themselves not proof. Our findings demonstrated the complex relationship among gene expression, methylation and metabolite, highlighting that the different omics data for complex disease do not simply model one driving another. Therefore, it would make more sense to identify the causal pathways among different omics rather than simply focus on the global relationship of different omics, which enable us to integrate data from different omics levels to reveal their interrelation and combined potential functional influence and pathways on the disease processes. The application of QTL analysis and the integration with MR approach enabled us to detect the specific causal pathways among different omics, which will provide us novel insights into etiology and potential mechanisms underlying complex diseases.

In the current study, our Spearman correlation analysis first demonstrated the significant correlation between biomarker pairs of different omics, then the bi-directional MR analysis further assessed their causal association. The results were partially validated by the previous evidence of their associations with obesity or related diseases. For the 20 biomarkers included in the mediation causal pairs, 17 of them were reported related to the development of obesity or related diseases, which were illustrated earlier. The rest three biomarkers, NMDA, indole-3-acetate and 6:163,743,051 (PACRG-AS1), although they were not implicated in the obesity, research showed their significance with other complicated obesity-related diseases. The results demonstrate the feasibility of the application of MR and network MR in multi-omics data integration, which deepen our understanding of the cross-talks between different omics of obesity and provide us novel insights into discovering the genetic flow information in the pathological of obesity.

### Strengths and limitations

Our study has several strengths. First, the application of MEGENA [20] in identifying the gene co-expression network not only helped us to prioritize meaningful and relevant co-expressed gene clusters for obesity and meanwhile reduces the computational burden for further QTL analysis. Secondly, MR analysis has been extensively applied to multiple integration analysis of multi-omics data such as gene expression and phenotypes, methylation and phenotypes, metabolite and phenotypes, gene expression and methylation. However, there was no application in detecting causal relationship among multi-omics data sets from gene expression, methylation, and metabolites simultaneously. Finally, and particularly, the application of network MR enables us to detect the mediation effect among the causal pathways, which provide us novel insights in unraveling the complex network underlying the mechanisms of obesity, and the biomarkers included in those pathways may serve as potential targets for future precision medicine. To our knowledge, this is the first reported study to integrate multi-omics data of obesity from same population using MR and network MR. We successful identified 18 mediation causal pathways among different omics, which demonstrates the feasibility of MR approach and its effectiveness in helping develop mechanistic insight into the etiology of obesity and other complex diseases.

There are several limitations in the current study. First, our sample size is relatively moderate so that our findings may be limited to those molecules and pathways with most significant effects. Second, current study subjects only included Caucasian females, which may not generalizable to male and other ethnicities. Last, further experimental validation is needed to confirm the biological functional of the identified potential causal pairs in this study.

## Conclusions

With the increasing availability of multi-omics or multilayer datasets for complex traits or diseases, the integration analysis of those datasets would be more helpful and powerful in solving the underlying mechanisms. By the application of MR in multi-omics datasets, we were able to untangle some of the crosstalks among various omics molecules and the underlying biological networks that drive the obesity and other complex phenotypes.

## Supplementary Information

**Additional file 1: Figures S1–S17** in.docx format are included in the supplementary information.

**Additional file 2: Tables S1–S12** in.docx format are included in the supplementary information.

## Data Availability

The datasets generated during and/or analyzed during the current study are available from the corresponding author upon reasonable request.

## Abbreviations

BMI: Body mass index
T2D: Type 2 diabetes
GWASs: Genome-wide association studies
WGS: Whole genome sequencing
RNA-Seq: RNA sequencing
RRBS: Reduced-representation bisulfite sequencing
LC–MS: Liquid chromatography–mass spectrometry
PBMs: Peripheral blood monocytes
LOS: Louisiana Osteoporosis Study
PBMCs: Peripheral blood mononuclear cells
DEGs: Differentially expressed genes
MEGENA: Multiscale Embedded Gene Co-expression Network Analysis
DMRs: Differentially methylated regions/bases
PMD: Percent methylation difference
PLS-DA: Partial least squares regression-discriminant analysis
DAMs: Differentially accumulated metabolites
VIP: Variable importance in projection
QTL: Quantitative trait loc
MR: Mendelian randomization
IVW: Inverse-variance weighted
MR-PRESSO: Mendelian randomization pleiotropy residual sum and outlier

## References

[1] Kopelman P (2007). Health risks associated with overweight and obesity. Obes Rev.

[2] Visscher PM, Brown MA, McCarthy MI, Yang J (2012). Five years of GWAS discovery. Am J Hum Genet.

[3] Hasin Y, Seldin M, Lusis A (2017). Multi-omics approaches to disease. Genome Biol.

[4] Ge S, Wang Y, Song M, Li X, Yu X, Wang H, Wang J, Zeng Q, Wang W (2018). Type 2 diabetes mellitus: integrative analysis of multiomics data for biomarker discovery. OMICS.

[5] Xu C, Zhang JG, Lin D, Zhang L, Shen H, Deng HW (2017). A systemic analysis of transcriptomic and epigenomic data to reveal regulation patterns for complex disease. G3 Bethesda.

[6] Keustermans GC, Kofink D, Eikendal A, de Jager W, Meerding J, Nuboer R, Waltenberger J, Kraaijeveld AO, Jukema JW, Sels JW, Garssen J, Prakken BJ, Asselbergs FW, Kalkhoven E, Hoefer IE, Pasterkamp G, Schipper HS (2017). Monocyte gene expression in childhood obesity is associated with obesity and complexity of atherosclerosis in adults. Sci Rep.

[7] Reynés B, Priego T, Cifre M, Oliver P, Palou A (2018). Peripheral blood cells, a transcriptomic tool in nutrigenomic and obesity studies: current state of the art. Compr Rev Food Sci Food Saf.

[8] Baccarelli A, Ghosh S (2012). Environmental exposures, epigenetics and cardiovascular disease. Curr Opin Clin Nutr Metab Care.

[9] Jeng C, Zhao LJ, Wu K, Zhou Y, Chen T, Deng HW (2018). Race and socioeconomic effect on sarcopenia and sarcopenic obesity in the Louisiana Osteoporosis Study (LOS). JCSM Clin Rep..

[10] He H, Liu Y, Tian Q, Papasian CJ, Hu T, Deng HW (2016). Relationship of sarcopenia and body composition with osteoporosis. Osteoporos Int.

[11] Qiu C, Yu F, Su K, Zhao Q, Zhang L, Xu C, Hu W, Wang Z, Zhao L, Tian Q, Wang Y, Deng H, Shen H (2020). Multi-omics data integration for identifying osteoporosis biomarkers and their biological interaction and causal mechanisms. Science.

[12] Lei SF, Wu S, Li LM, Deng FY, Xiao SM, Jiang C, Chen Y, Jiang H, Yang F, Tan LJ, Sun X, Zhu XZ, Liu MY, Liu YZ, Chen XD, Deng HW (2009). An in vivo genome wide gene expression study of circulating monocytes suggested GBP1, STAT1 and CXCL10 as novel risk genes for the differentiation of peak bone mass. Bone.

[13] Liu YZ, Dvornyk V, Lu Y, Shen H, Lappe JM, Recker RR, Deng HW (2005). A novel pathophysiological mechanism for osteoporosis suggested by an in vivo gene expression study of circulating monocytes. J Biol Chem.

[14] Yu F, Qiu C, Xu C, Tian Q, Zhao L-J, Wu L, Deng H-W, Shen H (2020). Mendelian randomization identifies CpG methylation sites with mediation effects for genetic influences on BMD in peripheral blood monocytes. Front Genet.

[15] Euan J, Rodger PA, Stockwell A, Chatter E.J.J.O. Biomedicine, biotechnology, Technical considerations for reduced representation bisulfite sequencing with multiplexed libraries. 2012. 10.1155/2012/741542 PMC3495292 23193365

[16] Zhao Q, Shen H, Su K-J, Zhang J-G, Tian Q, Zhao L-J, Qiu C, Zhang Q, Garrett TJ, Liu J, Deng H-W (2018). Metabolomic profiles associated with bone mineral density in US Caucasian women. Nutr Metab.

[17] Liu H, Garrett TJ, Su Z, Khoo C, Gu L (2017). UHPLC-Q-Orbitrap-HRMS-based global metabolomics reveal metabolome modifications in plasma of young women after cranberry juice consumption. J Nutr Biochem.

[18] Robinson MD, Oshlack A (2010). A scaling normalization method for differential expression analysis of RNA-seq data. Genome Biol.

[19] Smyth GK (2004). Linear models and empirical bayes methods for assessing differential expression in microarray experiments. Stat Appl Genet Mol Biol.

[20] Song WM, Zhang B (2015). Multiscale embedded gene co-expression network analysis. PLoS Comput Biol.

[21] Wang HQ, Tuominen LK, Tsai CJ (2011). SLIM: a sliding linear model for estimating the proportion of true null hypotheses in datasets with dependence structures. Bioinformatics.

[22] You YS, Lin CY, Liang HJ, Lee SH, Tsai KS, Chiou JM, Chen YC, Tsao CK, Chen JH (2014). Association between the metabolome and low bone mineral density in Taiwanese women determined by (1)H NMR spectroscopy. J Bone Miner Res.

[23] López-Ibáñez J, Pazos F, Chagoyen M (2016). MBROLE 2.0—functional enrichment of chemical compounds. Nucl Acids Res.

[24] Shabalin AA (2012). Matrix eQTL: ultra fast eQTL analysis via large matrix operations. Bioinformatics.

[25] Bowden J, Smith GD, Haycock PC, Burgess S (2016). Consistent estimation in Mendelian randomization with some invalid instruments using a weighted median estimator. Genet Epidemiol.

[26] Bowden J, Smith GD, Burgess S (2015). Mendelian randomization with invalid instruments: effect estimation and bias detection through Egger regression. Int J Epidemiol.

[27] Verbanck M, Chen C-Y, Neale B, Do R (2018). Detection of widespread horizontal pleiotropy in causal relationships inferred from Mendelian randomization between complex traits and diseases. Nat Genet.

[28] Burgess S, Daniel RM, Butterworth AS, Thompson SG (2015). Network Mendelian randomization: using genetic variants as instrumental variables to investigate mediation in causal pathways. Int J Epidemiol.

[29] Pinnick KE, Nicholson G, Manolopoulos KN, McQuaid SE, Valet P, Frayn KN, Denton N, Min JL, Zondervan KT, Fleckner J, McCarthy MI, Holmes CC, Karpe F (2014). Distinct developmental profile of lower-body adipose tissue defines resistance against obesity-associated metabolic complications. Diabetes.

[30] Xu P, Werner JU, Milerski S, Hamp CM, Kuzenko T, Jahnert M, Gottmann P, de Roy L, Warnecke D, Abaei A, Palmer A, Huber-Lang M, Durselen L, Rasche V, Schurmann A, Wabitsch M, Knippschild U (2018). Diet-induced obesity affects muscle regeneration after murine blunt muscle Trauma-A broad spectrum analysis. Front Physiol.

[31] Plaza-Diaz J, Robles-Sanchez C, Abadia-Molina F, Moron-Calvente V, Saez-Lara MJ, Ruiz-Bravo A, Jimenez-Valera M, Gil A, Gomez-Llorente C, Fontana L (2017). Adamdec1, Ednrb and Ptgs1/Cox1, inflammation genes upregulated in the intestinal mucosa of obese rats, are downregulated by three probiotic strains. Sci Rep.

[32] Quinkler M, Bujalska IJ, Tomlinson JW, Smith DM, Stewart PM (2006). Prostaglandin synthesis in adipose tissue from women with simple obesity reveals characteristic differences between omental and subcutaneous fat depots. Exp Clin Endocrinol Diabetes.

[33] Bradley D, Blaszczak A, Yin Z, Liu J, Joseph JJ, Wright V, Anandani K, Needleman B, Noria S, Renton D, Yearsley M, Wong STC, Hsueh WA (2019). Clusterin impairs hepatic insulin sensitivity and adipocyte clusterin associates with cardiometabolic risk. Diabetes Care.

[34] Trougakos IP, Poulakou M, Stathatos M, Chalikia A, Melidonis A, Gonos ES (2002). Serum levels of the senescence biomarker clusterin/apolipoprotein J increase significantly in diabetes type II and during development of coronary heart disease or at myocardial infarction. Exp Gerontol.

[35] Hedman AC, Smith JM, Sacks DB (2015). The biology of IQGAP proteins: beyond the cytoskeleton. EMBO Rep.

[36] Zhang Y, Sun J, Li F, Grogan TR, Vergara JL, Luan Q, Park M-S, Chia D, Elashoff D, Joshipura KJ, Wong DTW (2017). Salivary extracellular RNA biomarkers for insulin resistance detection in hispanics. Diabetes Res Clin Pract.

[37] Wang X, Huang K, Zeng X, Liu Z, Liao X, Yang C, Yu T, Han C, Zhu G, Qin W, Peng T (2019). Diagnostic and prognostic value of mRNA expression of phospholipase C β family genes in hepatitis B virus-associated hepatocellular carcinoma. Oncol Rep.

[38] Chang WS, Wang YH, Zhu XT, Wu CJ (2017). Genome-wide profiling of miRNA and mRNA expression in Alzheimer's disease. Med Sci Monit.

[39] Solas M, Milagro FI, Ramírez MJ, Martínez JA (2017). Inflammation and gut-brain axis link obesity to cognitive dysfunction: plausible pharmacological interventions. Curr Opin Pharmacol.

[40] Yaribeygi H, Maleki M, Sathyapalan T, Jamialahmadi T, Sahebkar A. Obesity and insulin resistance: a review of molecular interactions. Curr Mol Med. 2020. 10.2174/1566524020666200812221527 32787760

[41] Croteau-Chonka DC, Marvelle AF, Lange EM, Lee NR, Adair LS, Lange LA, Mohlke KL (2011). Genome-wide association study of anthropometric traits and evidence of interactions with age and study year in Filipino women. Obesity (Silver Spring, Md.).

[42] Liu X, Tamada K, Kishimoto R, Okubo H, Ise S, Ohta H, Ruf S, Nakatani J, Kohno N, Spitz F, Takumi T (2015). Transcriptome profiling of white adipose tissue in a mouse model for 15q duplication syndrome. Genomics Data.

[43] Godfrey KM, Sheppard A, Gluckman PD, Lillycrop KA, Burdge GC, McLean C, Rodford J, Slater-Jefferies JL, Garratt E, Crozier SR, Emerald BS, Gale CR, Inskip HM, Cooper C, Hanson MA (2011). Epigenetic gene promoter methylation at birth is associated with child's later adiposity. Diabetes.

[44] West AB, Lockhart PJ, O'Farell C, Farrer MJ (2003). Identification of a novel gene linked to parkin via a bi-directional promoter. J Mol Biol.

[45] Wang D, Li Y, Liu Y, He Y, Shi G (2016). Expression of VSTM1-v2 is increased in peripheral blood mononuclear cells from patients with rheumatoid arthritis and is correlated with disease activity. PLOS ONE.

[46] Martin-Jiménez CA, Gaitán-Vaca DM, Echeverria V, González J, Barreto GE (2017). Relationship between obesity, Alzheimer's disease, and Parkinson's disease: an astrocentric view. Mol Neurobiol.

[47] Dar L, Tiosano S, Watad A, Bragazzi NL, Zisman D, Comaneshter D, Cohen A, Amital H (2018). Are obesity and rheumatoid arthritis interrelated?. Int J Clin Pract.

[48] Pan JQ, Zhang YQ, Wang JH, Xu P, Wang W (2017). lncRNA co-expression network model for the prognostic analysis of acute myeloid leukemia. Int J Mol Med.

[49] Styrkarsdottir U, Halldorsson BV, Gretarsdottir S, Gudbjartsson DF, Walters GB, Ingvarsson T, Jonsdottir T, Saemundsdottir J, Snorradóttir S, Center JR, Nguyen TV, Alexandersen P, Gulcher JR, Eisman JA, Christiansen C, Sigurdsson G, Kong A, Thorsteinsdottir U, Stefansson K (2009). New sequence variants associated with bone mineral density. Nat Genet.

[50] Kathiresan S, Manning AK, Demissie S, D'Agostino RB, Surti A, Guiducci C, Gianniny L, Burtt NP, Melander O, Orho-Melander M, Arnett DK, Peloso GM, Ordovas JM, Cupples LA (2007). A genome-wide association study for blood lipid phenotypes in the Framingham Heart Study. BMC Med Genet.

[51] Sajan SA, Jhangiani SN, Muzny DM, Gibbs RA, Lupski JR, Glaze DG, Kaufmann WE, Skinner SA, Annese F, Friez MJ, Lane J, Percy AK, Neul JL (2017). Enrichment of mutations in chromatin regulators in people with Rett syndrome lacking mutations in MECP2. Genet Med.

[52] Preumont A, Rzem R, Vertommen D, Van Schaftingen E (2010). HDHD1, which is often deleted in X-linked ichthyosis, encodes a pseudouridine-5'-phosphatase. Biochem J.

[53] Savvidis C, Tournis S, Dede AD (2018). Obesity and bone metabolism. Hormones.

[54] Huang L, Chen J, Cao P, Pan H, Ding C, Xiao T, Zhang P, Guo J, Su Z (2015). Anti-obese effect of glucosamine and Chitosan oligosaccharide in high-fat diet-induced obese rats. Mar Drugs.

[55] Hofmann AF (1994). Pharmacology of ursodeoxycholic acid, an enterohepatic drug. Scand J Gastroenterol Suppl.

[56] Zhang Y, Zheng X, Huang F, Zhao A, Ge K, Zhao Q, Jia W. Ursodeoxycholic acid alters bile acid and fatty acid profiles in a mouse model of diet-induced obesity. 2019; 10(842). 10.3389/fphar.2019.00842 PMC6669341 31402868

[57] Mazzella G, Bazzoli F, Festi D, Ronchi M, Aldini R, Roda A, Grigolo B, Simoni P, Villanova N, Roda E (1991). Comparative evaluation of chenodeoxycholic and ursodeoxycholic acids in obese patients. Effects on biliary lipid metabolism during weight maintenance and weight reduction. Gastroenterology.

[58] Samad F, Hester KD, Yang G, Hannun YA, Bielawski J (2006). Altered adipose and plasma sphingolipid metabolism in obesity. A potential mechanism for cardiovascular and metabolic risk. Diabetes.

[59] Pereira RM, Botezelli JD, da Cruz-Rodrigues KC, Mekary RA, Cintra DE, Pauli JR, da Silva ASR, Ropelle ER, de Moura LP (2017). Fructose consumption in the development of obesity and the effects of different protocols of physical exercise on the hepatic metabolism. Nutrients.

[60] Rizkalla SW (2010). Health implications of fructose consumption: a review of recent data. Nutr Metab.

[61] Ozturk Y, Gencpinar P, Erdur B, Tokgöz Y, Isik I, Akin SB (2018). Overweight and obesity in children under phenylalanine restricted diet. Hong Kong J Paediatr.

[62] Baker PR, Boyle KE, Koves TR, Ilkayeva OR, Muoio DM, Houmard JA, Friedman JE (2015). Metabolomic analysis reveals altered skeletal muscle amino acid and fatty acid handling in obese humans. Obesity (Silver Spring, Md.).

[63] Cirulli ET, Guo L, Leon-Swisher C, Shah N, Huang L, Napier LA, Kirkness EF, Spector TD, Caskey CT, Thorens B, Venter JC, Telenti A (2019). Profound perturbation of the Metabolome in obesity is associated with health risk. Cell Metab.

[64] Rastam L, Lindberg G, Folsom AR, Burke GL, Nilsson-Ehle P, Lundblad A (1996). Association between serum sialic acid concentration and carotid atherosclerosis measured by B-mode ultrasound. The ARIC investigators. Atherosclerosis Risk in Communities Study. Int J Epidemiol.

[65] Del Chierico F, Abbatini F, Russo A, Quagliariello A, Reddel S, Capoccia D, Caccamo R, Ginanni Corradini S, Nobili V, De Peppo F, Dallapiccola B, Leonetti F, Silecchia G, Putignani L (2018). Gut microbiota markers in obese adolescent and adult patients: age-dependent differential patterns. Front Microbiol.

[66] Tirosh A, Calay ES, Tuncman G, Claiborn KC, Inouye KE, Eguchi K, Alcala M, Rathaus M, Hollander KS, Ron I, Livne R, Heianza Y, Qi L, Shai I, Garg R, Hotamisligil GS (2019). The short-chain fatty acid propionate increases glucagon and FABP4 production, impairing insulin action in mice and humans. Sci Transl Med.

[67] Moser AB, Steinberg SJ, Watkins PA, Moser HW, Ramaswamy K, Siegmund KD, Lee DR, Ely JJ, Ryder OA, Hacia JG (2011). Human and great ape red blood cells differ in plasmalogen levels and composition. Lipids Health Dis.

[68] Wang Y, Liu D, Li Y, Guo L, Cui Y, Zhang X, Li E (2016). Metabolomic analysis of serum from obese adults with hyperlipemia by UHPLC-Q-TOF MS/MS. Biomed Chromatogr.

[69] Boyd A, Boccara F, Meynard JL, Ichou F, Bastard JP, Fellahi S, Samri A, Sauce D, Haddour N, Autran B, Cohen A, Girard PM, Capeau J (2019). Serum tryptophan-derived quinolinate and indole-3-acetate are associated with carotid intima-media thickness and its evolution in HIV-infected treated adults. Open Forum Infect Dis.

[70] Krishnan S, Ding Y, Saedi N, Choi M, Sridharan GV, Sherr DH, Yarmush ML, Alaniz RC, Jayaraman A, Lee K (2018). Gut microbiota-derived tryptophan metabolites modulate inflammatory response in hepatocytes and macrophages. Cell Rep.

[71] Lapteva L, Nowak M, Yarboro CH, Takada K, Roebuck-Spencer T, Weickert T, Bleiberg J, Rosenstein D, Pao M, Patronas N, Steele S, Manzano M, van der Veen JW, Lipsky PE, Marenco S, Wesley R, Volpe B, Diamond B, Illei GG (2006). Anti-N-methyl-D-aspartate receptor antibodies, cognitive dysfunction, and depression in systemic lupus erythematosus. Arthritis Rheum.

