# Supplementary material for: Integrative analysis of multi-omics data to detect the underlying molecular mechanisms for obesity in vivo in humans
Source: Hum Genomics. 2022 May 14;16:15. doi: 10.1186/s40246-022-00388-x (PMC9107154; doi:10.1186/s40246-022-00388-x)
Supplement: Supplementary file 1 — Additional file 1: Figures S1–S17 in.docx format are included in the supplementary information. [file 40246_2022_388_MOESM1_ESM.docx]

**Supplementary Figure legends**

Figure S1 Co-expression modules (c1_3, c1_4 and c1_5) of identified hub genes with traits associated gene.

Figure S2 Co-expression modules (c1_6, c1_12 and c1_16) of identified hub genes with traits associated gene.

Figure S3 Co-expression modules (c1_18, c1_23 and c1_25) of identified hub genes with traits associated gene.

Figure S4 Co-expression modules (c1_29 and c1_34) of identified hub genes with traits associated gene.

Figure S5 MR analysis scatter plot: effect of DEGs/DMRs on DMRs/DEGs.

Figure S6 MR analysis scatter plot: effect of DAMs on DMR 6.110721178.

Figure S7 MR analysis scatter plot: effect of DAMs on DMR 9.13713161.

Figure S8 MR analysis scatter plot: effect of DAMs on DMR 6.163743051.

Figure S9 MR analysis scatter plot: effect of DAMs on DMR 6.110721154.

Figure S10 MR analysis scatter plot: effect of DAMs on DMR 6.110721139.

Figure S11 MR analysis scatter plot: effect of DAMs on DMR 11.129594021.

Figure S12 MR analysis scatter plot: effect of DEGs on DAMs.

Figure S13 MR analysis scatter plot: effect of 3-(2-Hydroxyphenyl) Propanoate on DEMs.

Figure S14 MR analysis scatter plot: effect of Glucosamine on DEMs.

Figure S15 MR analysis scatter plot: effect of Indole-3-Acetate, Isobutyrylcarnitine on DEMs.

Figure S16 MR analysis scatter plot: effect of N-Acetylneuraminate, Phenylalanyl-Threonine and UDCA on DEGs.

Figure S17 MR analysis scatter plot: effect of Plasmenyl-LysoPE, NMDA and Sphingosine on DEGs.

**Figure S1 Co-expression modules (c1_3, c1_4 and c1_5) of identified hub genes with traits associated gene.**


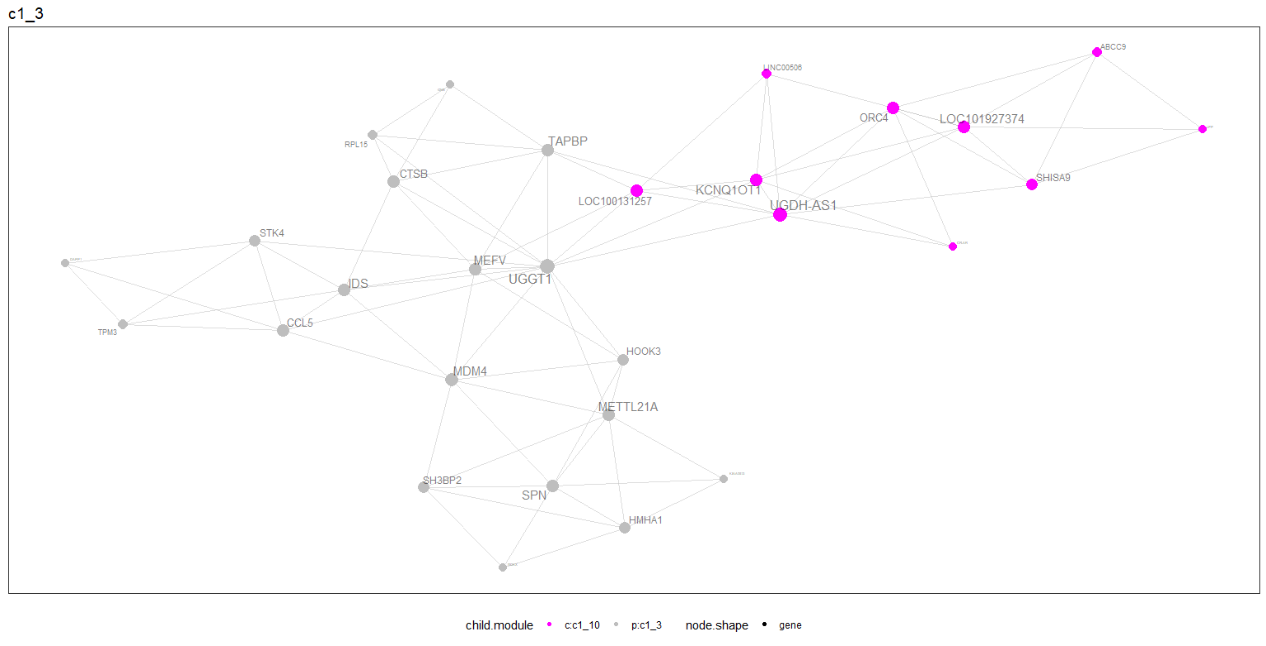

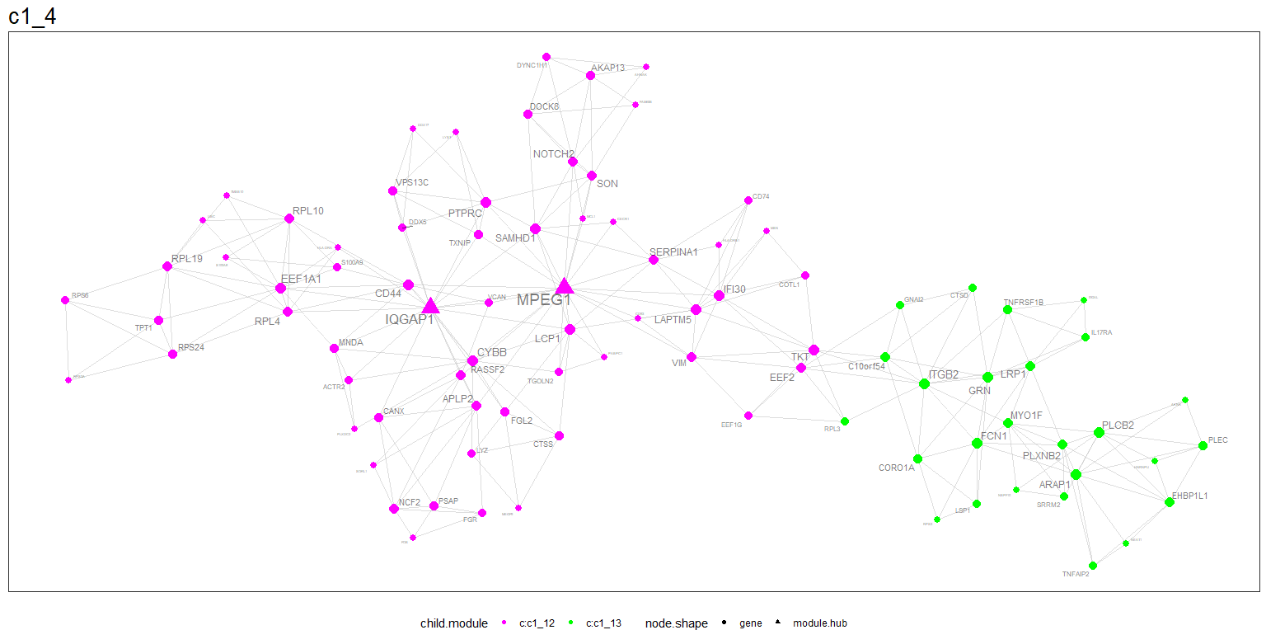

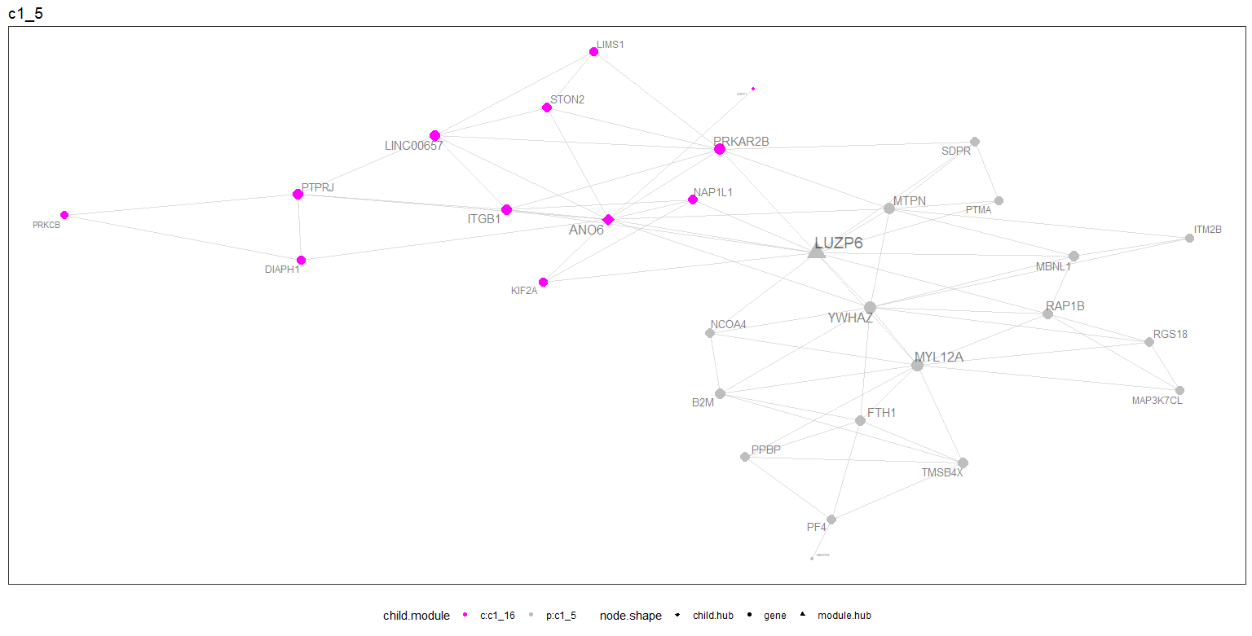


**Figure S2 Co-expression modules (c1_6, c1_12 and c1_16) of identified hub genes with traits associated gene.**


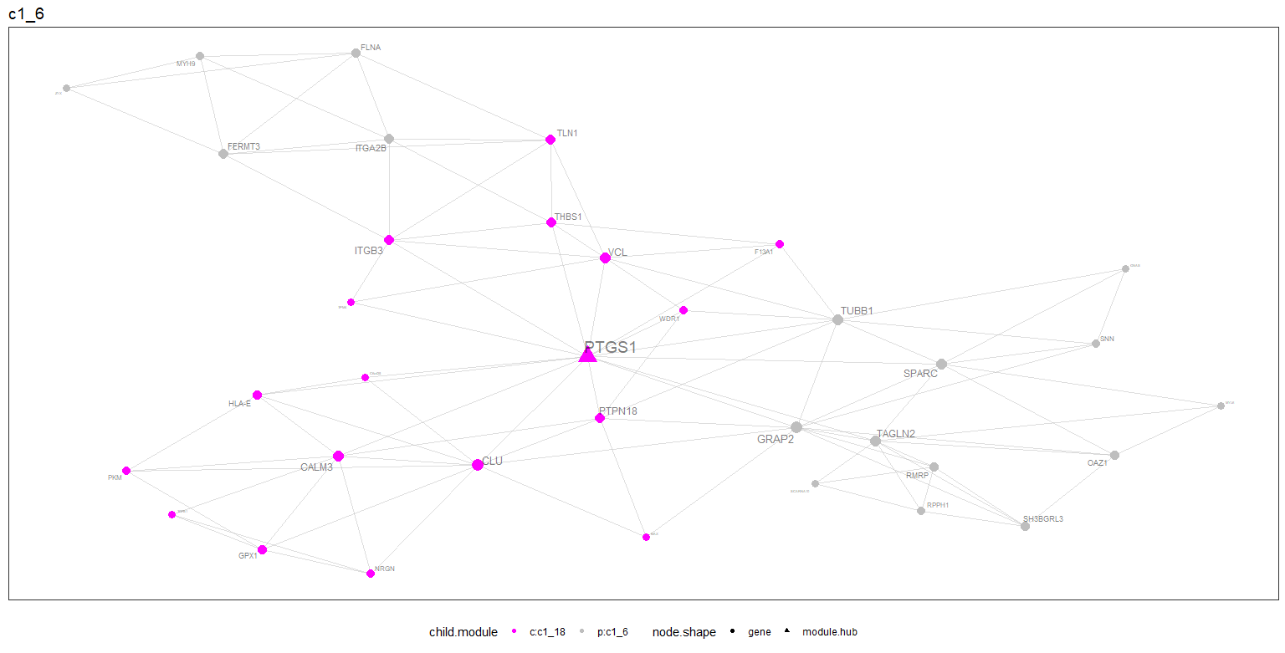

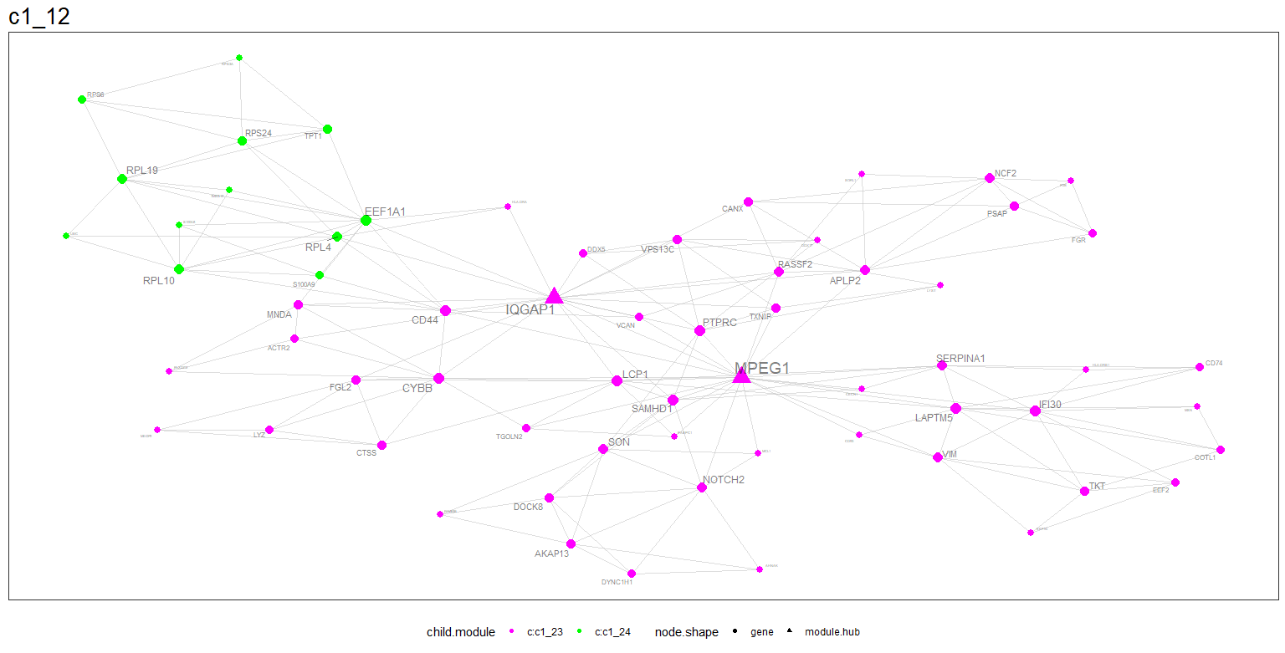

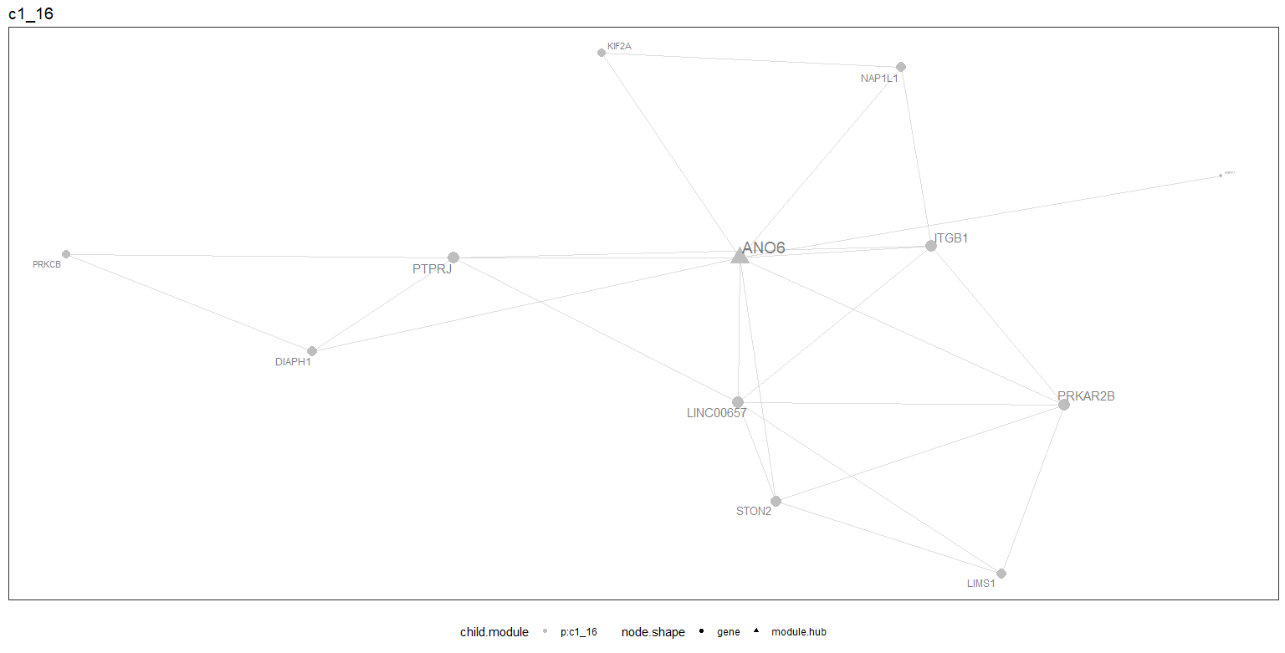


**Figure S3 Co-expression modules (c1_18, c1_23 and c1_25) of identified hub genes with traits associated gene.**


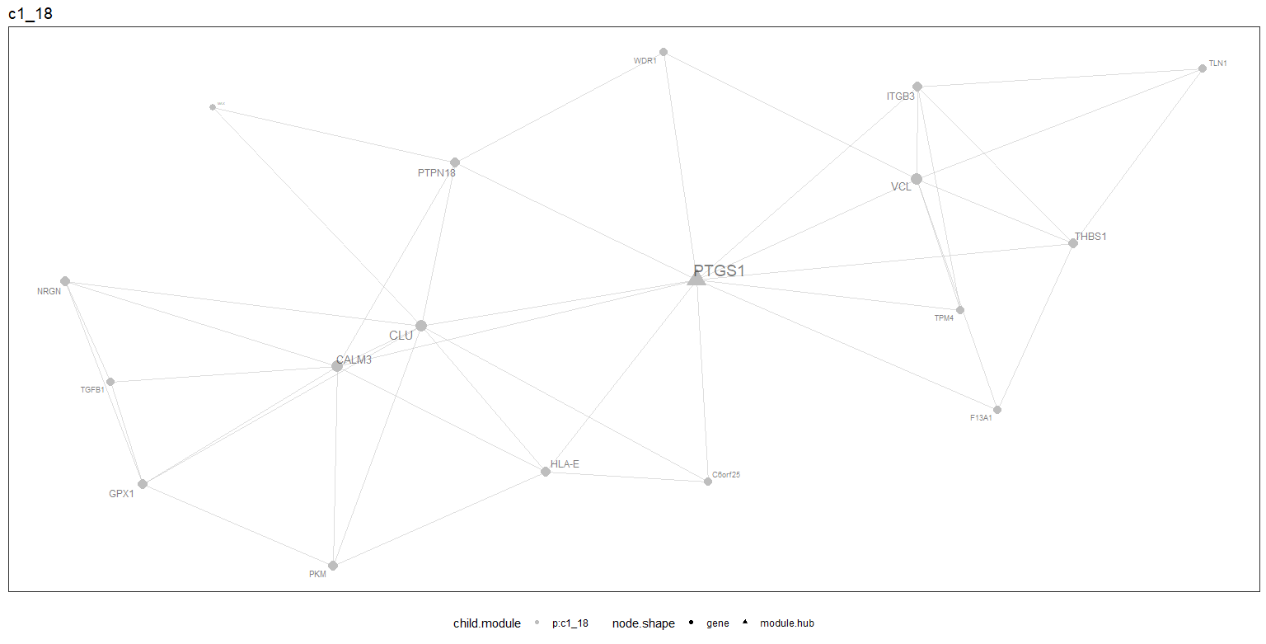

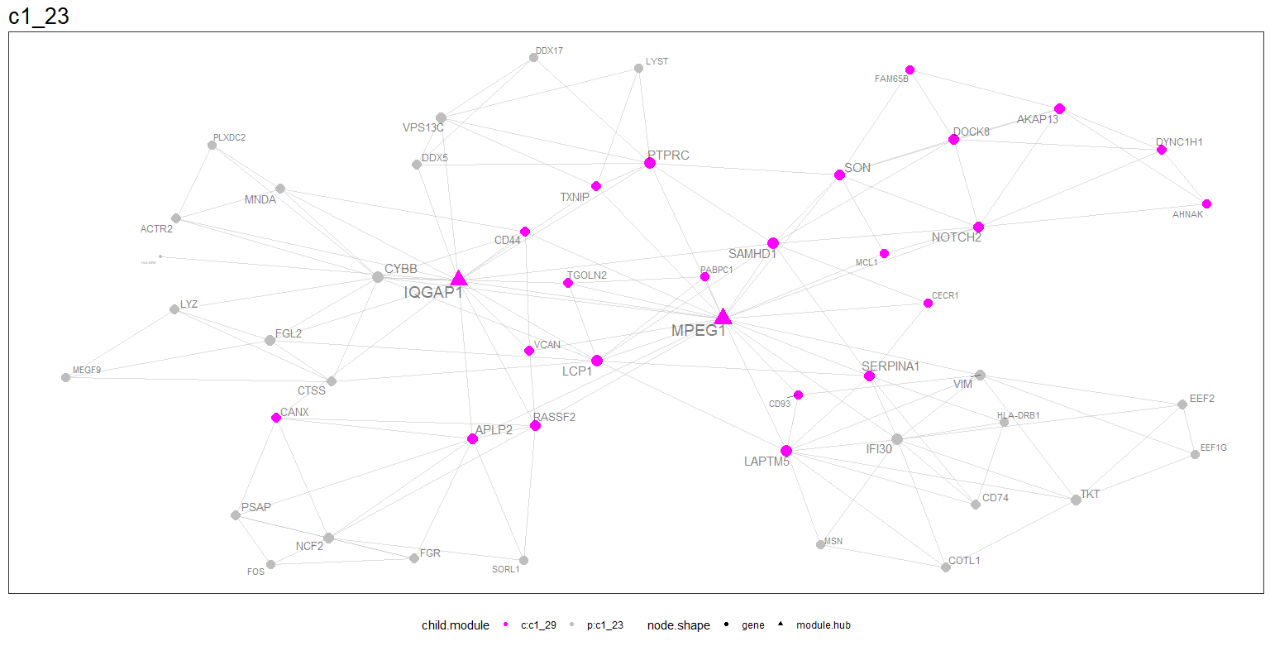

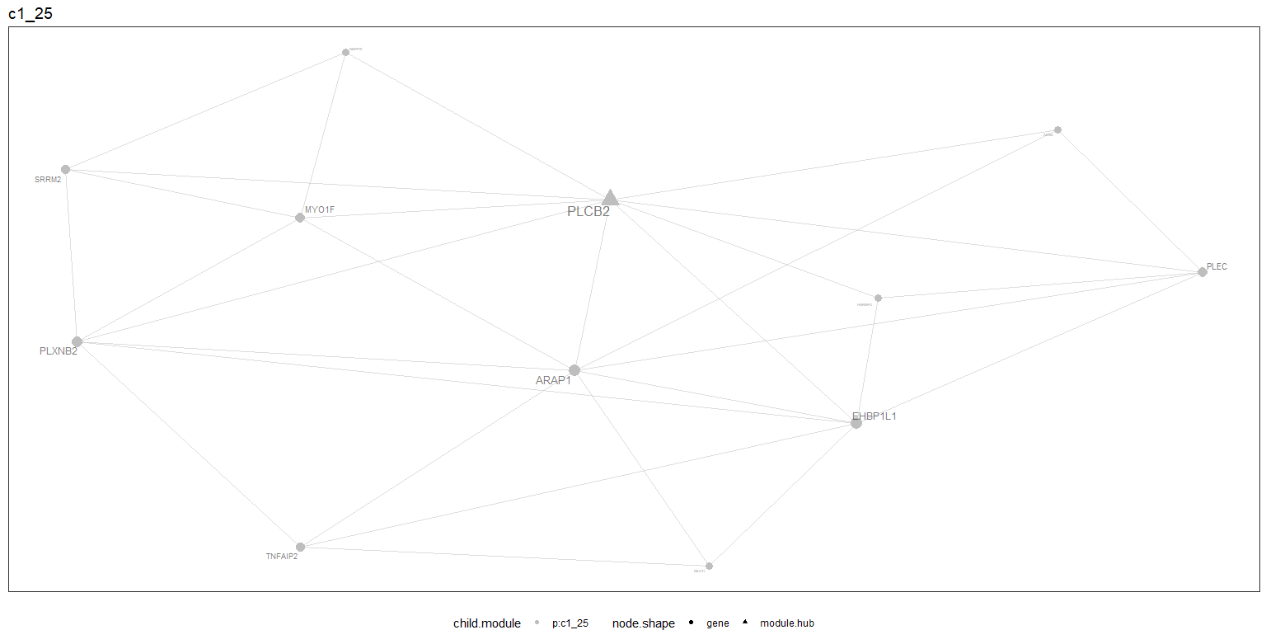


**Figure S4 Co-expression modules (c1_29 and c1_34) of identified hub genes with traits associated gene.**


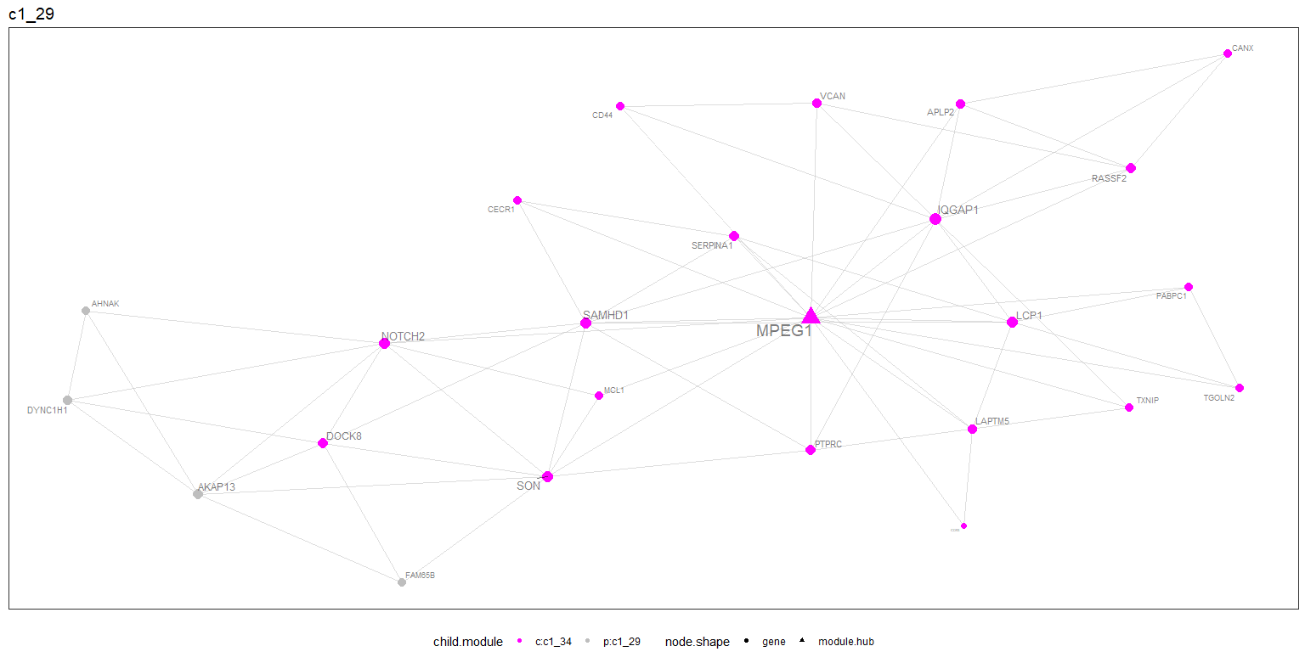

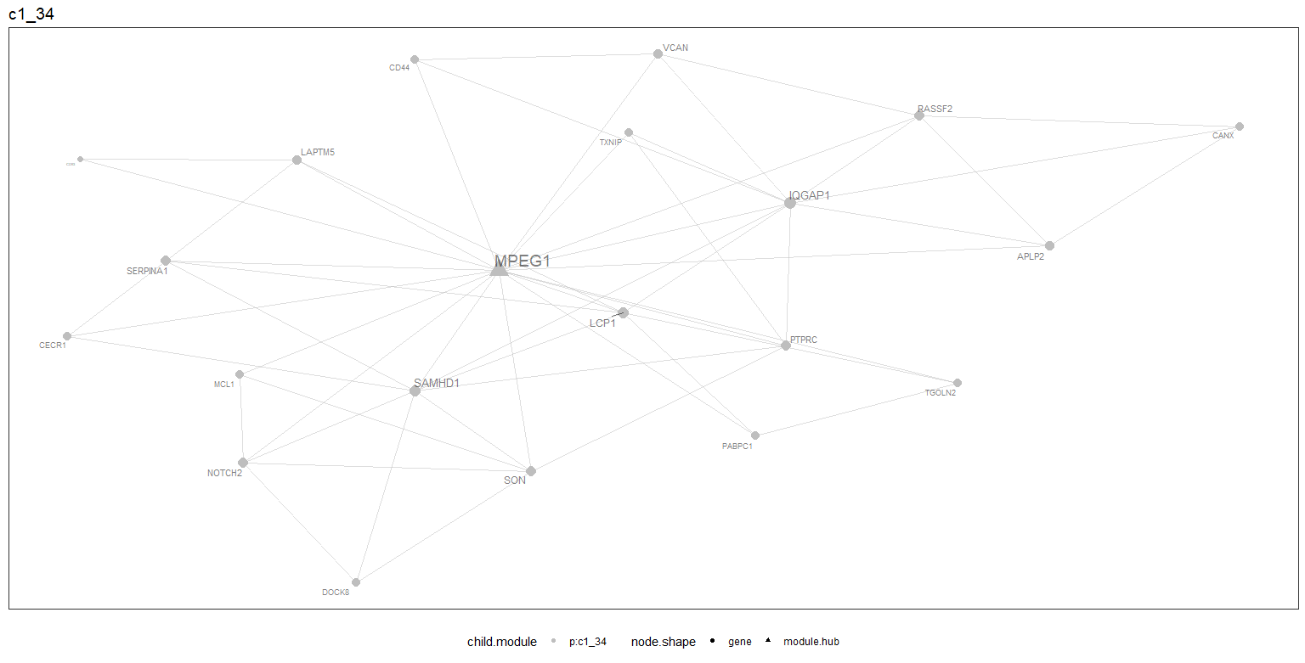


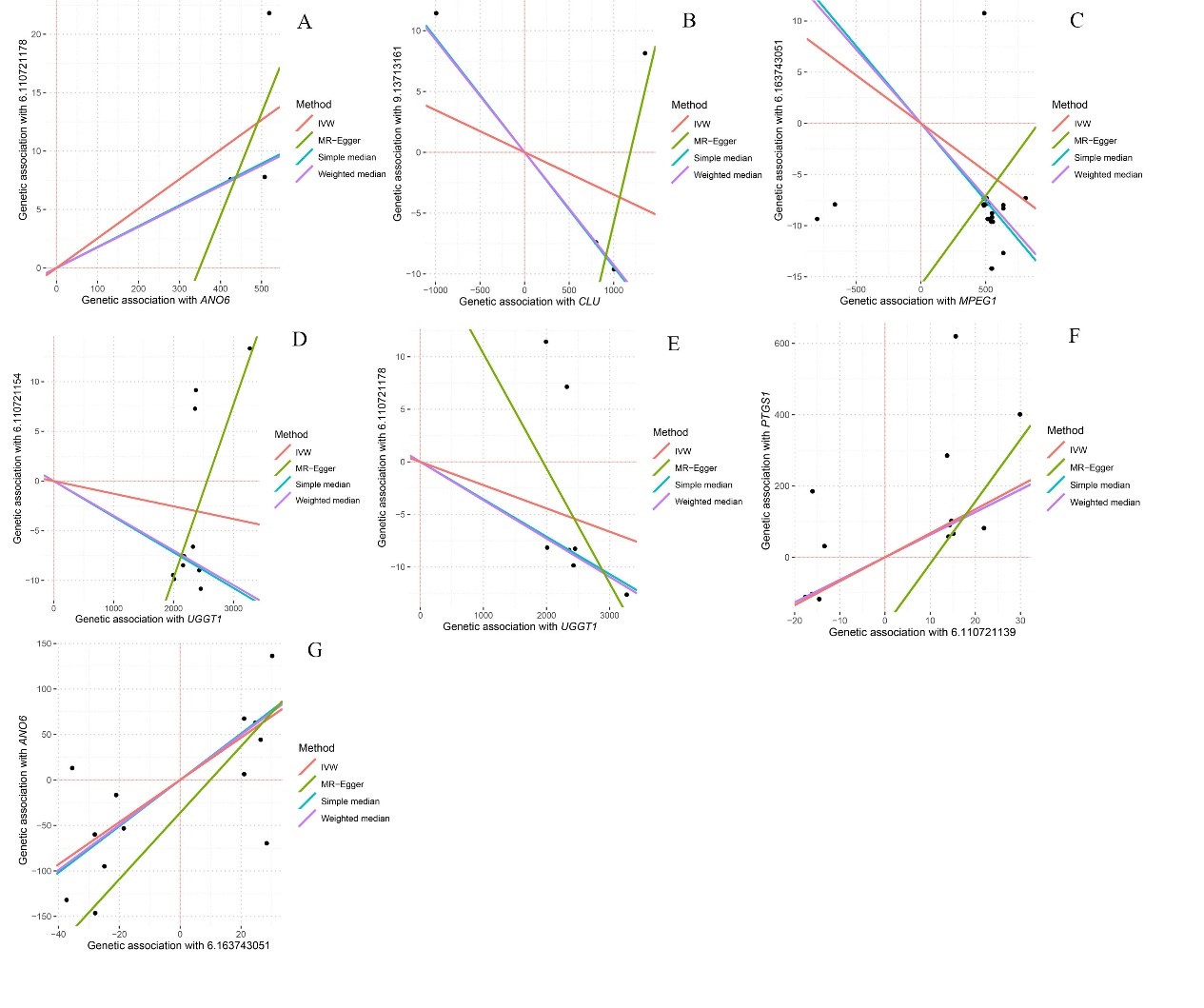


**Figure S5 MR analysis scatter plot: effect of DEGs/DMRs on DMRs/DEGs**


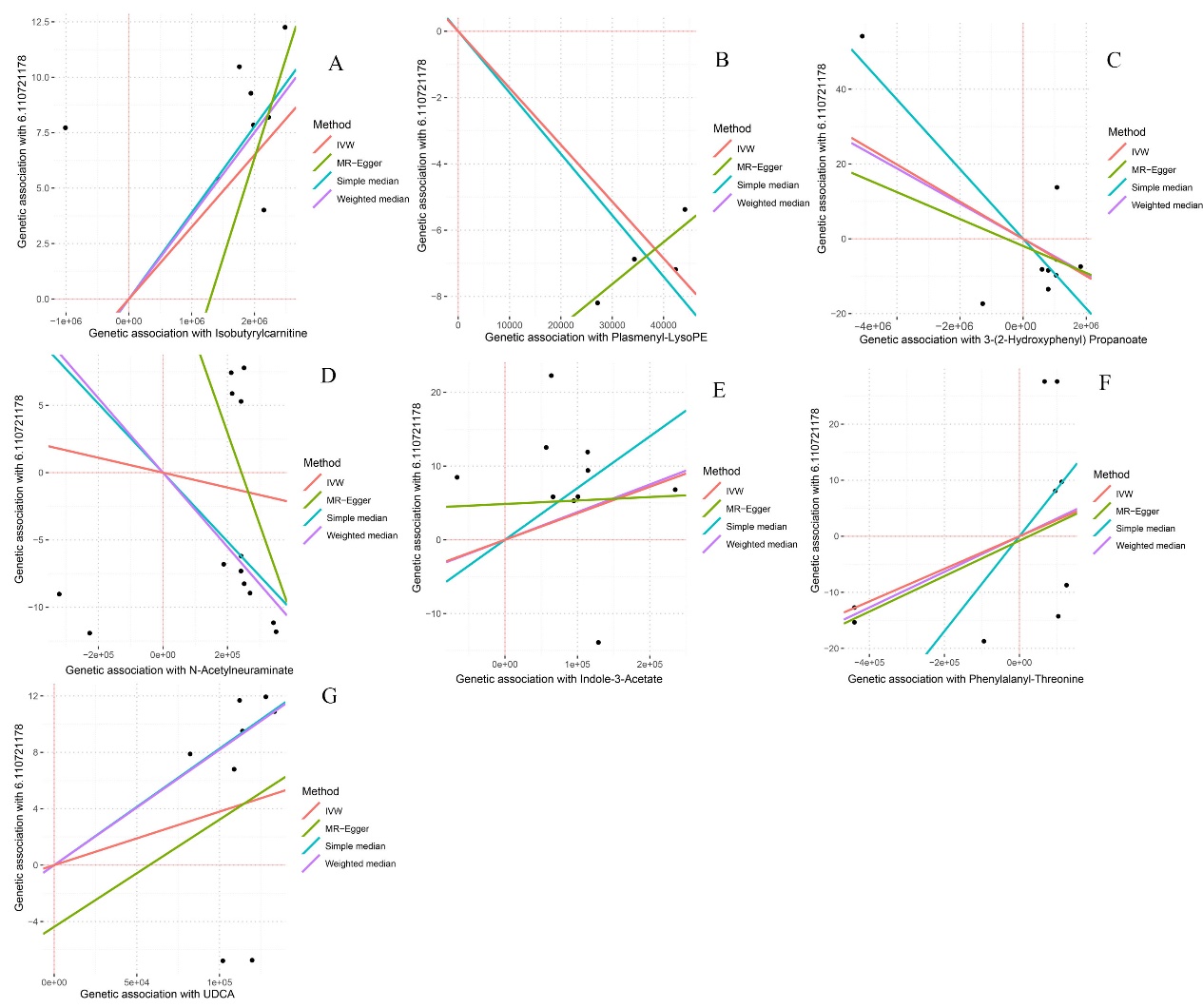


**Figure S6 MR analysis scatter plot: effect of DAMs on DMR 6.110721178.**


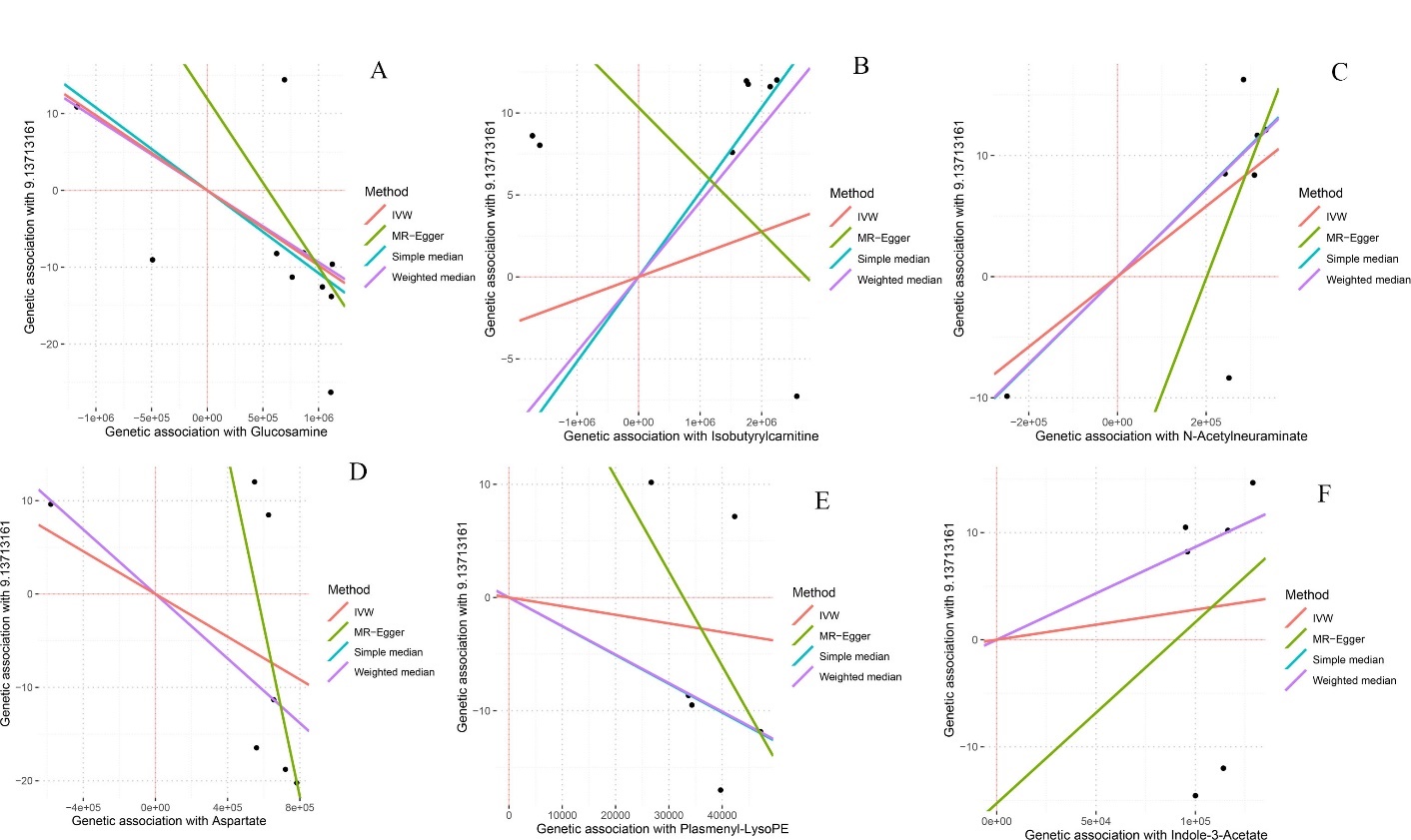


**Figure S7 MR analysis scatter plot: effect of DAMs on DMR 9.13713161.**


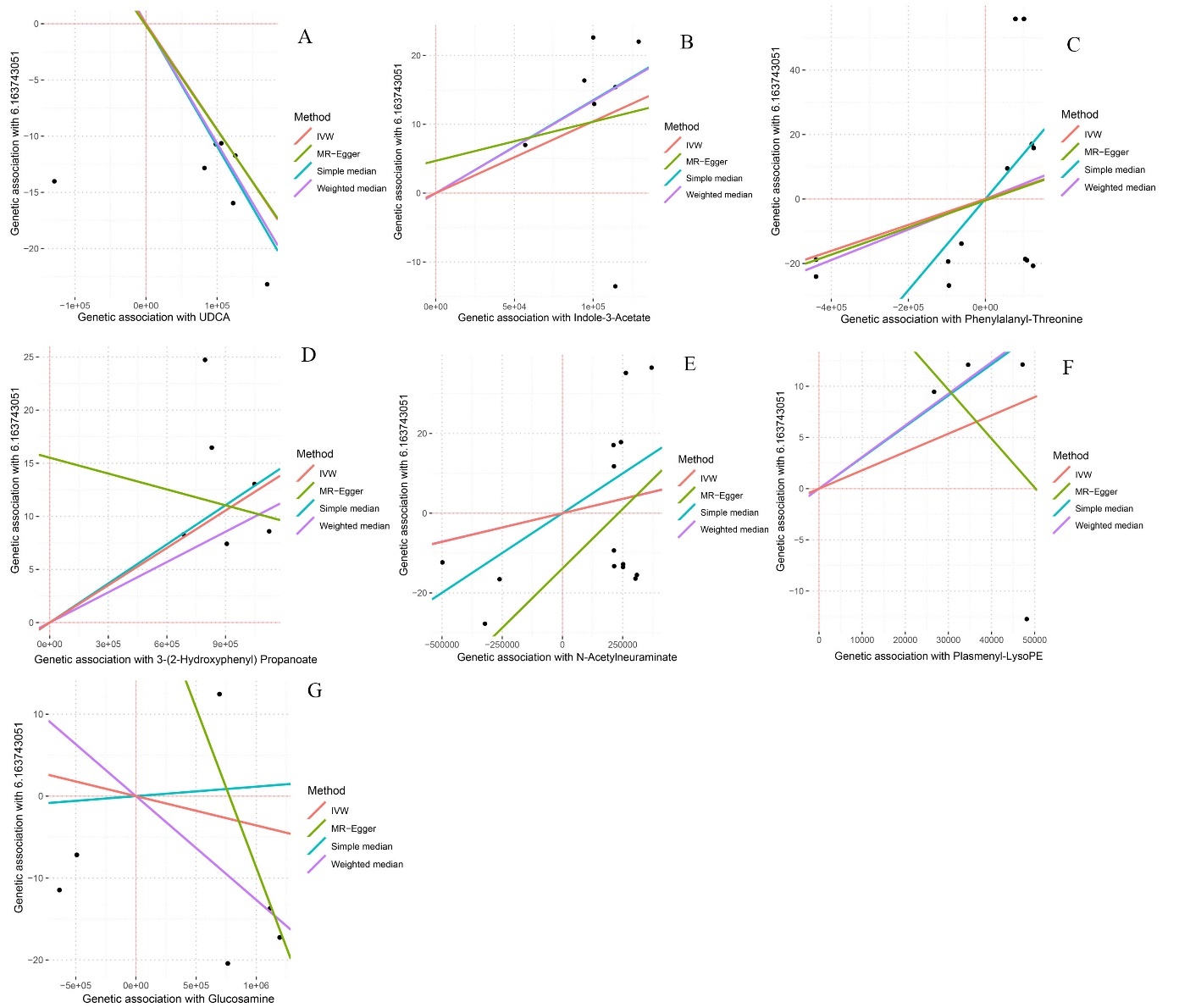


**Figure S8 MR analysis scatter plot: effect of DAMs on DMR 6.163743051.**


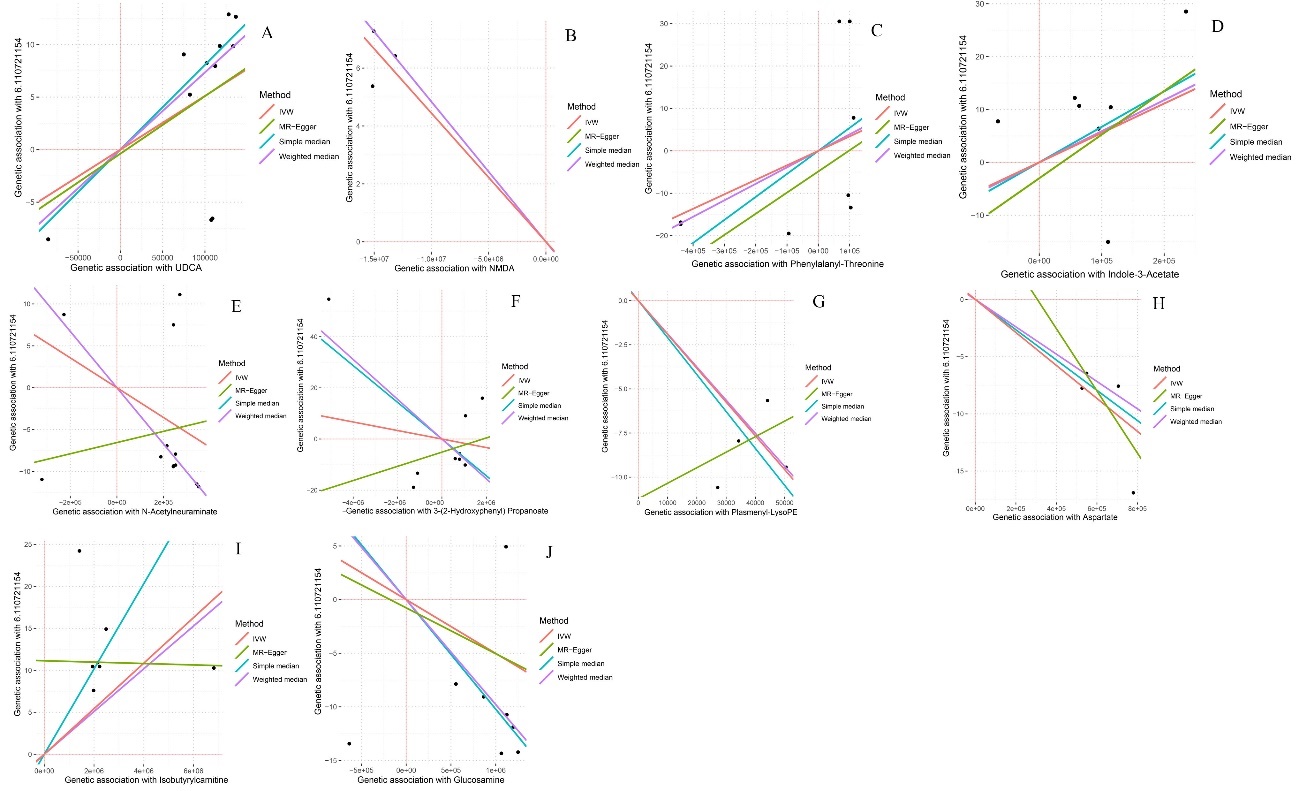


**Figure S9 MR analysis scatter plot: effect of DAMs on DMR 6.110721154.**


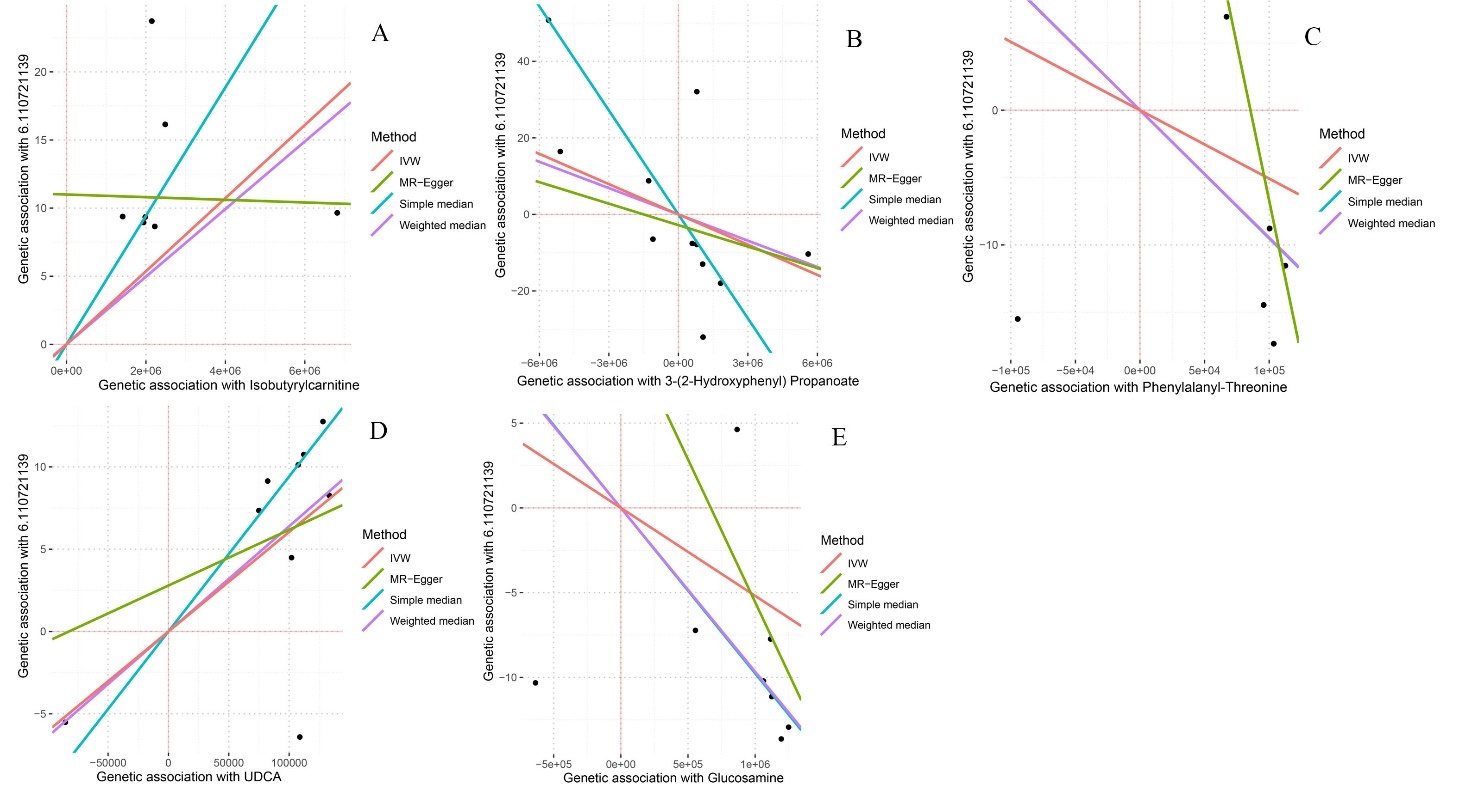


**Figure S10 MR analysis scatter plot: effect of DAMs on DMR 6.110721139.**


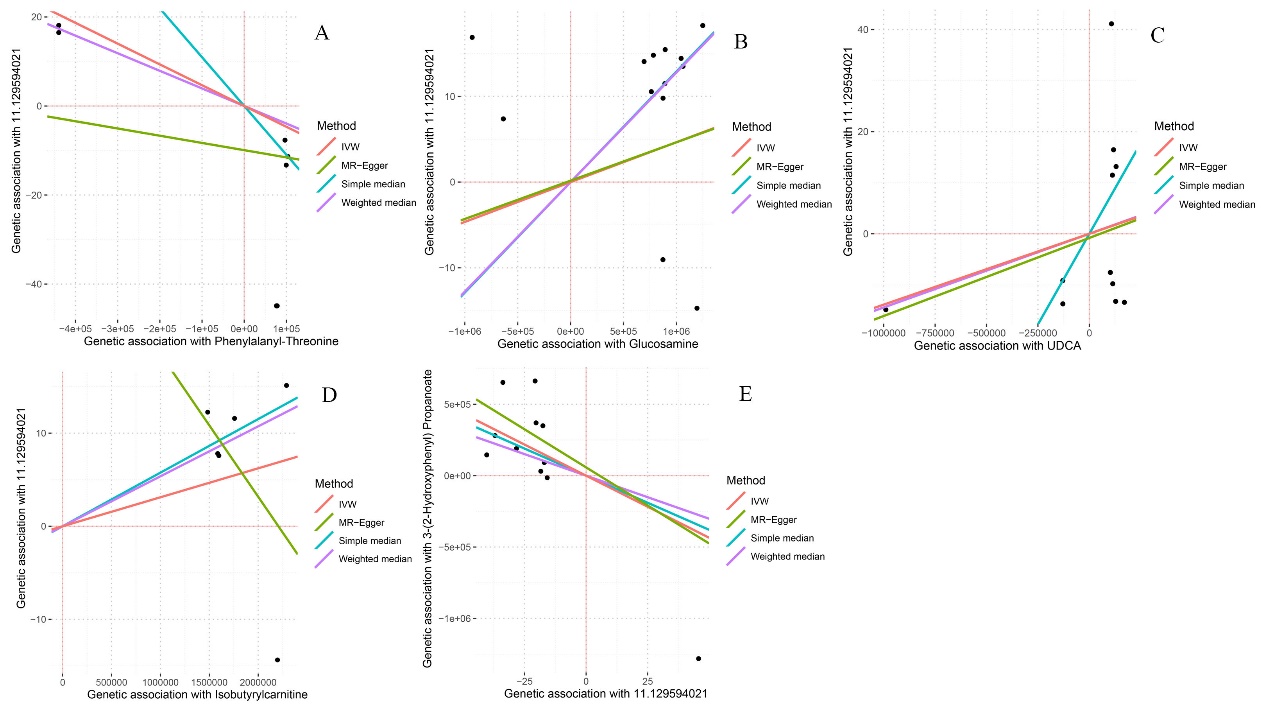


**Figure S11 MR analysis scatter plot: effect of DAMs on DMR 11.129594021.**


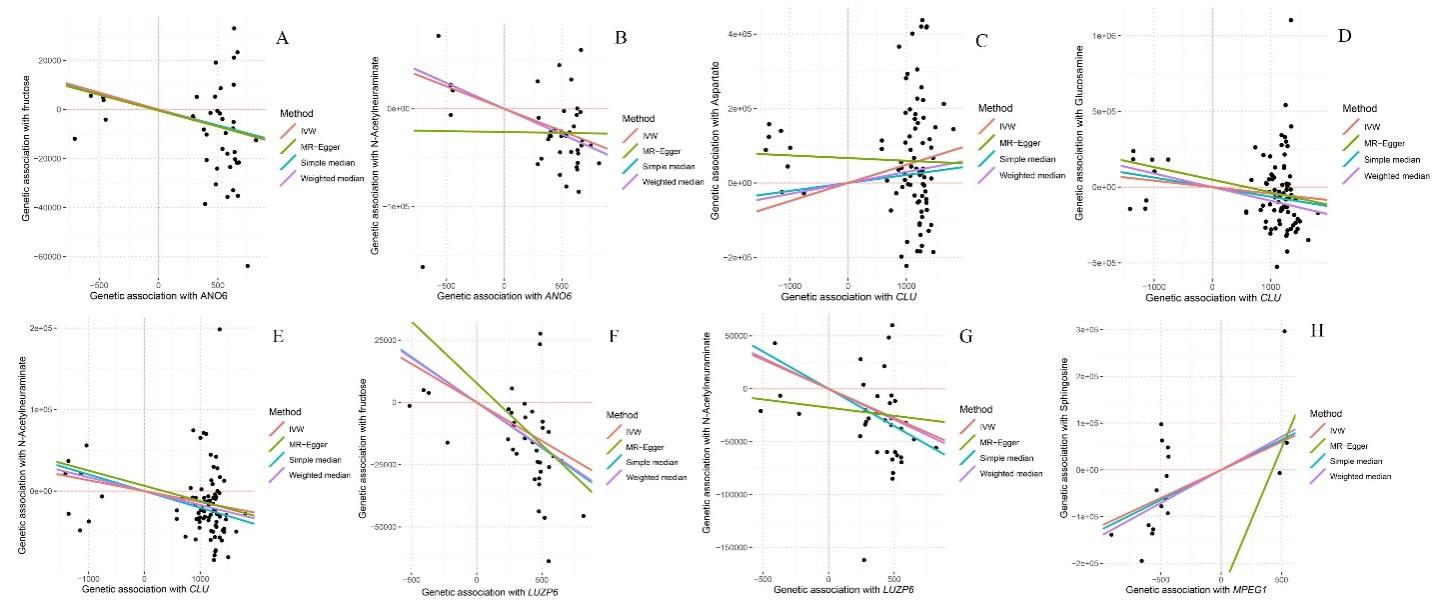


**Figure S12_1 MR analysis scatter plot_1: effect of DEGs on DAMs.**


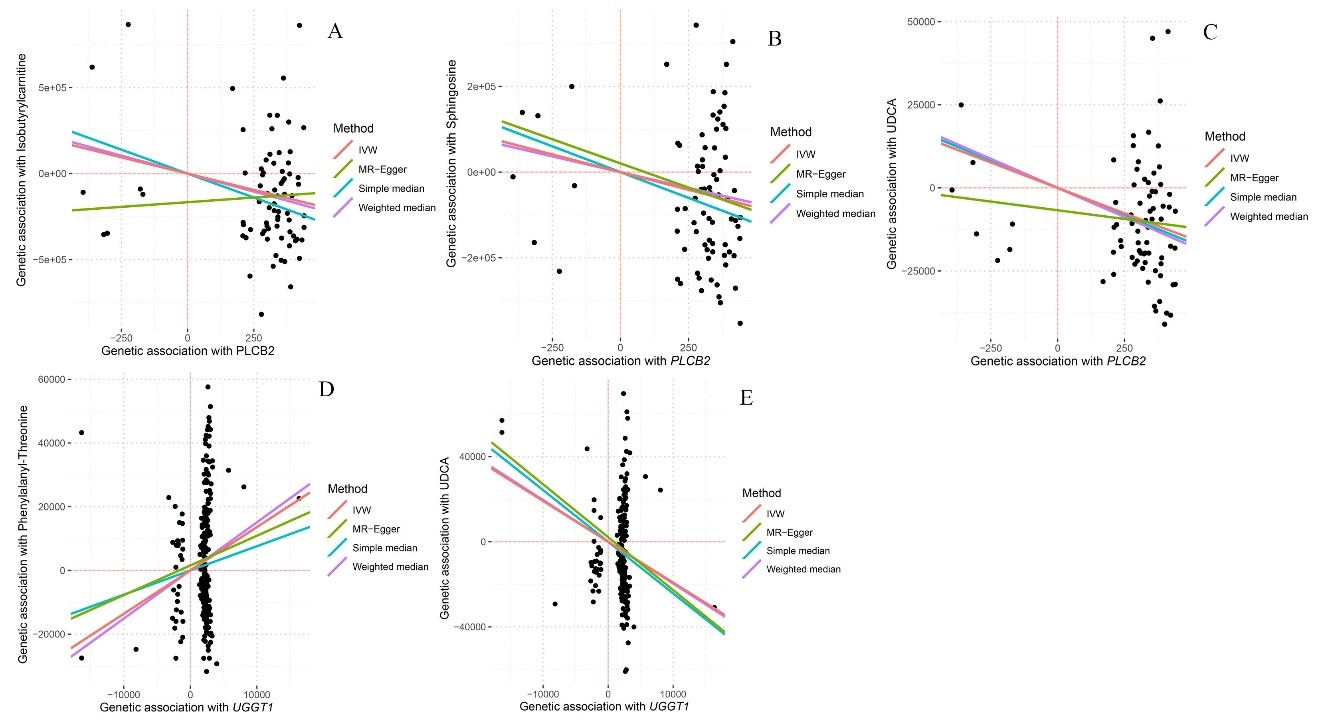


**Figure S12_2 MR analysis scatter plot_2: effect of DEGs on DAMs.**


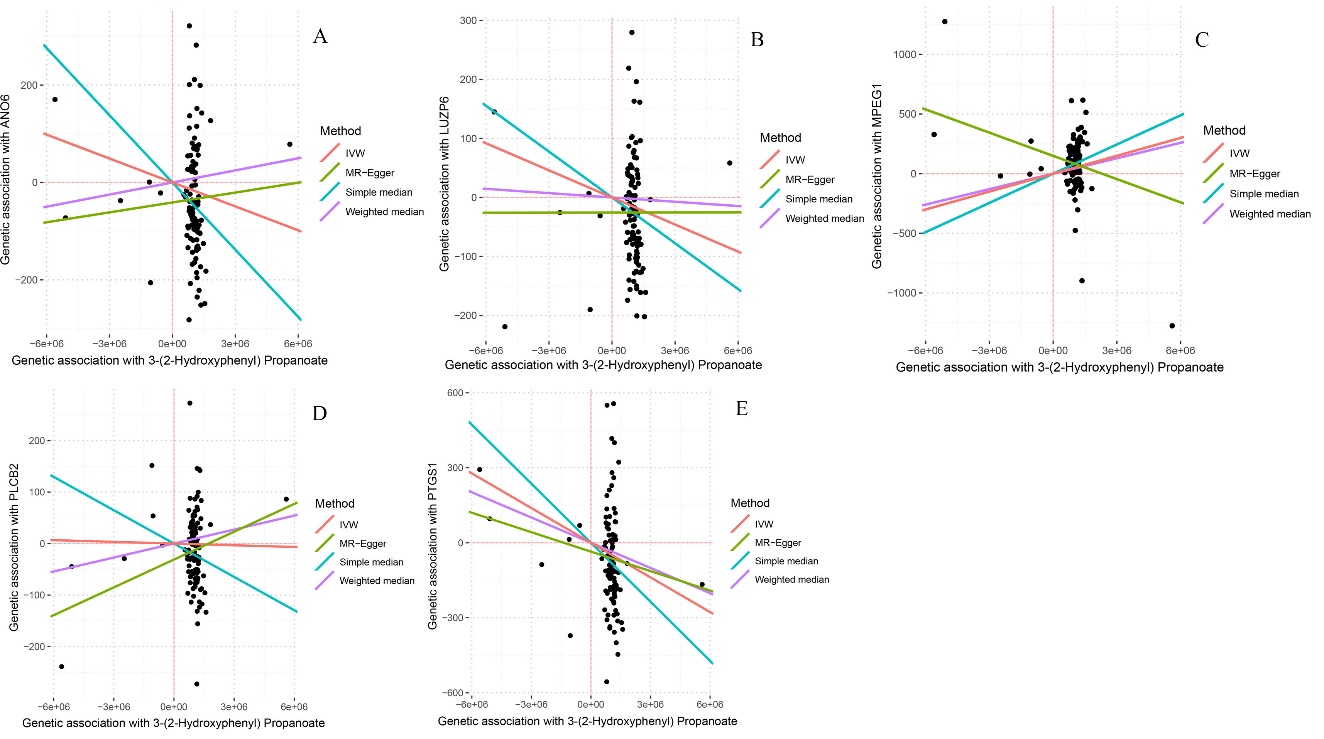


**Figure S13 MR analysis scatter plot: effect of 3-(2-Hydroxyphenyl) Propanoate on DEMs.**


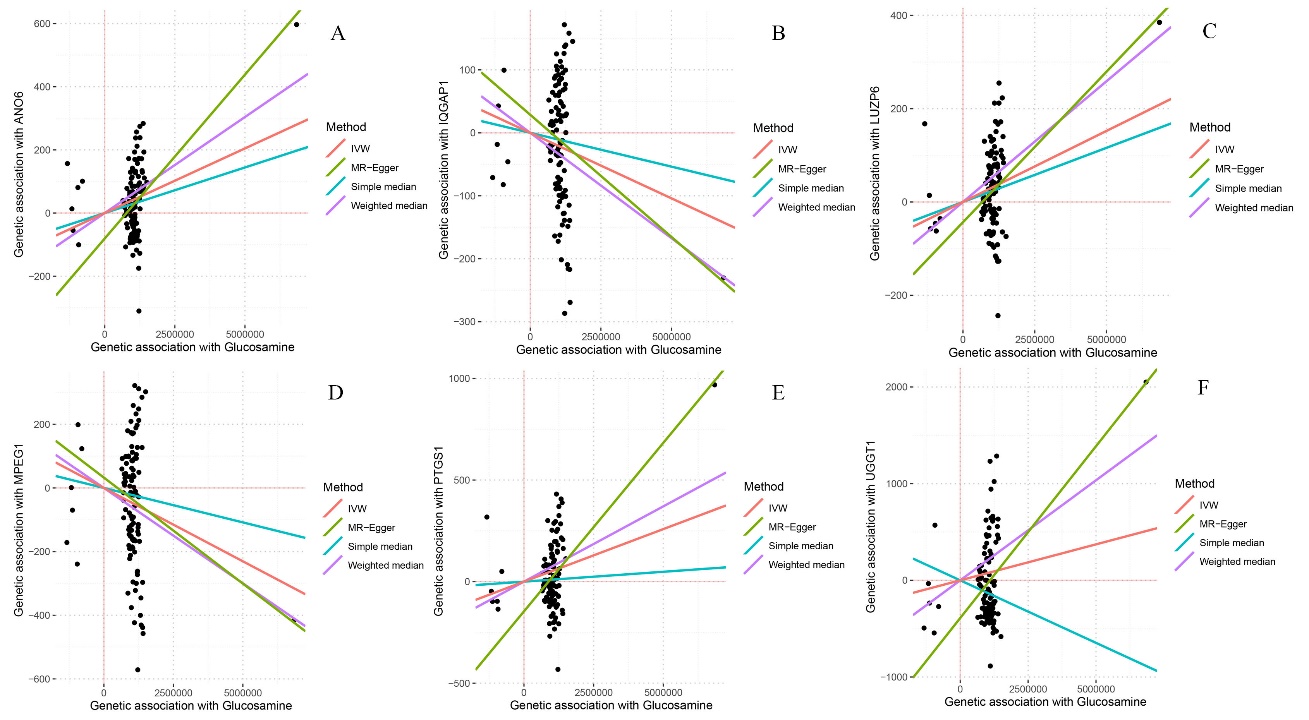


**Figure S14 MR analysis scatter plot: effect of Glucosamine on DEMs.**


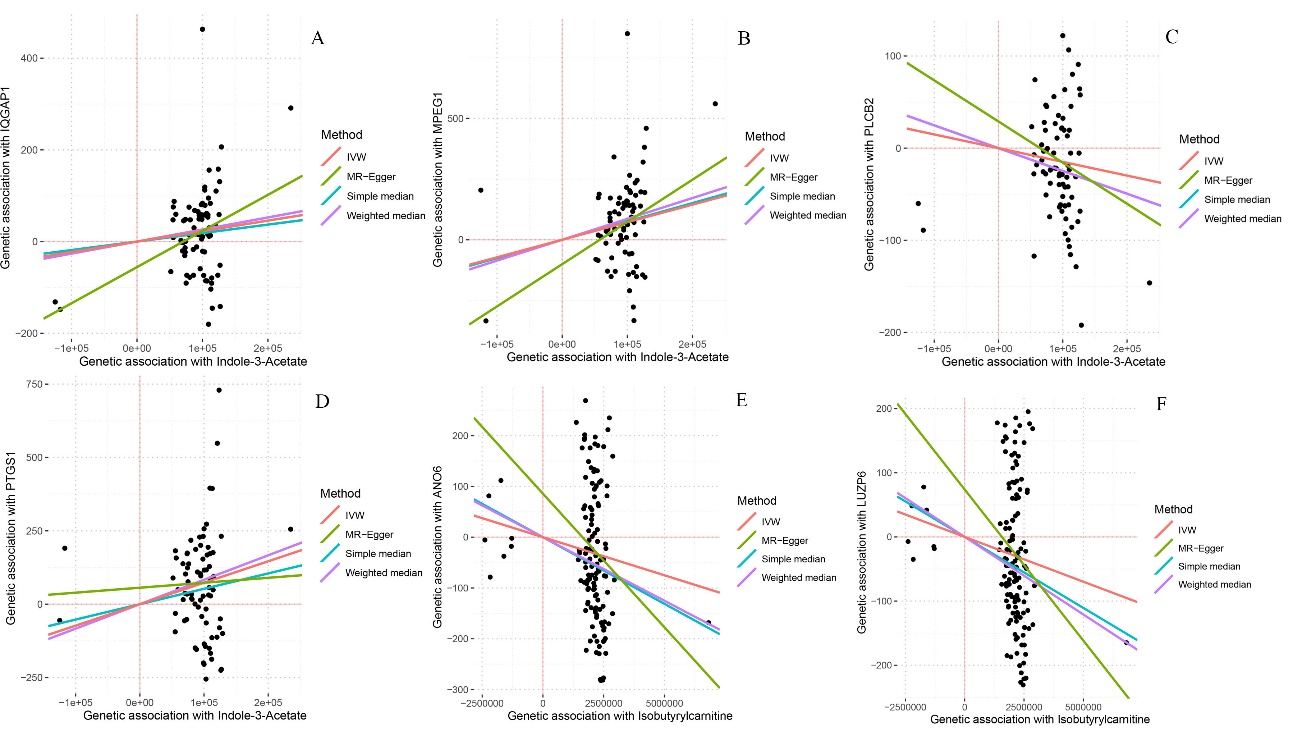


**Figure S15 MR analysis scatter plot: effect of Indole-3-Acetate, Isobutyrylcarnitine on DEMs.**


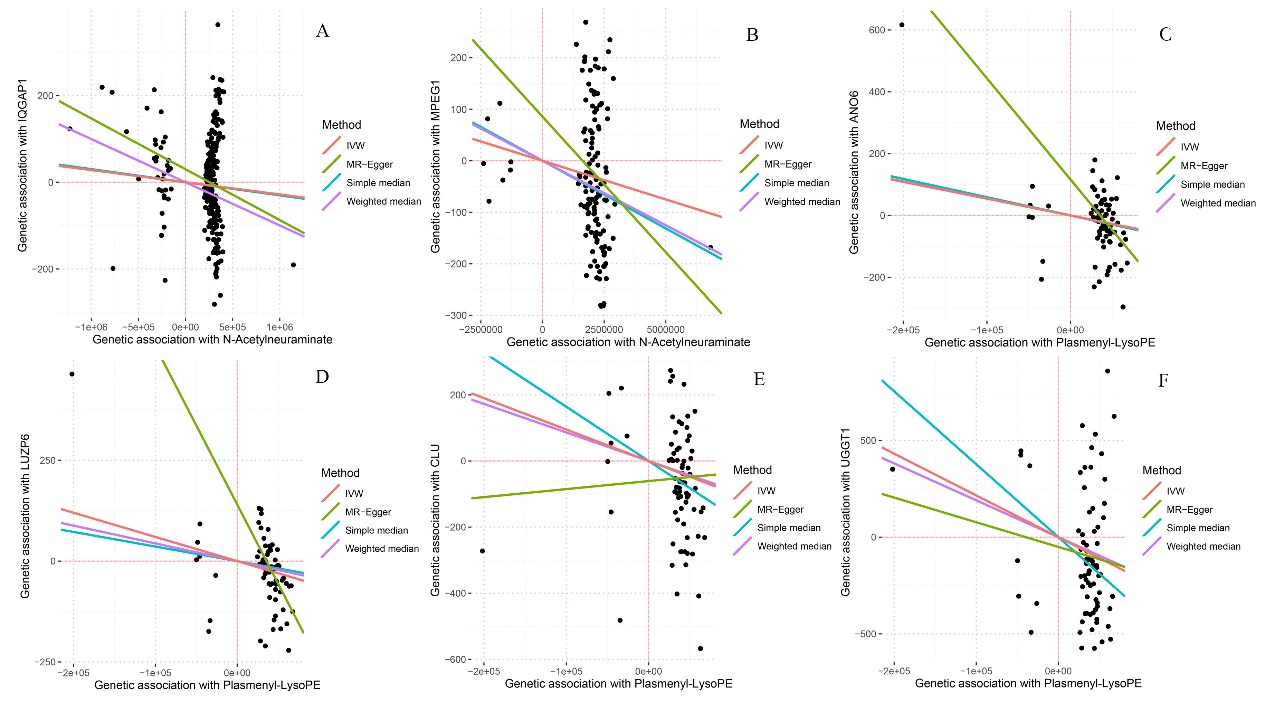


**Figure S16 MR analysis scatter plot: effect of N-Acetylneuraminate, Phenylalanyl-Threonine and UDCA on DEGs.**


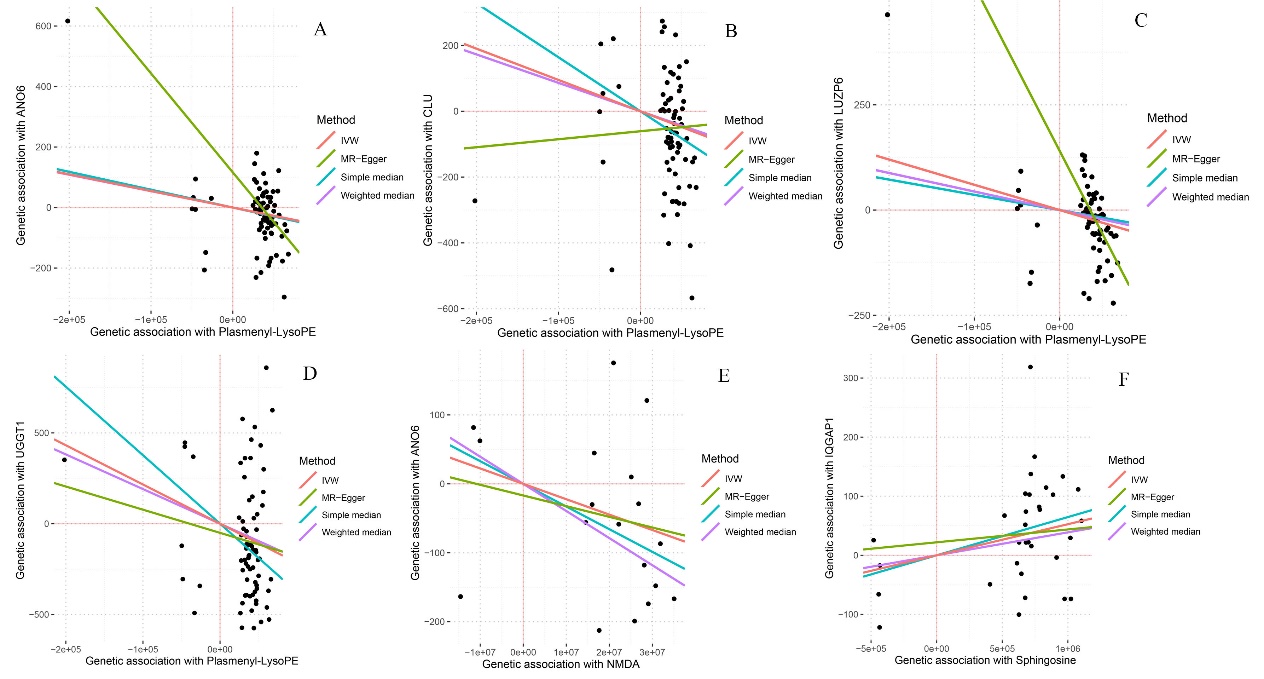


**Figure S17 MR analysis scatter plot: effect of Plasmenyl-LysoPE, NMDA and Sphingosine on DEGs.**
