# Supplementary material for: Integrative analysis of multi-omics data to detect the underlying molecular mechanisms for obesity in vivo in humans
Source: Hum Genomics. 2022 May 14;16:15. doi: 10.1186/s40246-022-00388-x (PMC9107154; doi:10.1186/s40246-022-00388-x)
Supplement: Supplementary file 2 — Additional file 2: Tables S1–S12 in.docx format are included in the supplementary information. [file 40246_2022_388_MOESM2_ESM.docx]

**Legends for the supplementary tables**

Supplementary Table1: Differentially expressed genes

Supplementary Table2: Differentially expressed genes in each gene modules.

Supplementary Table3: GO term enrichment analysis of DEGs.

Supplementary Table4: Differentially methylated regions (both hyper- and hypo- methylated)

Supplementary Table5: Differentially hyper-methylated regions

Supplementary Table6: Differentially hypo-methylated regions

Supplementary Table7: Differentially methylated regions and their nearest genes

Supplementary Table8: DAVID enrichment analysis of the genes DAMs annoted to.

Supplementary Table9: Enrichment analysis of the metabolites.

Supplementary Table10: Causal pairs between hub genes and methylation.

Supplementary Table11: Causal pairs between differentially methylated regions and differentially expressed metabolites

Supplementary Table12: Causal pairs between hub genes and differentially expressed metabolites

**Supplementary Table1: Differentially expressed genes**

| **ID** | **Gene_symbol** | **logFC** | **AveExpr** | **t** | **P.Value** | **adj.P.Val** | **B** |
| --- | --- | --- | --- | --- | --- | --- | --- |
| 12482 | MALAT1 | 8.565041 | 13.35448 | 31.58791 | 3.34E-77 | 1.87E-74 | 165.6794 |
| 17442 | PLCG2 | 8.352004 | 13.09898 | 30.72943 | 2.60E-75 | 4.66E-74 | 161.3444 |
| 216 | ACTB | 8.121712 | 12.69235 | 31.17318 | 2.71E-76 | 1.93E-74 | 163.5954 |
| 12388 | LYZ | 8.109582 | 12.7015 | 31.07673 | 4.42E-76 | 2.24E-74 | 163.108 |
| 476 | AHNAK | 7.971615 | 12.44292 | 31.57227 | 3.62E-77 | 1.87E-74 | 165.6011 |
| 22931 | TMSB4X | 7.76894 | 12.16088 | 30.04479 | 8.85E-74 | 4.89E-73 | 157.8277 |
| 5363 | EEF1A1 | 7.664963 | 11.96732 | 31.18362 | 2.57E-76 | 1.93E-74 | 163.6481 |
| 1454 | B2M | 7.663816 | 11.98519 | 29.9607 | 1.37E-73 | 6.96E-73 | 157.392 |
| 6639 | FLNA | 7.536263 | 11.77752 | 31.16713 | 2.79E-76 | 1.95E-74 | 163.5649 |
| 19343 | RPPH1 | 7.472441 | 11.68279 | 31.00828 | 6.26E-76 | 2.56E-74 | 162.7615 |
| 24039 | VCAN | 7.455801 | 11.63892 | 30.73055 | 2.58E-75 | 4.66E-74 | 161.3501 |
| 18176 | PSAP | 7.405563 | 11.58065 | 31.18272 | 2.58E-76 | 1.93E-74 | 163.6436 |
| 15141 | MYH9 | 7.317223 | 11.43192 | 31.28529 | 1.54E-76 | 1.87E-74 | 164.1606 |
| 22558 | TLN1 | 7.256421 | 11.33774 | 30.86344 | 1.31E-75 | 3.38E-74 | 162.0265 |
| 4305 | CTSS | 7.251576 | 11.37151 | 30.72114 | 2.71E-75 | 4.78E-74 | 161.3021 |
| 6821 | FTH1 | 7.237625 | 11.33745 | 30.10973 | 6.32E-74 | 3.76E-73 | 158.1635 |
| 23783 | UGDH-AS1 | 7.121388 | 11.14168 | 30.17154 | 4.59E-74 | 2.97E-73 | 158.4827 |
| 8141 | HLA-B | 7.115253 | 11.12826 | 31.27527 | 1.62E-76 | 1.87E-74 | 164.1102 |
| 9189 | KCNQ1OT1 | 7.113045 | 11.12682 | 30.59974 | 5.04E-75 | 6.84E-74 | 160.6823 |
| 17753 | PPBP | 7.09603 | 11.10792 | 28.48618 | 3.34E-70 | 6.47E-70 | 149.6193 |
| 8158 | HLA-E | 7.029347 | 10.99398 | 31.2926 | 1.48E-76 | 1.87E-74 | 164.1974 |
| 23560 | TUBB1 | 6.963352 | 10.88769 | 29.16476 | 8.94E-72 | 2.40E-71 | 153.228 |
| 6825 | FTL | 6.960953 | 10.89515 | 31.00191 | 6.46E-76 | 2.57E-74 | 162.7292 |
| 3087 | CD74 | 6.800591 | 10.62523 | 31.08333 | 4.27E-76 | 2.23E-74 | 163.1414 |
| 19048 | RMRP | 6.758268 | 10.51469 | 31.46373 | 6.24E-77 | 1.87E-74 | 165.0574 |
| 6425 | FCN1 | 6.755038 | 10.56585 | 31.17873 | 2.63E-76 | 1.93E-74 | 163.6234 |
| 19547 | S100A9 | 6.742375 | 10.52534 | 30.17582 | 4.49E-74 | 2.93E-73 | 158.5048 |
| 10154 | LINC00506 | 6.700639 | 10.48105 | 30.30659 | 2.28E-74 | 1.80E-73 | 159.1787 |
| 2945 | CCL5 | 6.688984 | 10.50025 | 28.3827 | 5.83E-70 | 1.08E-69 | 149.0641 |
| 15866 | NRGN | 6.68673 | 10.46193 | 29.41226 | 2.42E-72 | 7.69E-72 | 154.5307 |
| 16671 | PABPC1 | 6.675129 | 10.45066 | 30.85554 | 1.36E-75 | 3.43E-74 | 161.9864 |
| 12172 | LRP1 | 6.649908 | 10.3906 | 31.20267 | 2.33E-76 | 1.89E-74 | 163.7442 |
| 9838 | LCP1 | 6.647434 | 10.38566 | 30.78625 | 1.94E-75 | 4.11E-74 | 161.6339 |
| 5872 | F13A1 | 6.635864 | 10.37301 | 28.84931 | 4.78E-71 | 1.09E-70 | 151.5572 |
| 23132 | TPT1 | 6.624947 | 10.36876 | 30.41005 | 1.34E-74 | 1.24E-73 | 159.7105 |
| 8140 | HLA-A | 6.617347 | 10.35535 | 31.0247 | 5.76E-76 | 2.48E-74 | 162.8447 |
| 19208 | RNVU1-19 | 6.58804 | 10.30402 | 28.21737 | 1.42E-69 | 2.48E-69 | 148.1745 |
| 8965 | ITGB2 | 6.579679 | 10.26775 | 31.44481 | 6.87E-77 | 1.87E-74 | 164.9625 |
| 8142 | HLA-C | 6.516663 | 10.18701 | 30.99529 | 6.68E-76 | 2.57E-74 | 162.6957 |
| 19136 | RNF213 | 6.494683 | 10.13508 | 31.7736 | 1.32E-77 | 1.87E-74 | 166.6063 |
| 14946 | MSN | 6.484494 | 10.13834 | 30.99867 | 6.57E-76 | 2.57E-74 | 162.7128 |
| 219 | ACTG1 | 6.474662 | 10.11986 | 30.85332 | 1.38E-75 | 3.45E-74 | 161.9751 |
| 23619 | TXNIP | 6.471456 | 10.08263 | 31.11284 | 3.68E-76 | 2.17E-74 | 163.2906 |
| 8967 | ITGB3 | 6.469365 | 10.10612 | 29.1071 | 1.21E-71 | 3.14E-71 | 152.9235 |
| 19786 | SDPR | 6.425587 | 10.05666 | 28.5866 | 1.95E-70 | 3.94E-70 | 150.1567 |
| 4403 | CYBB | 6.42518 | 10.01887 | 30.29514 | 2.42E-74 | 1.87E-73 | 159.1198 |
| 15372 | NCOA4 | 6.421762 | 10.02481 | 29.8777 | 2.11E-73 | 9.82E-73 | 156.9612 |
| 24040 | VCL | 6.405369 | 10.01952 | 29.42604 | 2.25E-72 | 7.22E-72 | 154.6031 |
| 15457 | NEAT1 | 6.399102 | 10.00824 | 30.66085 | 3.69E-75 | 5.71E-74 | 160.9945 |
| 5371 | EEF2 | 6.393822 | 9.989991 | 31.21085 | 2.24E-76 | 1.88E-74 | 163.7855 |
| 14760 | MPEG1 | 6.382166 | 9.969781 | 30.17762 | 4.44E-74 | 2.92E-73 | 158.5141 |
| 18318 | PTMA | 6.379148 | 10.00809 | 30.46017 | 1.03E-74 | 1.06E-73 | 159.9677 |
| 17382 | PKM | 6.345451 | 9.928634 | 30.60939 | 4.80E-75 | 6.68E-74 | 160.7316 |
| 24479 | YWHAZ | 6.341115 | 9.91421 | 29.62362 | 7.97E-73 | 2.99E-72 | 155.6376 |
| 10245 | LINC00657 | 6.295354 | 9.86569 | 30.32455 | 2.08E-74 | 1.69E-73 | 159.2711 |
| 17457 | PLEC | 6.292075 | 9.826517 | 30.99706 | 6.62E-76 | 2.57E-74 | 162.7046 |
| 15318 | NBPF10 | 6.248117 | 9.733478 | 30.91916 | 9.85E-76 | 2.97E-74 | 162.3095 |
| 21607 | SRRM2 | 6.24477 | 9.737964 | 31.79628 | 1.18E-77 | 1.87E-74 | 166.7193 |
| 24072 | VIM | 6.241896 | 9.740544 | 31.07052 | 4.56E-76 | 2.27E-74 | 163.0766 |
| 11186 | LOC101927374 | 6.23543 | 9.760569 | 29.26246 | 5.33E-72 | 1.52E-71 | 153.7431 |
| 18360 | PTPRJ | 6.226051 | 9.739465 | 30.8531 | 1.38E-75 | 3.45E-74 | 161.974 |
| 23107 | TPM3 | 6.178405 | 9.676031 | 30.82158 | 1.62E-75 | 3.72E-74 | 161.8137 |
| 18351 | PTPRC | 6.177065 | 9.63329 | 30.45949 | 1.04E-74 | 1.06E-73 | 159.9642 |
| 24391 | XIST | 6.158896 | 9.620292 | 30.05566 | 8.37E-74 | 4.68E-73 | 157.8839 |
| 3948 | COTL1 | 6.151521 | 9.615507 | 30.93884 | 8.91E-76 | 2.87E-74 | 162.4094 |
| 22069 | TAGLN2 | 6.145708 | 9.620393 | 29.59637 | 9.19E-73 | 3.38E-72 | 155.4952 |
| 7313 | GNAS | 6.145442 | 9.61356 | 30.33946 | 1.93E-74 | 1.59E-73 | 159.3478 |
| 15736 | NOTCH2 | 6.142593 | 9.590052 | 31.19963 | 2.37E-76 | 1.89E-74 | 163.7289 |
| 23668 | UBC | 6.134505 | 9.573838 | 31.01244 | 6.13E-76 | 2.54E-74 | 162.7826 |
| 6521 | FGL2 | 6.131245 | 9.604199 | 30.13329 | 5.59E-74 | 3.45E-73 | 158.2853 |
| 6997 | GAPDH | 6.119799 | 9.558438 | 30.55976 | 6.19E-75 | 7.69E-74 | 160.4778 |
| 12386 | LYST | 6.117686 | 9.562884 | 31.35458 | 1.08E-76 | 1.87E-74 | 164.5093 |
| 18937 | RGS18 | 6.112841 | 9.559631 | 27.51428 | 6.55E-68 | 9.46E-68 | 144.3547 |
| 8154 | HLA-DRA | 6.103309 | 9.535573 | 30.07398 | 7.61E-74 | 4.33E-73 | 157.9787 |
| 9775 | LAPTM5 | 6.101463 | 9.538615 | 30.8012 | 1.80E-75 | 3.93E-74 | 161.71 |
| 22405 | TGFB1 | 6.099961 | 9.548991 | 30.11317 | 6.21E-74 | 3.71E-73 | 158.1813 |
| 9966 | LIMS1 | 6.091533 | 9.55474 | 29.77712 | 3.57E-73 | 1.52E-72 | 156.4382 |
| 16059 | OAZ1 | 6.086692 | 9.510178 | 30.57981 | 5.59E-75 | 7.24E-74 | 160.5804 |
| 516 | AKAP13 | 6.082051 | 9.488775 | 31.27042 | 1.66E-76 | 1.87E-74 | 164.0858 |
| 19546 | S100A8 | 6.060976 | 9.458912 | 29.48011 | 1.69E-72 | 5.65E-72 | 154.8866 |
| 16552 | ORC4 | 6.047756 | 9.455933 | 29.9346 | 1.57E-73 | 7.76E-73 | 157.2566 |
| 3404 | CFLAR | 6.047703 | 9.454697 | 31.14722 | 3.09E-76 | 2.06E-74 | 163.4643 |
| 4292 | CTSB | 6.04682 | 9.47153 | 30.34628 | 1.86E-74 | 1.55E-73 | 159.3829 |
| 23109 | TPM4 | 6.0449 | 9.466666 | 30.10882 | 6.35E-74 | 3.77E-73 | 158.1588 |
| 21380 | SPARC | 6.025257 | 9.422485 | 28.52066 | 2.77E-70 | 5.47E-70 | 149.8039 |
| 12660 | MBNL1 | 6.023828 | 9.424315 | 29.52647 | 1.33E-72 | 4.59E-72 | 155.1295 |
| 8688 | IL17RA | 6.013398 | 9.398971 | 31.39224 | 8.95E-77 | 1.87E-74 | 164.6985 |
| 15273 | NAP1L1 | 6.00866 | 9.399083 | 29.0671 | 1.50E-71 | 3.80E-71 | 152.712 |
| 910 | APLP2 | 6.005354 | 9.383925 | 30.99281 | 6.77E-76 | 2.57E-74 | 162.6831 |
| 3069 | CD44 | 5.993286 | 9.348723 | 30.89041 | 1.14E-75 | 3.20E-74 | 162.1635 |
| 12694 | MCL1 | 5.988905 | 9.357296 | 31.12184 | 3.51E-76 | 2.14E-74 | 163.3361 |
| 19367 | RPS2 | 5.979155 | 9.358251 | 30.80113 | 1.80E-75 | 3.93E-74 | 161.7096 |
| 19273 | RPL15 | 5.962346 | 9.340355 | 30.54918 | 6.54E-75 | 7.88E-74 | 160.4237 |
| 5498 | EIF4G2 | 5.951632 | 9.307308 | 30.44528 | 1.12E-74 | 1.12E-73 | 159.8913 |
| 10749 | LOC100131257 | 5.926375 | 9.276783 | 29.55538 | 1.14E-72 | 4.03E-72 | 155.2808 |
| 12834 | METTL21A | 5.922554 | 9.272686 | 29.44237 | 2.06E-72 | 6.72E-72 | 154.6887 |
| 24619 | ZEB2 | 5.897752 | 9.22287 | 30.91991 | 9.81E-76 | 2.97E-74 | 162.3134 |
| 22969 | TNFRSF1B | 5.893997 | 9.229874 | 30.95636 | 8.15E-76 | 2.76E-74 | 162.4983 |
| 18970 | RHOA | 5.888425 | 9.208782 | 30.75599 | 2.27E-75 | 4.32E-74 | 161.4797 |
| 20062 | SH3BGRL3 | 5.887906 | 9.212159 | 29.73776 | 4.38E-73 | 1.80E-72 | 156.2331 |
| 8985 | ITM2B | 5.879974 | 9.216066 | 30.04766 | 8.72E-74 | 4.83E-73 | 157.8425 |
| 22445 | THBS1 | 5.877573 | 9.185315 | 29.13367 | 1.05E-71 | 2.78E-71 | 153.0638 |
| 1043 | ARHGDIB | 5.862607 | 9.162369 | 29.98164 | 1.23E-73 | 6.38E-73 | 157.5006 |
| 17129 | PF4 | 5.853782 | 9.166946 | 27.37124 | 1.44E-67 | 2.02E-67 | 143.5701 |
| 21302 | SORL1 | 5.850621 | 9.119964 | 31.48543 | 5.60E-77 | 1.87E-74 | 165.1662 |
| 1879 | C10orf54 | 5.835913 | 9.123092 | 31.03103 | 5.57E-76 | 2.46E-74 | 162.8767 |
| 8879 | IQGAP1 | 5.83416 | 9.105194 | 29.98782 | 1.19E-73 | 6.22E-73 | 157.5326 |
| 15175 | MYO1F | 5.82992 | 9.090644 | 31.46741 | 6.13E-77 | 1.87E-74 | 165.0759 |
| 6869 | FYB | 5.816729 | 9.083614 | 30.13773 | 5.47E-74 | 3.39E-73 | 158.3082 |
| 8962 | ITGB1 | 5.813907 | 9.093374 | 28.93327 | 3.06E-71 | 7.25E-71 | 152.0031 |
| 7581 | GPX1 | 5.806588 | 9.076964 | 30.29853 | 2.38E-74 | 1.85E-73 | 159.1373 |
| 3278 | CECR1 | 5.806268 | 9.076635 | 31.14763 | 3.08E-76 | 2.06E-74 | 163.4664 |
| 18625 | RAP1B | 5.804728 | 9.06626 | 28.47161 | 3.61E-70 | 6.94E-70 | 149.5412 |
| 4853 | DIAPH1 | 5.802305 | 9.064751 | 30.58026 | 5.57E-75 | 7.24E-74 | 160.5827 |
| 19321 | RPL4 | 5.792279 | 9.045254 | 30.82781 | 1.57E-75 | 3.65E-74 | 161.8453 |
| 19916 | SERPINA1 | 5.79033 | 9.046901 | 30.57378 | 5.76E-75 | 7.37E-74 | 160.5495 |
| 19593 | SAMHD1 | 5.783648 | 9.053946 | 30.66755 | 3.56E-75 | 5.59E-74 | 161.0287 |
| 7308 | GNAI2 | 5.772751 | 9.015386 | 31.46729 | 6.13E-77 | 1.87E-74 | 165.0752 |
| 17995 | PRKAR2B | 5.762046 | 9.01271 | 28.31187 | 8.53E-70 | 1.54E-69 | 148.6834 |
| 24315 | WIPF1 | 5.761773 | 9.004939 | 30.25572 | 2.97E-74 | 2.16E-73 | 158.9168 |
| 77 | ABCC9 | 5.757269 | 9.004462 | 29.0393 | 1.74E-71 | 4.33E-71 | 152.5649 |
| 19662 | SCARNA10 | 5.75521 | 9.003478 | 29.25067 | 5.68E-72 | 1.61E-71 | 153.681 |
| 5370 | EEF1G | 5.746312 | 8.976843 | 31.21349 | 2.21E-76 | 1.88E-74 | 163.7988 |
| 22928 | TMSB10 | 5.745203 | 8.976211 | 30.97539 | 7.40E-76 | 2.69E-74 | 162.5948 |
| 22947 | TNFAIP2 | 5.745111 | 8.971434 | 31.06254 | 4.75E-76 | 2.32E-74 | 163.0362 |
| 580 | ALDOA | 5.738506 | 8.974217 | 30.68065 | 3.33E-75 | 5.36E-74 | 161.0956 |
| 8246 | HNRNPU | 5.735379 | 8.974932 | 31.12127 | 3.52E-76 | 2.14E-74 | 163.3332 |
| 15356 | NCF2 | 5.734511 | 8.955246 | 30.75977 | 2.22E-75 | 4.30E-74 | 161.499 |
| 7652 | GRN | 5.7342 | 8.958357 | 30.80842 | 1.73E-75 | 3.89E-74 | 161.7467 |
| 4648 | DDX17 | 5.733982 | 8.948212 | 31.33556 | 1.19E-76 | 1.87E-74 | 164.4136 |
| 237 | ACTR2 | 5.731361 | 8.958915 | 29.98628 | 1.20E-73 | 6.26E-73 | 157.5246 |
| 8202 | HMHA1 | 5.726838 | 8.942759 | 30.92568 | 9.53E-76 | 2.94E-74 | 162.3426 |
| 20115 | SHISA9 | 5.722941 | 8.953884 | 28.87582 | 4.15E-71 | 9.57E-71 | 151.698 |
| 18307 | PTGS1 | 5.721029 | 8.941191 | 28.92589 | 3.18E-71 | 7.51E-71 | 151.9639 |
| 12547 | MAP3K7CL | 5.717688 | 8.972185 | 27.08065 | 7.17E-67 | 9.60E-67 | 141.9687 |
| 21585 | SRGN | 5.716448 | 8.941888 | 29.32859 | 3.76E-72 | 1.13E-71 | 154.0911 |
| 6522 | FGR | 5.712336 | 8.918615 | 31.00406 | 6.39E-76 | 2.57E-74 | 162.7401 |
| 15150 | MYL6 | 5.712033 | 8.934893 | 30.19156 | 4.14E-74 | 2.77E-73 | 158.586 |
| 2375 | C6orf25 | 5.7062 | 8.945595 | 29.04498 | 1.69E-71 | 4.22E-71 | 152.595 |
| 5085 | DOCK8 | 5.70452 | 8.891489 | 31.21946 | 2.14E-76 | 1.88E-74 | 163.8289 |
| 17535 | PLXNB2 | 5.698665 | 8.905046 | 30.58002 | 5.58E-75 | 7.24E-74 | 160.5815 |
| 810 | ANO6 | 5.693428 | 8.917026 | 29.73357 | 4.48E-73 | 1.83E-72 | 156.2113 |
| 8543 | IFI30 | 5.691567 | 8.896863 | 30.45137 | 1.08E-74 | 1.10E-73 | 159.9226 |
| 8155 | HLA-DRB1 | 5.688476 | 8.883982 | 30.86401 | 1.30E-75 | 3.38E-74 | 162.0294 |
| 4673 | DDX5 | 5.686605 | 8.869578 | 30.77576 | 2.05E-75 | 4.17E-74 | 161.5804 |
| 980 | ARAP1 | 5.68657 | 8.878165 | 30.84981 | 1.40E-75 | 3.47E-74 | 161.9572 |
| 19301 | RPL3 | 5.686211 | 8.886318 | 30.9302 | 9.31E-76 | 2.91E-74 | 162.3656 |
| 25341 | ZYX | 5.685271 | 8.893135 | 30.80055 | 1.80E-75 | 3.93E-74 | 161.7066 |
| 3719 | CLU | 5.683903 | 8.896013 | 28.1802 | 1.74E-69 | 3.00E-69 | 147.9741 |
| 8958 | ITGAL | 5.682504 | 8.864663 | 31.71435 | 1.77E-77 | 1.87E-74 | 166.3109 |
| 8227 | HNRNPA2B1 | 5.678608 | 8.867996 | 30.97173 | 7.54E-76 | 2.73E-74 | 162.5763 |
| 23784 | UGGT1 | 5.676855 | 8.907104 | 29.09013 | 1.33E-71 | 3.40E-71 | 152.8338 |
| 9383 | KIF2A | 5.67542 | 8.884938 | 27.72049 | 2.12E-68 | 3.21E-68 | 145.4812 |
| 9527 | KMT2C | 5.670915 | 8.853599 | 30.75933 | 2.23E-75 | 4.30E-74 | 161.4968 |
| 2608 | CAP1 | 5.66609 | 8.851302 | 29.92843 | 1.62E-73 | 7.93E-73 | 157.2246 |
| 22427 | TGOLN2 | 5.660994 | 8.841579 | 31.4714 | 6.01E-77 | 1.87E-74 | 165.0959 |
| 3401 | CFL1 | 5.650934 | 8.832479 | 30.75463 | 2.28E-75 | 4.34E-74 | 161.4728 |
| 8961 | ITGAX | 5.646768 | 8.821791 | 31.1864 | 2.53E-76 | 1.93E-74 | 163.6621 |
| 3100 | CD93 | 5.645445 | 8.83283 | 30.44927 | 1.09E-74 | 1.11E-73 | 159.9117 |
| 12309 | LSP1 | 5.645089 | 8.827364 | 30.68907 | 3.19E-75 | 5.22E-74 | 161.1385 |
| 4525 | DAPP1 | 5.639879 | 8.857835 | 29.28421 | 4.75E-72 | 1.38E-71 | 153.8576 |
| 21790 | STK4 | 5.637932 | 8.829374 | 30.71675 | 2.77E-75 | 4.85E-74 | 161.2797 |
| 19322 | RPL41 | 5.629292 | 8.813857 | 30.40103 | 1.40E-74 | 1.28E-73 | 159.6642 |
| 15144 | MYL12A | 5.627199 | 8.797649 | 28.70105 | 1.05E-70 | 2.23E-70 | 150.7678 |
| 12791 | MEGF9 | 5.624426 | 8.804327 | 30.81441 | 1.68E-75 | 3.81E-74 | 161.7772 |
| 24000 | UTRN | 5.61659 | 8.756782 | 30.73947 | 2.47E-75 | 4.56E-74 | 161.3955 |
| 17997 | PRKCB | 5.614937 | 8.77553 | 30.93205 | 9.22E-76 | 2.89E-74 | 162.375 |
| 19259 | RPL10 | 5.612963 | 8.767437 | 30.55548 | 6.33E-75 | 7.77E-74 | 160.4559 |
| 19382 | RPS3A | 5.612618 | 8.774205 | 30.10557 | 6.46E-74 | 3.82E-73 | 158.142 |
| 2571 | CALM3 | 5.60981 | 8.777398 | 29.76537 | 3.80E-73 | 1.60E-72 | 156.3769 |
| 8258 | HOOK3 | 5.606128 | 8.801719 | 29.63046 | 7.69E-73 | 2.90E-72 | 155.6733 |
| 12141 | LPP | 5.604179 | 8.787003 | 29.53548 | 1.27E-72 | 4.41E-72 | 155.1766 |
| 22085 | TAPBP | 5.598885 | 8.754435 | 30.51695 | 7.72E-75 | 8.78E-74 | 160.2587 |
| 12639 | MAX | 5.598802 | 8.753988 | 29.06825 | 1.49E-71 | 3.78E-71 | 152.7181 |
| 12270 | LRRFIP1 | 5.597407 | 8.756216 | 31.33644 | 1.19E-76 | 1.87E-74 | 164.418 |
| 7357 | GNS | 5.594656 | 8.752487 | 30.82258 | 1.61E-75 | 3.71E-74 | 161.8187 |
| 21293 | SON | 5.594034 | 8.738519 | 31.31109 | 1.35E-76 | 1.87E-74 | 164.2905 |
| 9290 | KIAA0513 | 5.593383 | 8.751231 | 30.88443 | 1.18E-75 | 3.23E-74 | 162.1331 |
| 2607 | CANX | 5.589521 | 8.735577 | 30.56574 | 6.01E-75 | 7.56E-74 | 160.5084 |
| 12783 | MEFV | 5.585218 | 8.747795 | 29.72177 | 4.77E-73 | 1.93E-72 | 156.1498 |
| 6698 | FOS | 5.584809 | 8.711795 | 30.29401 | 2.43E-74 | 1.88E-73 | 159.114 |
| 24214 | WDR1 | 5.582774 | 8.715654 | 29.90803 | 1.80E-73 | 8.62E-73 | 157.1187 |
| 4294 | CTSD | 5.578383 | 8.718455 | 30.92704 | 9.46E-76 | 2.94E-74 | 162.3495 |
| 18684 | RASSF2 | 5.576133 | 8.707168 | 31.21663 | 2.17E-76 | 1.88E-74 | 163.8147 |
| 3939 | CORO1A | 5.575043 | 8.694259 | 31.21903 | 2.15E-76 | 1.88E-74 | 163.8267 |
| 21493 | SPN | 5.574443 | 8.728457 | 29.76147 | 3.87E-73 | 1.62E-72 | 156.3566 |
| 5271 | DYNC1H1 | 5.572639 | 8.692811 | 31.43787 | 7.11E-77 | 1.87E-74 | 164.9277 |
| 531 | AKNA | 5.566643 | 8.689701 | 31.4224 | 7.69E-77 | 1.87E-74 | 164.85 |
| 20840 | SNN | 5.566024 | 8.706264 | 29.48692 | 1.63E-72 | 5.47E-72 | 154.9223 |
| 12730 | MDM4 | 5.560129 | 8.714885 | 28.84944 | 4.78E-71 | 1.09E-70 | 151.5579 |
| 22542 | TKT | 5.557862 | 8.668551 | 30.83767 | 1.49E-75 | 3.57E-74 | 161.8955 |
| 19332 | RPLP1 | 5.555601 | 8.707786 | 30.16205 | 4.82E-74 | 3.08E-73 | 158.4338 |
| 17433 | PLCB2 | 5.554611 | 8.672549 | 30.87537 | 1.23E-75 | 3.28E-74 | 162.0871 |
| 8947 | ITGA2B | 5.552133 | 8.678839 | 28.55016 | 2.37E-70 | 4.72E-70 | 149.9618 |
| 19278 | RPL19 | 5.54952 | 8.66859 | 30.8345 | 1.52E-75 | 3.60E-74 | 161.8794 |
| 14707 | MNDA | 5.548282 | 8.650561 | 29.23492 | 6.17E-72 | 1.74E-71 | 153.598 |
| 19371 | RPS24 | 5.546811 | 8.676711 | 30.71573 | 2.78E-75 | 4.85E-74 | 161.2745 |
| 21805 | STON2 | 5.545991 | 8.682014 | 29.54014 | 1.24E-72 | 4.32E-72 | 155.201 |
| 6196 | FAM65B | 5.538011 | 8.640799 | 31.7109 | 1.80E-77 | 1.87E-74 | 166.2938 |
| 6459 | FERMT3 | 5.534119 | 8.651041 | 30.45673 | 1.05E-74 | 1.08E-73 | 159.95 |
| 18324 | PTP4A2 | 5.529709 | 8.661251 | 29.93524 | 1.56E-73 | 7.74E-73 | 157.26 |
| 8530 | IDS | 5.527666 | 8.667524 | 29.35305 | 3.31E-72 | 1.01E-71 | 154.2197 |
| 23112 | TPP1 | 5.522689 | 8.641411 | 30.12416 | 5.86E-74 | 3.57E-73 | 158.2381 |
| 24104 | VPS13C | 5.522121 | 8.628451 | 30.2856 | 2.54E-74 | 1.93E-73 | 159.0707 |
| 12339 | LUZP6 | 5.520425 | 8.660242 | 29.28273 | 4.79E-72 | 1.39E-71 | 153.8499 |
| 15034 | MTPN | 5.520425 | 8.660242 | 29.28273 | 4.79E-72 | 1.39E-71 | 153.8499 |
| 5432 | EHBP1L1 | 5.516917 | 8.620361 | 30.63091 | 4.30E-75 | 6.25E-74 | 160.8416 |
| 20064 | SH3BP2 | 5.515767 | 8.613752 | 30.78949 | 1.91E-75 | 4.09E-74 | 161.6503 |
| 7596 | GRAP2 | 5.515526 | 8.6379 | 28.04246 | 3.67E-69 | 6.05E-69 | 147.2298 |
| 17529 | PLXDC2 | 5.509454 | 8.628369 | 30.67848 | 3.37E-75 | 5.40E-74 | 161.0845 |
| 19387 | RPS6 | 5.506414 | 8.602885 | 30.41401 | 1.31E-74 | 1.23E-73 | 159.7308 |
| 18337 | PTPN18 | 5.50625 | 8.616578 | 30.15327 | 5.04E-74 | 3.20E-73 | 158.3884 |
| 14286 | MIR6723 | 5.502843 | 8.592128 | 27.10819 | 6.15E-67 | 8.28E-67 | 142.121 |
| 16848 | PCBP2 | 5.500689 | 8.620506 | 30.15501 | 5.00E-74 | 3.18E-73 | 158.3974 |

**Supplementary Table2: Differentially expressed genes in each gene modules.**

| **Gene Modules** | **Genes** |
| --- | --- |
| **c1_2** | LUZP6, YWHAZ, ITGB1, PTGS1, FLNA, SPARC, GRAP2, TUBB1, VCL, CLU, PTPRJ, TAGLN2,ITGA2B, NAP1L1,ITGB3, TLN1, MAX, LINC00657, HLA-E, F13A1, PRKAR2B, NRGN, GNAS, ANO6, C6orf25, MBNL1, ACTB, SDPR, THBS1, SNN, WDR1, CALM3, TMSB4X, PKM, TPM4,WIPF1, OAZ1, RGS18, GPX1, KIF2A, ACTG1, B2M, EIF4G2, RAP1B, SH3BGRL3, FTH1, MYL12A,RHOA, ITM2B, DIAPH1,NCOA4, ARHGDIB, ALDOA, GAPDH, LIMS1, MYH9, CAP1, PPBP, FYB, MAP3K7CL, FTL, ZYX, HLA-B, TPP1, SRGN, ZEB2, MALAT1, KMT2C, HLA-C, HLA-A, HNRNPA2B1, TGFB1, RMRP, RPPH1, PTMA, RNF213, XIST, PLCG2, PF4, RNVU1-19, MTPN, PTPN18, FERMT3, STON2, MYL6, PTP4A2, CFL1, PRKCB, PCBP2, RPL41, UTRN, SCARNA10, RPLP1, LRRFIP1, MIR6723 |
| **c1_3** | KCNQ1OT1, UGDH-AS1, LOC101927374 CCL5, **UGGT1**, ORC4, ABCC9, LINC00506, MEFV, MDM4, HOOK3, TAPBP, SHISA9, CTSB, LOC100131257 METTL21A, STK4, RPL15, SPN, DAPP1, TPM3, HMHA1, ITGAX, KIAA0513, IDS, LPP, CFLAR, SH3BP2, GNS |
| **c1_4** | **MPEG1**, S100A9, LCP1, CYBB, EEF1A1, PTPRC, VCAN, **IQGAP1**, CD44, PLXNB2, NCF2, ARAP1, TXNIP, APLP2, LAPTM5, AKAP13, ACTR2, FGL2, RPL10, RPL4, RPS3A, CANX, NOTCH2, SERPINA1, SAMHD1, COTL1, GRN, VIM, PSAP, TPT1, PLEC, MEGF9, CD74, HLA-DRA, PABPC1, ITGB2, C10orf54, RPL19, SORL1, LYST, MSN, IFI30, DOCK8, EEF1G, EEF2, PLCB2, TNFAIP2, LYZ, AHNAK, DDX17, TMSB10, CTSS, CECR1, GNAI2, RPS24, DDX5, AKNA, MCL1, LRP1, FCN1, TNFRSF1B, MNDA, SRRM2, IL17RA, UBC, LSP1, MYO1F, RPS2, NEAT1, RPL3, SON, NBPF10, S100A8, FGR, HNRNPU, RASSF2, EHBP1L1, VPS13C, RPS6, CTSD, DYNC1H1, CD93, TGOLN2, PLXDC2, TKT, FAM65B, CORO1A, HLA-DRB1, ITGAL, FOS |
| **c1_5** | **LUZP6**, YWHAZ, ITGB1, PTPRJ, NAP1L1, LINC00657, PRKAR2B, **ANO6**, MBNL1, SDPR, TMSB4X, WIPF1, RGS18, KIF2A, B2M, RAP1B, FTH1, MYL12A, ITM2B, DIAPH1, NCOA4, LIMS1, PPBP, MAP3K7CL, PTMA, PF4, MTPN, STON2, PRKCB, MIR6723 |
| **c1_6** | **PTGS1**, FLNA, SPARC, GRAP2, TUBB1, VCL, CLU, TAGLN2, ITGA2B, ITGB3, TLN1, MAX, HLA-E, F13A1, NRGN, GNAS, C6orf25, THBS1, SNN, WDR1, CALM3, PKM, TPM4, OAZ1, GPX1, SH3BGRL3, MYH9, ZYX, TGFB1, RMRP, RPPH1, PTPN18, FERMT3, MYL6, SCARNA10 |
| **c1_8** | EIF4G2, RHOA, ARHGDIB, GAPDH, CAP1, FYB, TPP1, SRGN, KMT2C, PTP4A2, PCBP2, RPL41 |
| **c1_10** | KCNQ1OT1, UGDH-AS1, LOC101927374, ORC4, ABCC9, LINC00506, SHISA9, LOC100131257, LPP, CFLAR |
| **c1_12** | **MPEG1**, S100A9, LCP1, CYBB, EEF1A1, PTPRC, VCAN, **IQGAP1**, CD44, NCF2, TXNIP, APLP2, LAPTM5, AKAP13, ACTR2, FGL2, RPL10, RPL4, RPS3A, CANX, NOTCH2, SERPINA1, SAMHD1, COTL1, VIM, PSAP, TPT1, MEGF9, CD74, HLA-DRA, PABPC1, RPL19, SORL1, LYST, MSN, IFI30, DOCK8, EEF1G, EEF2, LYZ, AHNAK, DDX17, TMSB10, CTSS, CECR1, RPS24, DDX5, MCL1, MNDA, UBC, SON, S100A8, FGR, RASSF2, VPS13C, RPS6, DYNC1H1, CD93, TGOLN2, PLXDC2, TKT, FAM65B, HLA-DRB1, FOS |
| **c1_13** | PLXNB2, ARAP1, GRN, PLEC, ITGB2, C10orf54, PLCB2, TNFAIP2, GNAI2, AKNA, LRP1, FCN1, TNFRSF1B, SRRM2, IL17RA, LSP1, MYO1F, RPS2, NEAT1, RPL3, NBPF10, HNRNPU, EHBP1L1, CTSD, CORO1A, ITGAL |
| **c1_16** | ITGB1, PTPRJ, NAP1L1, LINC00657, PRKAR2B, **ANO6**, WIPF1, KIF2A, DIAPH1, LIMS1, STON2, PRKCB |
| **c1_18** | **PTGS1**, VCL, **CLU**, ITGB3, TLN1, MAX, HLA-E, F13A1, NRGN, C6orf25, THBS1, WDR1, CALM3, PKM, TPM4, GPX1, TGFB1, PTPN18 |
| **c1_23** | **MPEG1**, LCP1, CYBB, PTPRC, VCAN, **IQGAP1**, CD44, NCF2, TXNIP, APLP2, LAPTM5, AKAP13,ACTR2, FGL2, CANX, NOTCH2, SERPINA1, SAMHD1, COTL1, VIM, PSAP, MEGF9, CD74, HLA-DRA, PABPC1, SORL1, LYST, MSN, IFI30, DOCK8, EEF1G, EEF2, LYZ, AHNAK, DDX17, CTSS, CECR1, DDX5, MCL1, MNDA, SON, FGR, RASSF2, VPS13C, DYNC1H1, CD93, TGOLN2, PLXDC2, TKT, FAM65B, HLA-DRB1, FOS |
| **c1_24** | S100A9, EEF1A1, RPL10, RPL4, RPS3A, TPT1, RPL19, TMSB10, RPS24, UBC, S100A8, RPS6 |
| **c1_25** | PLXNB2, ARAP1, PLEC, **PLCB2**, TNFAIP2, AKNA, SRRM2, MYO1F, NEAT1, NBPF10, HNRNPU, EHBP1L1 |
| **c1_26** | GRN, ITGB2, C10orf54, GNAI2, LRP1, FCN1, TNFRSF1B, IL17RA, LSP1, RPS2, RPL3, CTSD, CORO1A, ITGAL |
| **c1_29** | **MPEG1**, LCP1, PTPRC, VCAN, IQGAP1, CD44, TXNIP, APLP2, LAPTM5, AKAP13, CANX, NOTCH2, SERPINA1, SAMHD1, PABPC1, DOCK8, AHNAK, CECR1, MCL1, SON, RASSF2, DYNC1H1, CD93, TGOLN2, FAM65B |
| **c1_34** | **MPEG1**, LCP1, PTPRC, VCAN, IQGAP1, CD44, TXNIP, APLP2, LAPTM5, CANX, NOTCH2, SERPINA1, SAMHD1, PABPC1, DOCK8, CECR1, MCL1, SON, RASSF2, CD93, TGOLN2 |

**Supplementary Table3: GO term enrichment analysis of DEGs.**

| **PANTHER Pathways** | **Gene counts** | **fold Enrichment** | **raw P-value** | **FDR** |
| --- | --- | --- | --- | --- |
| Glycolysis (P00024) | 3 | 15.07 | 1.44E-03 | 3.02E-02 |
| T cell activation (P00053) | 8 | 9.35 | 4.18E-06 | 1.75E-04 |
| Blood coagulation (P00011) | 4 | 8.37 | 1.71E-03 | 3.18E-02 |
| Integrin signalling pathway (P00034) | 15 | 7.81 | 2.44E-09 | 2.04E-07 |
| Cytoskeletal regulation by Rho GTPase (P00016) | 6 | 7.35 | 2.36E-04 | 7.88E-03 |
| B cell activation (P00010) | 5 | 7.18 | 8.74E-04 | 2.43E-02 |
| Inflammation mediated by chemokine and cytokine signaling pathway (P00031) | 16 | 6.3 | 1.25E-08 | 6.98E-07 |
| Huntington disease (P00029) | 7 | 4.75 | 8.87E-04 | 2.12E-02 |
| Alzheimer disease-presenilin pathway (P00004) | 6 | 4.75 | 2.07E-03 | 3.15E-02 |
| CCKR signaling map (P06959) | 7 | 4.09 | 2.03E-03 | 3.39E-02 |
| Gonadotropin-releasing hormone receptor pathway (P06664) | 8 | 3.48 | 2.63E-03 | 3.65E-02 |

**Supplementary Table4: Differentially methylated regions (both hyper- and hypo- methylated)**

| **chr** | **start** | **end** | **strand** | **pvalue** | **qvalue** | **meth.diff** |
| --- | --- | --- | --- | --- | --- | --- |
| 17 | 75284971 | 75284971 | + | 1.76E-126 | 5.21E-123 | 18.64026 |
| 6 | 163743051 | 163743051 | - | 2.32E-55 | 1.54E-52 | 17.65037 |
| 11 | 129594021 | 129594021 | - | 1.30E-103 | 2.50E-100 | 17.05906 |
| 19 | 54545186 | 54545186 | + | 8.37E-79 | 9.76E-76 | 17.03821 |
| 9 | 137131610 | 137131610 | + | 2.93E-138 | 1.04E-134 | 15.9169 |
| 6 | 110721154 | 110721154 | - | 4.40E-55 | 2.84E-52 | 15.36733 |
| 6 | 110721178 | 110721178 | - | 1.65E-48 | 7.75E-46 | 15.18883 |
| 6 | 110721139 | 110721139 | - | 5.45E-55 | 3.46E-52 | 15.10461 |
| 16 | 4846855 | 4846855 | - | 1.24E-137 | 4.21E-134 | 14.77192 |
| 6 | 110721167 | 110721167 | - | 3.79E-52 | 2.16E-49 | 14.68311 |
| 1 | 234849338 | 234849338 | + | 5.16E-75 | 5.48E-72 | 13.99867 |
| 4 | 29544573 | 29544573 | + | 1.35E-48 | 6.44E-46 | 13.72217 |
| 5 | 1393877 | 1393877 | + | 9.26E-63 | 8.23E-60 | 13.65519 |
| 14 | 99984589 | 99984589 | - | 4.75E-90 | 6.89E-87 | 13.40685 |
| X | 47213765 | 47213765 | + | 1.47E-62 | 1.29E-59 | 13.36779 |
| 12 | 239300 | 239300 | + | 2.62E-89 | 3.73E-86 | 13.12604 |
| 5 | 1159270 | 1159270 | - | 1.71E-61 | 1.41E-58 | 12.82923 |
| X | 153065323 | 153065323 | + | 1.25E-55 | 8.55E-53 | 12.49063 |
| 4 | 6946572 | 6946572 | - | 4.48E-39 | 1.37E-36 | 12.07124 |
| 4 | 3494847 | 3494847 | - | 5.61E-61 | 4.58E-58 | 11.98901 |
| 16 | 5550223 | 5550223 | - | 6.12E-237 | 7.25E-233 | 11.78926 |
| 9 | 124659921 | 124659921 | - | 1.84E-123 | 5.04E-120 | 11.78709 |
| 7 | 157452030 | 157452030 | + | 1.98E-49 | 9.59E-47 | 11.6971 |
| 19 | 55941195 | 55941195 | + | 8.83E-44 | 3.43E-41 | 11.6809 |
| 3 | 134635275 | 134635275 | + | 2.97E-26 | 4.41E-24 | 11.19476 |
| 22 | 45827019 | 45827019 | + | 2.25E-29 | 4.14E-27 | 11.17762 |
| 4 | 49151818 | 49151818 | - | 4.87E-165 | 2.04E-161 | 10.97988 |
| 14 | 65188392 | 65188392 | + | 6.11E-36 | 1.63E-33 | 10.84047 |
| 6 | 160776653 | 160776653 | - | 1.79E-27 | 2.90E-25 | 10.66714 |
| 9 | 136574473 | 136574473 | - | 6.65E-30 | 1.26E-27 | 10.6632 |
| 14 | 24540415 | 24540415 | + | 2.04E-39 | 6.38E-37 | 10.51126 |
| 7 | 1363621 | 1363621 | + | 2.85E-124 | 8.12E-121 | 10.36891 |
| 11 | 69878010 | 69878010 | - | 3.21E-30 | 6.21E-28 | 10.2566 |
| 16 | 5550218 | 5550218 | - | 3.51E-201 | 2.77E-197 | 10.22861 |
| 19 | 36755380 | 36755380 | + | 2.72E-46 | 1.18E-43 | 10.00286 |
| 8 | 145164576 | 145164576 | - | 3.00E-36 | 8.16E-34 | -10.01 |
| 8 | 1813939 | 1813939 | - | 2.49E-42 | 8.80E-40 | -10.06778 |
| 2 | 239047321 | 239047321 | - | 6.77E-23 | 7.46E-21 | -10.07942 |
| 12 | 2544723 | 2544723 | + | 1.03E-38 | 3.12E-36 | -10.13569 |
| 12 | 113515759 | 113515759 | - | 2.05E-19 | 1.69E-17 | -10.18097 |
| 14 | 105995918 | 105995918 | + | 1.63E-43 | 6.03E-41 | -10.20122 |
| 18 | 77128144 | 77128144 | + | 1.12E-50 | 5.84E-48 | -10.23334 |
| 7 | 158182809 | 158182809 | - | 6.66E-51 | 3.56E-48 | -10.43707 |
| 16 | 73092605 | 73092605 | - | 1.44E-23 | 1.70E-21 | -10.55078 |
| 21 | 20866202 | 20866202 | - | 1.26E-22 | 1.36E-20 | -10.61543 |
| 14 | 105995908 | 105995908 | + | 7.91E-48 | 3.63E-45 | -10.61946 |
| 16 | 89703519 | 89703519 | + | 7.74E-50 | 3.82E-47 | -10.62478 |
| 8 | 145164614 | 145164614 | - | 4.16E-41 | 1.42E-38 | -10.64538 |
| 22 | 50473162 | 50473162 | + | 3.08E-65 | 2.85E-62 | -10.65179 |
| 12 | 109996689 | 109996689 | - | 3.76E-59 | 2.88E-56 | -10.74116 |
| 3 | 11652035 | 11652035 | - | 2.13E-42 | 7.56E-40 | -10.74465 |
| 8 | 145164594 | 145164594 | - | 4.68E-43 | 1.69E-40 | -10.78944 |
| 13 | 113422473 | 113422473 | - | 8.56E-41 | 2.85E-38 | -10.9759 |
| 20 | 4142801 | 4142801 | - | 3.06E-24 | 3.83E-22 | -11.04188 |
| 1 | 200885635 | 200885635 | + | 3.91E-86 | 5.25E-83 | -11.1124 |
| 5 | 1637565 | 1637565 | + | 9.52E-101 | 1.78E-97 | -11.3454 |
| 8 | 145164584 | 145164584 | - | 1.62E-51 | 8.71E-49 | -11.49041 |
| 13 | 72440674 | 72440674 | - | 2.24E-22 | 2.37E-20 | -11.55259 |
| 8 | 145164582 | 145164582 | - | 7.69E-52 | 4.24E-49 | -11.56006 |
| 13 | 72440662 | 72440662 | - | 2.78E-22 | 2.92E-20 | -11.68631 |
| 13 | 72440665 | 72440665 | - | 4.31E-23 | 4.85E-21 | -11.84422 |
| 12 | 113515752 | 113515752 | - | 2.72E-25 | 3.75E-23 | -11.84544 |
| 8 | 145164588 | 145164588 | - | 2.19E-56 | 1.54E-53 | -12.10411 |
| 7 | 4873323 | 4873323 | + | 8.08E-65 | 7.37E-62 | -12.14837 |
| 11 | 68832847 | 68832847 | + | 5.90E-72 | 6.09E-69 | -12.26524 |
| 8 | 145164599 | 145164599 | - | 2.97E-57 | 2.18E-54 | -12.36942 |
| 16 | 81248716 | 81248716 | + | 2.83E-93 | 4.37E-90 | -12.41596 |
| 19 | 986657 | 986657 | + | 8.72E-166 | 3.87E-162 | -12.49447 |
| 12 | 113515756 | 113515756 | - | 2.60E-29 | 4.76E-27 | -12.66391 |
| 13 | 72440668 | 72440668 | - | 3.09E-26 | 4.55E-24 | -12.70314 |
| 12 | 113515740 | 113515740 | - | 3.77E-29 | 6.79E-27 | -12.74583 |
| 18 | 2406195 | 2406195 | - | 1.21E-69 | 1.21E-66 | -12.816 |
| 13 | 72440671 | 72440671 | - | 1.58E-27 | 2.58E-25 | -12.91575 |
| 2 | 130586386 | 130586386 | + | 1.63E-76 | 1.82E-73 | -12.92675 |
| 13 | 72440647 | 72440647 | - | 7.25E-27 | 1.13E-24 | -13.00017 |
| 16 | 88816894 | 88816894 | - | 7.73E-159 | 3.05E-155 | -13.05148 |
| 13 | 72440656 | 72440656 | - | 3.84E-28 | 6.58E-26 | -13.19583 |
| 13 | 72440644 | 72440644 | - | 5.90E-28 | 9.99E-26 | -13.28128 |
| 12 | 113515738 | 113515738 | - | 2.14E-30 | 4.19E-28 | -13.53696 |
| 21 | 47850405 | 47850405 | + | 5.73E-95 | 9.05E-92 | -13.59048 |
| 13 | 72440650 | 72440650 | - | 1.88E-29 | 3.49E-27 | -13.64659 |
| 1 | 242121539 | 242121539 | + | 8.41E-117 | 2.14E-113 | -13.67389 |
| 5 | 12795630 | 12795630 | - | 9.99E-127 | 3.09E-123 | -13.71346 |
| 12 | 113515735 | 113515735 | - | 1.43E-31 | 3.04E-29 | -13.87985 |
| 16 | 84903208 | 84903208 | + | 5.75E-87 | 7.86E-84 | -14.08248 |
| 9 | 139819984 | 139819984 | + | 9.06E-86 | 1.19E-82 | -14.13529 |
| 6 | 178169 | 178169 | + | 1.56E-104 | 3.18E-101 | -14.35323 |
| 10 | 134624505 | 134624505 | - | 7.14E-144 | 2.67E-140 | -14.67826 |
| 12 | 113515720 | 113515720 | - | 2.56E-36 | 6.99E-34 | -14.92885 |
| 8 | 28227281 | 28227281 | - | 2.27E-44 | 8.99E-42 | -15.2398 |
| 20 | 47132784 | 47132784 | - | 0 | 0 | -15.9148 |
| X | 86937439 | 86937439 | - | 8.41E-104 | 1.66E-100 | -16.45169 |
| 12 | 122264647 | 122264647 | + | 0 | 0 | -20.04055 |
| 5 | 355049 | 355049 | - | 1.31E-185 | 7.78E-182 | -21.24946 |
| X | 6608536 | 6608536 | + | 8.05E-205 | 7.16E-201 | -21.88445 |

**Supplementary Table5: Differentially hyper-methylated regions**

| **chr** | **start** | **end** | **strand** | **pvalue** | **qvalue** | **meth.diff** |
| --- | --- | --- | --- | --- | --- | --- |
| 1 | 2.01E+08 | 2.01E+08 | + | 3.91E-86 | 5.25E-83 | -11.1124 |
| 1 | 2.42E+08 | 2.42E+08 | + | 8.41E-117 | 2.14E-113 | -13.67389 |
| 10 | 1.35E+08 | 1.35E+08 | - | 7.14E-144 | 2.67E-140 | -14.67826 |
| 11 | 68832847 | 68832847 | + | 5.90E-72 | 6.09E-69 | -12.26524 |
| 12 | 2544723 | 2544723 | + | 1.03E-38 | 3.12E-36 | -10.13569 |
| 12 | 1.1E+08 | 1.1E+08 | - | 3.76E-59 | 2.88E-56 | -10.74116 |
| 12 | 1.14E+08 | 1.14E+08 | - | 2.56E-36 | 6.99E-34 | -14.92885 |
| 12 | 1.14E+08 | 1.14E+08 | - | 1.43E-31 | 3.04E-29 | -13.87985 |
| 12 | 1.14E+08 | 1.14E+08 | - | 2.14E-30 | 4.19E-28 | -13.53696 |
| 12 | 1.14E+08 | 1.14E+08 | - | 3.77E-29 | 6.79E-27 | -12.74583 |
| 12 | 1.14E+08 | 1.14E+08 | - | 2.72E-25 | 3.75E-23 | -11.84544 |
| 12 | 1.14E+08 | 1.14E+08 | - | 2.60E-29 | 4.76E-27 | -12.66391 |
| 12 | 1.14E+08 | 1.14E+08 | - | 2.05E-19 | 1.69E-17 | -10.18097 |
| 12 | 1.22E+08 | 1.22E+08 | + | 0 | 0 | -20.04055 |
| 13 | 72440644 | 72440644 | - | 5.90E-28 | 9.99E-26 | -13.28128 |
| 13 | 72440647 | 72440647 | - | 7.25E-27 | 1.13E-24 | -13.00017 |
| 13 | 72440650 | 72440650 | - | 1.88E-29 | 3.49E-27 | -13.64659 |
| 13 | 72440656 | 72440656 | - | 3.84E-28 | 6.58E-26 | -13.19583 |
| 13 | 72440662 | 72440662 | - | 2.78E-22 | 2.92E-20 | -11.68631 |
| 13 | 72440665 | 72440665 | - | 4.31E-23 | 4.85E-21 | -11.84422 |
| 13 | 72440668 | 72440668 | - | 3.09E-26 | 4.55E-24 | -12.70314 |
| 13 | 72440671 | 72440671 | - | 1.58E-27 | 2.58E-25 | -12.91575 |
| 13 | 72440674 | 72440674 | - | 2.24E-22 | 2.37E-20 | -11.55259 |
| 13 | 1.13E+08 | 1.13E+08 | - | 8.56E-41 | 2.85E-38 | -10.9759 |
| 14 | 1.06E+08 | 1.06E+08 | + | 7.91E-48 | 3.63E-45 | -10.61946 |
| 14 | 1.06E+08 | 1.06E+08 | + | 1.63E-43 | 6.03E-41 | -10.20122 |
| 16 | 73092605 | 73092605 | - | 1.44E-23 | 1.70E-21 | -10.55078 |
| 16 | 81248716 | 81248716 | + | 2.83E-93 | 4.37E-90 | -12.41596 |
| 16 | 84903208 | 84903208 | + | 5.75E-87 | 7.86E-84 | -14.08248 |
| 16 | 88816894 | 88816894 | - | 7.73E-159 | 3.05E-155 | -13.05148 |
| 16 | 89703519 | 89703519 | + | 7.74E-50 | 3.82E-47 | -10.62478 |
| 18 | 2406195 | 2406195 | - | 1.21E-69 | 1.21E-66 | -12.816 |
| 18 | 77128144 | 77128144 | + | 1.12E-50 | 5.84E-48 | -10.23334 |
| 19 | 986657 | 986657 | + | 8.72E-166 | 3.87E-162 | -12.49447 |
| 2 | 1.31E+08 | 1.31E+08 | + | 1.63E-76 | 1.82E-73 | -12.92675 |
| 2 | 2.39E+08 | 2.39E+08 | - | 6.77E-23 | 7.46E-21 | -10.07942 |
| 20 | 4142801 | 4142801 | - | 3.06E-24 | 3.83E-22 | -11.04188 |
| 20 | 47132784 | 47132784 | - | 0 | 0 | -15.9148 |
| 21 | 20866202 | 20866202 | - | 1.26E-22 | 1.36E-20 | -10.61543 |
| 21 | 47850405 | 47850405 | + | 5.73E-95 | 9.05E-92 | -13.59048 |
| 22 | 50473162 | 50473162 | + | 3.08E-65 | 2.85E-62 | -10.65179 |
| 3 | 11652035 | 11652035 | - | 2.13E-42 | 7.56E-40 | -10.74465 |
| 5 | 355049 | 355049 | - | 1.31E-185 | 7.78E-182 | -21.24946 |
| 5 | 1637565 | 1637565 | + | 9.52E-101 | 1.78E-97 | -11.3454 |
| 5 | 12795630 | 12795630 | - | 9.99E-127 | 3.09E-123 | -13.71346 |
| 6 | 178169 | 178169 | + | 1.56E-104 | 3.18E-101 | -14.35323 |
| 7 | 4873323 | 4873323 | + | 8.08E-65 | 7.37E-62 | -12.14837 |
| 7 | 1.58E+08 | 1.58E+08 | - | 6.66E-51 | 3.56E-48 | -10.43707 |
| 8 | 1813939 | 1813939 | - | 2.49E-42 | 8.80E-40 | -10.06778 |
| 8 | 28227281 | 28227281 | - | 2.27E-44 | 8.99E-42 | -15.2398 |
| 8 | 1.45E+08 | 1.45E+08 | - | 3.00E-36 | 8.16E-34 | -10.01 |
| 8 | 1.45E+08 | 1.45E+08 | - | 7.69E-52 | 4.24E-49 | -11.56006 |
| 8 | 1.45E+08 | 1.45E+08 | - | 1.62E-51 | 8.71E-49 | -11.49041 |
| 8 | 1.45E+08 | 1.45E+08 | - | 2.19E-56 | 1.54E-53 | -12.10411 |
| 8 | 1.45E+08 | 1.45E+08 | - | 4.68E-43 | 1.69E-40 | -10.78944 |
| 8 | 1.45E+08 | 1.45E+08 | - | 2.97E-57 | 2.18E-54 | -12.36942 |
| 8 | 1.45E+08 | 1.45E+08 | - | 4.16E-41 | 1.42E-38 | -10.64538 |
| 9 | 1.4E+08 | 1.4E+08 | + | 9.06E-86 | 1.19E-82 | -14.13529 |
| X | 6608536 | 6608536 | + | 8.05E-205 | 7.16E-201 | -21.88445 |
| X | 86937439 | 86937439 | - | 8.41E-104 | 1.66E-100 | -16.45169 |

**Supplementary Table6: Differentially hypo-methylated regions**

| **chr** | **start** | **end** | **strand** | **pvalue** | **qvalue** | **meth.diff** |
| --- | --- | --- | --- | --- | --- | --- |
| 1 | 234849338 | 234849338 | + | 5.16E-75 | 5.48E-72 | 13.99867 |
| 11 | 69878010 | 69878010 | - | 3.21E-30 | 6.21E-28 | 10.2566 |
| 11 | 129594021 | 129594021 | - | 1.30E-103 | 2.50E-100 | 17.05906 |
| 12 | 239300 | 239300 | + | 2.62E-89 | 3.73E-86 | 13.12604 |
| 14 | 24540415 | 24540415 | + | 2.04E-39 | 6.38E-37 | 10.51126 |
| 14 | 65188392 | 65188392 | + | 6.11E-36 | 1.63E-33 | 10.84047 |
| 14 | 99984589 | 99984589 | - | 4.75E-90 | 6.89E-87 | 13.40685 |
| 16 | 4846855 | 4846855 | - | 1.24E-137 | 4.21E-134 | 14.77192 |
| 16 | 5550218 | 5550218 | - | 3.51E-201 | 2.77E-197 | 10.22861 |
| 16 | 5550223 | 5550223 | - | 6.12E-237 | 7.25E-233 | 11.78926 |
| 17 | 75284971 | 75284971 | + | 1.76E-126 | 5.21E-123 | 18.64026 |
| 19 | 36755380 | 36755380 | + | 2.72E-46 | 1.18E-43 | 10.00286 |
| 19 | 54545186 | 54545186 | + | 8.37E-79 | 9.76E-76 | 17.03821 |
| 19 | 55941195 | 55941195 | + | 8.83E-44 | 3.43E-41 | 11.6809 |
| 22 | 45827019 | 45827019 | + | 2.25E-29 | 4.14E-27 | 11.17762 |
| 3 | 134635275 | 134635275 | + | 2.97E-26 | 4.41E-24 | 11.19476 |
| 4 | 3494847 | 3494847 | - | 5.61E-61 | 4.58E-58 | 11.98901 |
| 4 | 6946572 | 6946572 | - | 4.48E-39 | 1.37E-36 | 12.07124 |
| 4 | 29544573 | 29544573 | + | 1.35E-48 | 6.44E-46 | 13.72217 |
| 4 | 49151818 | 49151818 | - | 4.87E-165 | 2.04E-161 | 10.97988 |
| 5 | 1159270 | 1159270 | - | 1.71E-61 | 1.41E-58 | 12.82923 |
| 5 | 1393877 | 1393877 | + | 9.26E-63 | 8.23E-60 | 13.65519 |
| 6 | 110721139 | 110721139 | - | 5.45E-55 | 3.46E-52 | 15.10461 |
| 6 | 110721154 | 110721154 | - | 4.40E-55 | 2.84E-52 | 15.36733 |
| 6 | 110721167 | 110721167 | - | 3.79E-52 | 2.16E-49 | 14.68311 |
| 6 | 110721178 | 110721178 | - | 1.65E-48 | 7.75E-46 | 15.18883 |
| 6 | 160776653 | 160776653 | - | 1.79E-27 | 2.90E-25 | 10.66714 |
| 6 | 163743051 | 163743051 | - | 2.32E-55 | 1.54E-52 | 17.65037 |
| 7 | 1363621 | 1363621 | + | 2.85E-124 | 8.12E-121 | 10.36891 |
| 7 | 157452030 | 157452030 | + | 1.98E-49 | 9.59E-47 | 11.6971 |
| 9 | 124659921 | 124659921 | - | 1.84E-123 | 5.04E-120 | 11.78709 |
| 9 | 136574473 | 136574473 | - | 6.65E-30 | 1.26E-27 | 10.6632 |
| 9 | 137131610 | 137131610 | + | 2.93E-138 | 1.04E-134 | 15.9169 |
| X | 47213765 | 47213765 | + | 1.47E-62 | 1.29E-59 | 13.36779 |
| X | 153065323 | 153065323 | + | 1.25E-55 | 8.55E-53 | 12.49063 |

**Supplementary Table7: Differentially methylated regions and their nearest genes**

| **feature.name** | **Gene** | **target.row** | **dist.to.feature** | **feature.strand** | **bin** | **chrom** | **strand** | **txStart** | **txEnd** | **cdsStart** | **cdsEnd** | **exonCount** |
| --- | --- | --- | --- | --- | --- | --- | --- | --- | --- | --- | --- | --- |
| NM_001142569 | INAVA | 1 | 21688 | + | 2117 | chr1 | + | 200863948 | 200884864 | 200867528 | 200882757 | 10 |
| NR_038856 | LINC01132 | 2 | -10451 | + | 2376 | chr1 | + | 234859788 | 234867390 | 234867390 | 234867390 | 3 |
| NM_001290693 | BECN2 | 3 | 472 | + | 2432 | chr1 | + | 242121068 | 242122364 | 242121068 | 242122364 | 1 |
| NM_177400 | NKX6-2 | 4 | -24969 | - | 1611 | chr10 | - | 134598319 | 134599537 | 134598419 | 134599452 | 3 |
| NM_139075 | TPCN2 | 5 | 16499 | + | 1110 | chr11 | + | 68816349 | 68858072 | 68816465 | 68855421 | 25 |
| NR_120530 | LOC101928443 | 6 | 32021 | - | 1118 | chr11 | - | 69902335 | 69910030 | 69910030 | 69910030 | 2 |
| NM_138788 | TMEM45B | 7 | -91720 | + | 1574 | chr11 | + | 129685675 | 129729406 | 129722377 | 129728580 | 6 |
| NR_033859 | LOC574538 | 8 | 19033 | - | 586 | chr12 | - | 246576 | 258335 | 258335 | 258335 | 10 |
| NR_046769 | CACNA1C-IT3 | 9 | 165783 | + | 603 | chr12 | + | 2378941 | 2397911 | 2397911 | 2397911 | 3 |
| NM_052845 | MMAB | 10 | 14670 | - | 1424 | chr12 | - | 109991520 | 110011358 | 109994832 | 110011285 | 9 |
| NM_004416 | DTX1 | 11 | 20060 | + | 181 | chr12 | + | 113495661 | 113535833 | 113495997 | 113534744 | 9 |
| NM_004392 | DACH1 | 24 | 666 | - | 17 | chr13 | - | 72012097 | 72441330 | 72014786 | 72440907 | 8 |
| NR_046661 | ATP11A-AS1 | 28 | -13429 | - | 1450 | chr13 | - | 113399762 | 113409045 | 113409045 | 113409045 | 2 |
| NM_006032 | CPNE6 | 29 | -141 | + | 772 | chr14 | + | 24540555 | 24547309 | 24542145 | 24546939 | 17 |
| NM_001099402 | CCNK | 31 | 36852 | + | 1347 | chr14 | + | 99947738 | 99977852 | 99959014 | 99977119 | 11 |
| NM_025268 | TMEM121 | 32 | 2957 | + | 1393 | chr14 | + | 105992939 | 105996539 | 105995171 | 105996131 | 2 |
| NM_001253794 | SMIM22 | 34 | 1478 | + | 621 | chr16 | + | 4845378 | 4846492 | 4845735 | 4846232 | 4 |
| NR_110902 | LINC01570 | 36 | 116029 | - | 628 | chr16 | - | 5651169 | 5666251 | 5666251 | 5666251 | 3 |
| NM_001164766 | ZFHX3 | 37 | -72 | - | 142 | chr16 | - | 72816785 | 73092534 | 72821062 | 72984841 | 9 |
| NM_052892 | PKD1L2 | 38 | 5260 | - | 1204 | chr16 | - | 81134481 | 81253999 | 81134727 | 81253975 | 44 |
| NM_031476 | CRISPLD2 | 39 | 49623 | + | 19 | chr16 | + | 84853586 | 84943116 | 84872101 | 84940248 | 15 |
| NR_120387 | LOC339059 | 40 | 7721 | + | 1262 | chr16 | + | 88809174 | 88812156 | 88812156 | 88812156 | 3 |
| NM_004413 | DPEP1 | 41 | 16521 | + | 1269 | chr16 | + | 89686999 | 89704839 | 89696818 | 89704630 | 11 |
| NM_001113492 | SEPT9 | 42 | 1000 | + | 144 | chr17 | + | 75283972 | 75496678 | 75398556 | 75494740 | 12 |
| NM_022840 | METTL4 | 43 | 165295 | - | 604 | chr18 | - | 2537523 | 2571502 | 2538998 | 2567215 | 9 |
| NM_006162 | NFATC1 | 44 | -27628 | + | 146 | chr18 | + | 77155771 | 77289323 | 77156224 | 77287530 | 10 |
| NM_024100 | WDR18 | 45 | 2331 | + | 592 | chr19 | + | 984327 | 994569 | 984353 | 994343 | 10 |
| NR_029389 | LOC100134317 | 46 | -46865 | + | 865 | chr19 | + | 36802244 | 36803570 | 36803570 | 36803570 | 2 |
| NM_001288793 | VSTM1 | 47 | 22022 | - | 1001 | chr19 | - | 54544079 | 54567207 | 54544214 | 54567031 | 6 |
| NM_001145176 | SHISA7 | 48 | 13036 | - | 1011 | chr19 | - | 55940104 | 55954230 | 55944522 | 55954230 | 4 |
| NR_110285 | LINC01856 | 49 | -94364 | + | 1582 | chr2 | + | 130680749 | 130704276 | 130704276 | 130704276 | 4 |
| NM_198582 | KLHL30 | 50 | -42 | + | 2408 | chr2 | + | 239047362 | 239061547 | 239049395 | 239059706 | 8 |
| NM_001270691 | SMOX | 51 | 13377 | + | 616 | chr20 | + | 4129425 | 4168394 | 4155702 | 4168054 | 8 |
| NR_026958 | LINC00494 | 52 | 144132 | + | 943 | chr20 | + | 46988653 | 46999381 | 46999381 | 46999381 | 5 |
| NR_109925 | MIR548XHG | 53 | -734073 | - | 92 | chr21 | - | 19933582 | 20132130 | 20132130 | 20132130 | 4 |
| NM_015151 | DIP2A | 54 | -28457 | + | 118 | chr21 | + | 47878861 | 47989926 | 47879044 | 47987535 | 38 |
| NM_015653 | RIBC2 | 55 | 17449 | + | 934 | chr22 | + | 45809571 | 45828302 | 45809767 | 45828242 | 8 |
| NM_001001694 | IL17REL | 56 | -22108 | - | 969 | chr22 | - | 50432941 | 50451055 | 50435466 | 50439619 | 15 |
| NM_001284391 | VGLL4 | 57 | -5981 | - | 673 | chr3 | - | 11597540 | 11646055 | 11600029 | 11643383 | 5 |
| NM_004441 | EPHB1 | 58 | 121178 | + | 201 | chr3 | + | 134514098 | 134979307 | 134514473 | 134977962 | 16 |
| NM_001256896 | DOK7 | 59 | 8578 | + | 611 | chr4 | + | 3486270 | 3496209 | 3494643 | 3495228 | 4 |
| NM_020773 | TBC1D14 | 60 | 35079 | + | 79 | chr4 | + | 6911494 | 7034845 | 6925116 | 7032119 | 14 |
| NR_036237 | MIR4275 | 61 | 723371 | + | 804 | chr4 | + | 28821203 | 28821290 | 28821290 | 28821290 | 1 |
| NM_001286791 | CWH43 | 62 | 163161 | + | 119 | chr4 | + | 48988658 | 49064095 | 48990531 | 49063907 | 16 |
| NM_020731 | AHRR | 63 | 50760 | + | 73 | chr5 | + | 304290 | 438405 | 304334 | 434949 | 12 |
| NR_109911 | CTD-3080P12.3 | 64 | 19451 | - | 593 | chr5 | - | 1173210 | 1178720 | 1178720 | 1178720 | 5 |
| NR_125810 | LINC01511 | 65 | -13690 | - | 595 | chr5 | - | 1363696 | 1380188 | 1380188 | 1380188 | 2 |
| NR_003713 | LOC728613 | 66 | -3446 | - | 597 | chr5 | - | 1597671 | 1634120 | 1634120 | 1634120 | 4 |
| NR_033383 | LINC01194 | 67 | 220663 | + | 10 | chr5 | + | 12574968 | 12805295 | 12805295 | 12805295 | 4 |
| NR_126020 | LOC285766 | 68 | 27316 | - | 586 | chr6 | - | 181465 | 205484 | 205484 | 205484 | 8 |
| NM_003649 | DDO | 70 | 15600 | - | 1429 | chr6 | - | 110713382 | 110736753 | 110713977 | 110736749 | 5 |
| NM_021977 | SLC22A3 | 73 | 7250 | + | 226 | chr6 | + | 160769404 | 160873611 | 160769451 | 160872088 | 11 |
| NR_028390 | PACRG-AS1 | 74 | 2455 | - | 1834 | chr6 | - | 163731016 | 163745505 | 163745505 | 163745505 | 6 |
| NM_001080461 | UNCX | 75 | 90969 | + | 594 | chr7 | + | 1272017 | 1276962 | 1272653 | 1276613 | 3 |
| NM_020144 | PAPOLB | 76 | 28303 | - | 622 | chr7 | - | 4897368 | 4901625 | 4899527 | 4901441 | 1 |
| NR_029689 | MIR153-2 | 77 | -84917 | - | 1785 | chr7 | - | 157367027 | 157367114 | 157367114 | 157367114 | 1 |
| NR_030325 | MIR595 | 78 | 142697 | - | 1792 | chr7 | - | 158325409 | 158325505 | 158325505 | 158325505 | 1 |
| NM_014629 | ARHGEF10 | 79 | 41792 | + | 74 | chr8 | + | 1772141 | 1906807 | 1791565 | 1905429 | 29 |
| NM_018660 | ZNF395 | 80 | 16697 | - | 800 | chr8 | - | 28203101 | 28243977 | 28206235 | 28218641 | 10 |
| NM_138709 | DAB2IP | 88 | 154859 | + | 191 | chr9 | + | 124505063 | 124545154 | 124519301 | 124544777 | 14 |
| NM_001134707 | SARDH | 89 | 29007 | - | 203 | chr9 | - | 136528683 | 136603479 | 136529010 | 136599295 | 21 |
| NM_002957 | RXRA | 90 | -86699 | + | 203 | chr9 | + | 137218308 | 137332432 | 137218477 | 137328460 | 10 |
| NM_018998 | FBXW5 | 91 | 19223 | - | 1651 | chr9 | - | 139834884 | 139839206 | 139835379 | 139838535 | 9 |
| NM_016379 | VCX3A | 92 | -155378 | - | 634 | chrX | - | 6451658 | 6453159 | 6451785 | 6452538 | 3 |
| NM_003446 | ZNF157 | 93 | -16234 | + | 945 | chrX | + | 47229998 | 47273098 | 47230067 | 47272993 | 4 |
| NM_019117 | KLHL4 | 94 | 164726 | + | 155 | chrX | + | 86772714 | 86925050 | 86772896 | 86921534 | 11 |
| NM_006280 | SSR4 | 95 | 5306 | + | 1752 | chrX | + | 153060018 | 153063967 | 153060142 | 153063888 | 6 |

**Supplementary Table8: DAVID enrichment analysis of the genes DAMs annoted to.**

| **Annotation** | **Category** | **Counts** | P-value | **FDR correction** |
| --- | --- | --- | --- | --- |
| Cytoplasm | UP_KEYWORDS | 5 | 0.09 | 0.98 |
| GO:0005737~cytoplasm | GOTERM_CC_DIRECT | 5 | 0.17 | 0.97 |
| GO:0006351~transcription, DNA-templated | GOTERM_BP_DIRECT | 3 | 0.19 | 1.00 |
| GO:0005634~nucleus | GOTERM_CC_DIRECT | 4 | 0.44 | 1.00 |
| Nucleus | UP_KEYWORDS | 3 | 0.64 | 1.00 |
| splice variant | UP_SEQ_FEATURE | 5 | 0.38 | 1.00 |
| Alternative splicing | UP_KEYWORDS | 5 | 0.67 | 1.00 |
| Polymorphism | UP_KEYWORDS | 5 | 0.80 | 1.00 |
| sequence variant | UP_SEQ_FEATURE | 5 | 0.86 | 1.00 |

**Supplementary Table9: Enrichment analysis of the metabolites.**

| **Annotation** | **Category** | **Counts** | | ***P*-value** | **FDR correction** |
| --- | --- | --- | --- | --- | --- |
| Amino sugar and nucleotide sugar metabolism (map00520) | KEGG pathways | 4 | 8.19E-06 | | 3.03E-04 |
| Amino Sugar Metabolism (SMP00045) | SMPDB Pathways (HMDB) | 3 | 5.96E-04 | | 1.13E-02 |
| Two-component system (map02020) | KEGG pathways | 2 | 1.07E-03 | | 1.98E-02 |
| Starch and sucrose metabolism (map00500) | KEGG pathways | 2 | 3.17E-03 | | 2.93E-02 |
| Phosphotransferase system (PTS) (map02060) | KEGG pathways | 2 | 2.69E-03 | | 2.93E-02 |
| Meiosis – yeast (map04113) | KEGG pathways | 1 | 5.26E-03 | | 3.58E-02 |
| Biosynthesis of plant hormones (map01070) | KEGG pathways | 2 | 5.80E-03 | | 3.58E-02 |
| Insulin signaling pathway (map04910) | KEGG pathways | 1 | 7.01E-03 | | 3.71E-02 |
| ABC transporters (map02010) | KEGG pathways | 2 | 1.00E-02 | | 3.89E-02 |
| Type II diabetes mellitus (map04930) | KEGG pathways | 1 | 1.05E-02 | | 3.89E-02 |
| Bacterial chemotaxis (map02030) | KEGG pathways | 1 | 1.05E-02 | | 3.89E-02 |
| Metabolic pathway (map01100) | KEGG pathways | 6 | 1.56E-02 | | 5.25E-02 |

**Supplementary Table10: Causal pairs between hub genes and methylation.**

| **Causal pairs** | **Method** | **Estimate** | **Standard Error** | **95% CI** | **P-value** |
| --- | --- | --- | --- | --- | --- |
| **Causality relationship: Genes → DMRs** |  |  |  |  |  |
| **ANO6_6.110721178** | Simple median | 2.545 | 1.198 | (0.197, 4.894) | 0.034 |
|  | Weighted median | 2.478 | 1.139 | (0.245, 4.711) | 0.03 |
|  | Inverse variance weighted | 2.324 | 0.691 | (0.691, 3.957) | **0.005** |
|  | MR-Egger Intercept | -36.103 | 116.977 | (-265.373, 193.167) | 0.758 |
|  | PRESSO Global test |  |  |  | 1 |
| 6.110721178_ANO6 | Simple median | -0.894 | 1.965 | (-4.74, 2.956) | 0.649 |
|  | Weighted median | -0.702 | 1.886 | (-4.398, 2.993) | 0.71 |
|  | Inverse variance weighted | 0.282 | 2.182 | (-3.996, 4.559) | 0.897 |
|  |  |  |  |  |  |
| **CLU_ 9.13713161** | Inverse variance weighted | 0.01 | 0.003 | (0.004, 0.016) | **0.002** |
|  | PRESSO MR | 9.02 | 3.737 | (1.695, 16.344) | 0.03 |
|  | MR-Egger Intercept | 3.24 | 31.818 | (-59.122, 65.602) | 0.919 |
|  | PRESSO Global test |  |  |  | 1 |
| 9.13713161_ CLU | Simple median | -0.106 | 3.776 | (-7.506, 7.294) | 0.978 |
|  | Weighted median | -0.889 | 3.145 | (-7.053, 5.274) | 0.778 |
|  | Inverse variance weighted | -0.056 | 2.519 | (-4.994, 4.881) | 0.982 |
|  |  |  |  |  |  |
| **MPEG1_6.163743051** | Simple median | -0.013 | 0.006 | (-0.025, -0.002) | 0.02 |
|  | Weighted median | -0.013 | 0.006 | (-0.025, -0.002) | 0.02 |
|  | Inverse variance weighted | -0.013 | 0.005 | (-0.023, -0.004) | **0.005** |
|  | PRESSO MR | -0.013 | 5.31E-05 | (-0.013, -0.012) | **1.89E-11** |
|  | MR-Egger Intercept | -9.918 | 410.5 | (-814.482, 794.646) | 0.981 |
|  | PRESSO Global test |  |  |  | 1 |
| 6.163743051_ MPEG1 | Simple median | 0.561 | 2.107 | (-3.569, 4.691) | 0.79 |
|  | Weighted median | -0.329 | 1.961 | (-4.173, 3.514) | 0.867 |
|  | Inverse variance weighted | 0.657 | 1.541 | (-2.364, 3.678) | 0.67 |
|  |  |  |  |  |  |
| **UGGT1_6.110721154** | Simple median | -0.004 | 0.002 | (-0.007, -0.0003) | 0.033 |
|  | Weighted median | -0.004 | 0.002 | (-0.007, -0.0003) | 0.033 |
|  | MR-Egger Intercept | -66.087 | 40.454 | (-145.375, 13.200) | 0.102 |
|  | PRESSO Global test |  |  |  | 1 |
| 6.110721154_UGGT1 | Simple median | 3.643 | 7.384 | (-10.830, 18.115) | 0.622 |
|  | Weighted median | 3.977 | 7.136 | (-10.010, 17.963) | 0.577 |
|  | Inverse variance weighted | 2.6 | 5.128 | (-7.452, 12.651) | 0.612 |
|  |  |  |  |  |  |
| **UGGT1_6.110721178** | Simple median | -0.004 | 0.001 | (-0.007, -0.0009) | **0.009** |
|  | Weighted median | -0.004 | 0.001 | (-0.007, -0.0008) | 0.012 |
|  | MR-Egger Intercept | -48.273 | 43.712 | (-133.948, 37.402) | 0.269 |
|  | PRESSO Global test |  |  |  | 1 |
| 6.110721178_ UGGT1 | Simple median | 2.889 | 6.829 | (-10.496, 16.274) | 0.672 |
|  | Weighted median | 6.924 | 6.402 | (-5.623, 19.472) | 0.279 |
|  | Inverse variance weighted | 6.002 | 4.58 | (-2.975, 14.979) | 0.19 |
| **Causality relationship: DMRs → Genes** |  |  |  |  |  |
| **6.163743051_ANO6** | Simple median | 2.545 | 1.198 | (0.197, 4.894) | 0.034 |
|  | Weighted median | 2.478 | 1.139 | (0.245, 4.711) | 0.03 |
|  | Inverse variance weighted | 2.324 | 0.691 | (0.691, 3.957) | **0.005** |
|  | MR-Egger Intercept | -36.103 | 116.977 | (-265.373, 193.167) | 0.758 |
|  | PRESSO Global test |  |  |  | 1 |
| **6.110721139_PTGS1** | Weighted median | 6.333 | 3.082 | (0.293, 12.373) | 0.04 |
|  | Inverse variance weighted | 6.719 | 2.505 | (1.809, 11.629) | **0.007** |
|  | PRESSO MR | 8.958 | 2.296 | (4.458, 13.458) | **0.002** |
|  | MR-Egger Intercept | -192.619 | 170.02 | (-525.851, 140.613) | 0.257 |
| PTGS1_6.110721139 | Simple median | -2.553 | 3.386 | (-9.190, 4.083) | 0.451 |
|  | Weighted median | -0.454 | 3.261 | (-6.846, 5.937) | 0.889 |
|  | Inverse variance weighted | 1.211 | 3.902 | (-6.437, 8.859) | 0.756 |

*****Bold demonstrates significantly causal association (P < 0.01)

**Supplementary Table11: Causal pairs between differentially methylated regions and differentially expressed metabolites**

| **Causal pairs** | **Method** | **Estimate** | **Standard Error** | **95% CI** | **P-value** |
| --- | --- | --- | --- | --- | --- |
| **Causality relationship: DAMs → DMRs** |  |  |  |  |  |
| **Isobutyrylcarnitine_6.110721178** | Simple median | 3.89E-06 | 1.26E-06 | (1.425e-06, 6.360e-06) | **0.002** |
|  | Weighted median | 3.76E-06 | 1.24E-06 | (1.322e-06, 6.194e-06) | **0.002** |
|  | Inverse variance weighted | 3.25E-06 | 9.10E-07 | (1.464e-06, 5.032e-06) | **0.0003** |
|  | MR-Egger | 9.00E-06 | 4.22E-06 | (7.323e-07, 1.728e-05) | 0.033 |
|  | PRESSO MR | 3.57E-06 | 9.97E-07 | (1.620e-06, 5.526e-06) | **0.009** |
|  | MR-Egger Intercept | -11.649 | 8.341 | (-28.000, 4.698) | 0.163 |
|  | PRESSO Global test |  |  |  | 1 |
| 6.110721178_Isobutyrylcarnitine | Simple median | 1122.78 | 4439.021 | (-7577.541, 9823.100) | 0.8 |
|  | Weighted median | 1776.584 | 4118.521 | (-6295.570, 9848.738) | 0.666 |
|  | Inverse variance weighted | 884.817 | 2956.581 | (-4909.975, 6679.610) | 0.765 |
|  |  |  |  |  |  |
|  |  |  |  |  |  |
| **Plasmenyl-LysoPE_6.110721178** | Simple median | -0.0002 | 9.03E-05 | (-0.0004, -8.162e-06) | 0.04 |
|  | Weighted median | -0.0002 | 8.65E-05 | (-0.0003, -1.770e-06) | 0.048 |
|  | Inverse variance weighted | -0.0002 | 6.69E-05 | (-0.0003, -4.015e-05) | **0.01** |
|  | PRESSO MR | -0.0002 | 2.17E-05 | (-0.0002, -0.00016) | **0.003** |
|  | MR-Egger Intercept | -11.43 | 16.028 | (-42.844, 19.985) | 0.476 |
|  | PRESSO Global test |  |  |  | 1 |
| 6.110721178_Plasmenyl-LysoPE | Simple median | 77.864 | 96.221 | (-110.726, 266.455) | 0.418 |
|  | Weighted median | 123.748 | 89.379 | (-51.432, 298.927) | 0.166 |
|  | Inverse variance weighted | 46.985 | 64.884 | (-80.185, 174.155) | 0.469 |
|  |  |  |  |  |  |
| **3-(2-HYDROXYPHENYL)PROPANOATE_6.110721178** | Simple median | -9.31E-06 | 2.73E-06 | (-1.467e-05, -3.959e-06) | **0.0007** |
|  | Weighted median | -4.71E-06 | 2.18E-06 | (-8.976e-06, -4.400e-07) | 0.031 |
|  | Inverse variance weighted | -4.96E-06 | 2.14E-06 | (-9.159e-06, -7.674e-07) | 0.02 |
|  | PRESSO MR | -9.85E-06 | 7.16E-07 | (-1.125e-05, -8.44e-06) | **7.53E-07** |
|  | MR-Egger Intercept | -1.92 | 7.441 | (-16.504, 12.663) | 0.796 |
|  | PRESSO Global test |  |  |  | 1 |
| 6.110721178_3-(2-HYDROXYPHENYL)PROPANOATE | Simple median | 4265.597 | 2767.74 | (-1159.073, 9690.267) | 0.123 |
|  | Weighted median | 2895.847 | 2405.585 | (-1819.013, 7610.707) | 0.229 |
|  | Inverse variance weighted | 2275.552 | 2207.966 | (-2051.982, 6603.086) | 0.303 |
|  |  |  |  |  |  |
| **N-ACETYLNEURAMINATE_6.110721178** | Simple median | -2.56E-05 | 1.11E-05 | (-4.733e-05, -3.833e-06) | 0.021 |
|  | Weighted median | -2.76E-05 | 9.65E-06 | (-4.651e-05, -8.676e-06) | **0.004** |
|  | PRESSO MR | -2.32E-05 | 6.61E-06 | (-3.617e-05, -1.025e-05) | **0.004** |
|  | MR-Egger Intercept | 16.635 | 12.398 | (-7.664, 40.934) | 0.18 |
|  | PRESSO Global test |  |  |  | 1 |
| 6.110721178_ N-ACETYLNEURAMINATE_ | Simple median | -525.342 | 643.67 | (-1786.912, 736.228) | 0.414 |
|  | Weighted median | 597.236 | 567.539 | (-515.120, 1709.593) | 0.293 |
|  | Inverse variance weighted | 48.666 | 464.701 | (-862.131, 959.462) | 0.917 |
|  |  |  |  |  |  |
| **INDOLE-3-ACETATE_6.110721178** | Simple median | 7.02E-05 | 2.60E-05 | (1.915e-05, 0.0001) | **0.007** |
|  | Weighted median | 3.75E-05 | 1.62E-05 | (5.866e-06, 6.920e-05) | 0.02 |
|  | Inverse variance weighted | 3.60E-05 | 1.68E-05 | (2.972e-06, 6.897e-05) | 0.033 |
|  | PRESSO MR | 6.14E-05 | 2.61E-05 | (1.029e-05, 0.0001) | 0.043 |
|  | MR-Egger Intercept | 4.859 | 5.118 | (-5.173, 14.891) | 0.342 |
|  | PRESSO Global test |  |  |  | 1 |
| 6.110721178_INDOLE-3-ACETATE | Simple median | 42.598 | 223.865 | (-396.168, 481.365) | 0.849 |
|  | Weighted median | -18.067 | 209.126 | (-427.946, 391.812) | 0.931 |
|  | Inverse variance weighted | -36.579 | 139.361 | (-309.722, 236.563) | 0.793 |
|  |  |  |  |  |  |
| **Phenylalanyl-Threonine_6.110721178** | Simple median | 8.47E-05 | 3.08E-05 | (2.432e-05, 0.0001) | **0.006** |
|  | Weighted median | 3.18E-05 | 1.19E-05 | (8.483e-06, 5.505e-05) | **0.007** |
|  | PRESSO MR | -9.15E-05 | 2.43E-05 | (-0.0001, -4.396e-05) | **0.005** |
|  | MR-Egger Intercept | -0.791 | 6.616 | (-13.757, 12.176) | 0.905 |
|  | PRESSO Global test |  |  |  | 1 |
| 6.110721178_ Phenylalanyl-Threonine | Simple median | 77.864 | 96.221 | (-110.726, 266.455) | 0.418 |
|  | Weighted median | 123.748 | 89.379 | (-51.432, 298.927) | 0.166 |
|  | Inverse variance weighted | 46.985 | 64.884 | (-80.185, 174.155) | 0.469 |
|  |  |  |  |  |  |
| **Ursodeoxycholic Acid (UDCA)_ 6.110721178** | Simple median | 8.26E-05 | 2.64E-05 | (3.083e-05, 0.0001) | **0.002** |
|  | Weighted median | 8.18E-05 | 2.69E-05 | (2.913e-05, 0.0001) | **0.002** |
|  | MR-Egger Intercept | -4.378 | 27.282 | (-57.849, 49.094) | 0.873 |
|  | PRESSO Global test |  |  |  | 1 |
| 6.110721178_ UDCA | Simple median | -302.033 | 279.752 | (-850.336, 246.270) | 0.28 |
|  | Weighted median | -226.188 | 256.122 | (-728.177, 275.801) | 0.377 |
|  | Inverse variance weighted | -150.295 | 179.765 | (-502.627, 202.037) | 0.403 |
|  |  |  |  |  |  |
| **GLUCOSAMINE_9.13713161** | Simple median | -1.08E-05 | 3.14E-06 | (-1.693e-05, -4.619e-06) | **0.0006** |
|  | Weighted median | -9.35E-06 | 3.26E-06 | (-1.575e-05, -2.951e-06) | **0.004** |
|  | Inverse variance weighted | -9.76E-06 | 2.49E-06 | (-1.464e-05, -4.884e-06) | **8.726E-05** |
|  | MR Egger | -2.19E-05 | 1.01E-05 | (-4.159e-05, -2.153e-06) | 0.03 |
|  | PRESSO MR | -1.37E-05 | 1.51E-06 | (-1.664e-05, -1.074e-05) | **7.82E-06** |
|  | MR Egger Intercept | 11.931 | 9.62 | (-6.924, 30.786) | 0.215 |
|  | PRESSO Global test |  |  |  | 1 |
| 9.13713161_GLUCOSAMINE | Simple median | -752.986 | 3481.158 | (-7575.931, 6069.960) | 0.829 |
|  | Weighted median | -119.539 | 2969.517 | (-5939.686, 5700.608) | 0.968 |
|  | Inverse variance weighted | -732.221 | 2201.746 | (-5047.564, 3583.122) | 0.739 |
|  |  |  |  |  |  |
| **Isobutyrylcarnitine_9.13713161** | Simple median | 5.17E-06 | 1.99E-06 | (1.268e-06, 9.065e-06) | **0.009** |
|  | Weighted median | 4.58E-06 | 1.98E-06 | (6.871e-07, 8.463e-06) | 0.021 |
|  | PRESSO MR | 4.23E-06 | 1.16E-06 | (1.954e-06, 6.512e-06) | **0.008** |
|  | MR Egger Intercept | 10.338 | 20.244 | (-29.338, 50.015) | 0.61 |
|  | PRESSO Global test |  |  |  | 1 |
| 9.13713161_Isobutyrylcarnitine | Simple median | -7002.225 | 7717.478 | (-22128.205, 8123.755) | 0.364 |
|  | Weighted median | 119.662 | 6174.522 | (-11982.178, 12221.503) | 0.985 |
|  | Inverse variance weighted | -1395.872 | 5930.35 | (-13019.145, 10227.401) | 0.814 |
|  |  |  |  |  |  |
| **ASPARTATE_9.13713161** | Simple median | -1.73E-05 | 7.23E-06 | (-3.145e-05, -3.106e-06) | 0.017 |
|  | Weighted median | -1.73E-05 | 6.95E-06 | (-3.089e-05, -3.636e-06) | 0.013 |
|  | MR Egger Intercept | 51.422 | 36.717 | (-20.542, 123.386) | 0.161 |
|  | PRESSO Global test |  |  |  | 1 |
| 9.13713161_ ASPARTATE | Simple median | 660.026 | 2609.051 | (-4453.620, 5773.672) | 0.8 |
|  | Weighted median | -1776.702 | 2053.692 | (-5801.864, 2248.460) | 0.387 |
|  | Inverse variance weighted | 198.8 | 1626.951 | (-2989.965, 3387.565) | 0.903 |
|  |  |  |  |  |  |
| **Plasmenyl-LysoPE_9.13713161** | Simple median | -0.0003 | 0.0001 | (-0.0005, -5.424e-05) | 0.013 |
|  | Weighted median | -0.0003 | 0.0001 | (-0.0005, -4.155e-05) | 0.019 |
|  | MR Egger Intercept | 27.27 | 22.153 | (-16.149, 70.688) | 0.218 |
|  | PRESSO Global test |  |  |  | 1 |
| 9.13713161_ Plasmenyl-LysoPE | Simple median | -5.593 | 166.237 | (-331.411, 320.225) | 0.973 |
|  | Weighted median | 98.285 | 135.205 | (-166.712, 363.281) | 0.467 |
|  | Inverse variance weighted | 104.935 | 109.812 | (-110.292, 320.162) | 0.339 |
|  |  |  |  |  |  |
| **N-ACETYLNEURAMINATE_9.13713161** | Simple median | 3.62E-05 | 1.40E-05 | (8.824e-06, 6.353e-05) | **0.01** |
|  | Weighted median | 3.59E-05 | 1.33E-05 | (9.858e-06, 6.192e-05) | **0.007** |
|  | Inverse variance weighted | 2.90E-05 | 1.01E-05 | (9.271e-06, 4.871e-05) | **0.004** |
|  | MR Egger Intercept | -19.292 | 23.501 | (-65.354, 26.770) | 0.412 |
|  | PRESSO Global test |  |  |  | 1 |
| 9.13713161_ N-ACETYLNEURAMINATE | Simple median | -66.524 | 893.79 | (-1818.320, 1685.273) | 0.941 |
|  | Weighted median | 228.949 | 775.877 | (-1291.742, 1749.641) | 0.768 |
|  | Inverse variance weighted | 366.278 | 597.223 | (-804.257, 1536.814) | 0.54 |
|  |  |  |  |  |  |
| **INDOLE-3-ACETATE_9.13713161** | Simple median | 8.66E-05 | 4.35E-05 | (1.373e-06, 0.0002) | 0.046 |
|  | Weighted median | 8.66E-05 | 4.36E-05 | (1.106e-06, 0.0002) | 0.047 |
|  | MR Egger Intercept | -15.276 | 50.097 | (-113.464, 82.911) | 0.76 |
|  | PRESSO Global test |  |  |  | 1 |
| 9.13713161_ INDOLE-3-ACETATE | Simple median | 316.161 | 351.893 | (-373.536, 1005.858) | 0.369 |
|  | Weighted median | 279.172 | 286.21 | (-281.789, 840.133) | 0.329 |
|  | Inverse variance weighted | 332.335 | -82.383 | (-82.383, 747.052) | 0.116 |
|  |  |  |  |  |  |
| **UDCA_ 6.163743051** | Simple median | -0.0001 | 4.19E-05 | (-0.0002, -2.684e-05) | **0.009** |
|  | Weighted median | -0.0001 | 3.89E-05 | (-0.0002, -2.998e-05) | **0.006** |
|  | Inverse variance weighted | -9.40E-05 | 2.91E-05 | (-0.0002, -3.694e-05) | **0.001** |
|  | PRESSO MR | -9.06E-05 | 3.54E-05 | (-0.0002, -2.121e-05) | 0.043 |
|  | MR Egger Intercept | -0.18 | 18.444 | (-36.329, 35.969) | 0.992 |
|  | PRESSO Global test |  |  |  | 1 |
| 6.163743051_ UDCA | Simple median | -302.033 | 279.752 | (-850.336, 246.270) | 0.28 |
|  | Weighted median | -226.188 | 256.122 | (-728.177, 275.801) | 0.377 |
|  | Inverse variance weighted | -150.295 | 179.765 | (-502.627, 202.037) | 0.403 |
|  |  |  |  |  |  |
| **Phenylalanyl-Threonine_6.163743051** | Simple median | 0.0001 | 4.58E-05 | (5.157e-05, 0.0002) | **0.002** |
|  | Weighted median | 4.73E-05 | 1.80E-05 | (1.214e-05, 8.251e-05) | **0.008** |
|  | PRESSO MR | -0.0002 | 1.88E-05 | (-0.0002, -0.00016) | **6.37E-07** |
|  | MR Egger Intercept | -0.457 | 8.373 | (-16.868, 15.955) | 0.957 |
|  | PRESSO Global test |  |  |  | 1 |
| 6.163743051_ Phenylalanyl-Threonine | Simple median | 26.455 | 200.959 | (-367.418, 420.328) | 0.895 |
|  | Weighted median | 26.455 | 191.735 | (-323.726, 427.860) | 0.786 |
|  | Inverse variance weighted | 52.067 | 136.92 | (-273.848, 262.870) | 0.968 |
|  |  |  |  |  |  |
| **INDOLE-3-ACETATE_6.163743051** | Simple median | 0.0001 | 5.42E-05 | (2.873e-05, 0.0002) | 0.013 |
|  | Weighted median | 0.0001 | 5.17E-05 | (3.250e-05, 0.0002) | **0.0002** |
|  | Inverse variance weighted | 0.0001 | 4.54E-05 | (1.512e-05, 0.0002) | 0.022 |
|  | PRESSO MR | 0.0001 | 4.33E-05 | (1.511e-05, 0.0002) | 0.04 |
|  | MR Egger Intercept | 4.689 | 16.92 | (-28.474, 37.8526) | 0.782 |
|  | PRESSO Global test |  |  |  | 1 |
| 6.163743051_ INDOLE-3-ACETATE | Simple median | 42.598 | 223.865 | (-396.168, 481.365) | 0.849 |
|  | Weighted median | -18.067 | 209.126 | (-427.946, 391.812) | 0.931 |
|  | Inverse variance weighted | -36.579 | 139.361 | (-309.722, 236.563) | 0.793 |
|  |  |  |  |  |  |
| **N-ACETYLNEURAMINATE_6.163743051** | Simple median | 3.99E-05 | 1.60E-05 | (8.587e-06, 7.127e-05) | 0.013 |
|  | PRESSO MR | -5.02E-05 | 1.24E-05 | (-7.451e-05, -2.583e-05) | **0.001** |
|  | MR Egger Intercept | -13.852 | 17.299 | (-47.756, 20.053) | 0.423 |
|  | PRESSO Global test |  |  |  | 1 |
| 6.163743051_ N-ACETYLNEURAMINATE | Simple median | -525.342 | 643.67 | (-1786.912, 736.228) | 0.414 |
|  | Weighted median | 597.236 | 567.539 | (-515.120, 1709.593) | 0.293 |
|  | Inverse variance weighted | 48.666 | 464.701 | (-862.131, 959.462) | 0.917 |
|  |  |  |  |  |  |
| **3-(2-HYDROXYPHENYL)PROPANOATE_6.163743051** | Simple median | 1.23E-05 | 5.05E-06 | (2.376e-06, 2.217e-05) | 0.015 |
|  | Weighted median | 9.50E-06 | 4.84E-06 | (1.794e-08, 1.898e-05) | 0.0496 |
|  | Inverse variance weighted | 1.17E-05 | 3.69E-06 | (4.479e-06, 1.896e-05) | **0.002** |
|  | PRESSO MR | 1.34E-05 | 1.98E-06 | (9.495e-06, 1.726e-05) | **0.001** |
|  | MR Egger Intercept | 15.522 | 19.848 | (-23.380, 54.425) | 0.434 |
|  | PRESSO Global test |  |  |  | 1 |
| 6.163743051_3-(2-HYDROXYPHENYL)PROPANOATE | Simple median | 4265.597 | 2767.74 | (-1159.073, 9690.267) | 0.123 |
|  | Weighted median | 2895.847 | 2405.585 | (-1819.013, 7610.707) | 0.229 |
|  | Inverse variance weighted | 2275.552 | 2207.967 | (-2051.982, 6603.086) | 0.303 |
|  |  |  |  |  |  |
| **Plasmenyl-LysoPE_6.163743051** | Simple median | 0.0003 | 0.0001 | (2.545e-05, 0.0006) | 0.032 |
|  | Weighted median | 0.0003 | 0.0001 | (3.175e-05, 0.0006) | 0.029 |
|  | MR Egger Intercept | 23.997 | 19.911 | (-15.028, 63.021) | 0.228 |
|  | PRESSO Global test |  |  |  | 1 |
| 6.163743051_ Plasmenyl-LysoPE | Simple median | 77.864 | 96.221 | (-110.726, 266.455) | 0.418 |
|  | Weighted median | 123.748 | 89.379 | (-51.432, 298.927) | 0.166 |
|  | Inverse variance weighted | 46.985 | 64.884 | (-80.185, 174.155) | 0.469 |
|  |  |  |  |  |  |
| **GLUCOSAMINE_6.163743051** | Weighted median | -1.27E-05 | 6.01E-06 | (-2.446e-05, -8.862e-07) | 0.035 |
|  | MR-Egger | -3.89E-05 | 1.37E-05 | (-6.585e-05, -1.203e-05) | **0.005** |
|  | PRESSO Global test |  |  |  | 1 |
| 6.163743051_ GLUCOSAMINE | Simple median | -1595.384 | 2326.139 | (-6154.533, 2963.765) | 0.493 |
|  | Weighted median | -63.83 | 2182.708 | (-4341.860, 4214.200) | 0.977 |
|  | Inverse variance weighted | -1645.604 | 1610.422 | (-4801.973, 1510.765) | 0.307 |
|  |  |  |  |  |  |
| **UDCA_ 6.110721154** | Simple median | 8.07E-05 | 2.43E-05 | (3.303e-05, 0.0001) | **0.0009** |
|  | Weighted median | 7.36E-05 | 2.33E-05 | (2.781e-05, 0.0001) | **0.0001** |
|  | Inverse variance weighted | 5.12E-05 | 1.97E-05 | (1.262e-05, 8.972e-05) | **0.009** |
|  | MR Egger Intercept | -0.386 | 12.052 | (-24.008, 23.236) | 0.974 |
|  | PRESSO Global test |  |  |  | 1 |
| 6.110721154_ UDCA | Simple median | -213.283 | 382.818 | (-963.592, 537.027) | 0.577 |
|  | Weighted median | -305.468 | 347.535 | (-986.625, 375.689) | 0.379 |
|  | Inverse variance weighted | -300.466 | 254.216 | (-798.719, 197.788) | 0.237 |
|  |  |  |  |  |  |
| **N-Methyl-D-Aspartic Acid (NMDA)_6.110721154** | Simple median | -4.84E-07 | 2.21E-07 | (-9.168e-07, -5.055e-08) | 0.029 |
|  | Weighted median | -4.84E-07 | 2.21E-07 | (-9.168e-07, -5.055e-08) | 0.027 |
|  | Inverse variance weighted | -4.44E-07 | 2.18E-07 | (-9.114e-07, -5.641e-08) | **0.009** |
|  | MR Egger Intercept | -7.353 | 36.758 | (-79.397, 64.691) | 0.841 |
|  | PRESSO Global test |  |  |  | 1 |
| 6.110721154_ NMDA | Simple median | 118137 | 90285.108 | (-58818.558, 295092.562) | 0.191 |
|  | Weighted median | 2354.348 | -157425.924 | (-157425.924, 162134.621) | 0.977 |
|  | Inverse variance weighted | 17665.318 | -104723.723 | (-104723.723, 140054.360) | 0.777 |
|  |  |  |  |  |  |
| **Phenylalanyl-Threonine_6.110721154** | Simple median | 5.43E-05 | 2.56E-05 | (4.109e-06, 0.0001) | 0.034 |
|  | Weighted median | 3.89E-05 | 1.36E-05 | (1.223e-05, 6.552e-05) | **0.004** |
|  | MR Egger Intercept | -4.851 | 7.448 | (-19.448, 9.747) | 0.515 |
|  | PRESSO Global test |  |  |  | 1 |
| 6.110721154_ Phenylalanyl-Threonine | Simple median | 52.067 | 323.849 | (-582.666, 686.799) | 0.872 |
|  | Weighted median | -16.417 | 312.145 | (-628.211, 595.376) | 0.958 |
|  | Inverse variance weighted | -168.064 | 265.596 | (-688.623, 352.495) | 0.527 |
|  |  |  |  |  |  |
| **INDOLE-3-ACETATE_6.110721154** | Simple median | 6.71E-05 | 2.93E-05 | (9.672e-06, 0.0001) | 0.022 |
|  | Weighted median | 5.89E-05 | 2.63E-05 | (7.266e-06, 0.0001) | 0.025 |
|  | Inverse variance weighted | 5.56E-05 | 2.44E-05 | (7.777e-06, 0.0001) | 0.023 |
|  | MR Egger Intercept | -3.011 | 9.556 | (-21.740, 15.719) | 0.753 |
|  | PRESSO Global test |  |  |  | 1 |
| 6.110721154_ INDOLE-3-ACETATE | Simple median | 76.17 | 324.353 | (-559.550, 711.891) | 0.814 |
|  | Weighted median | -25.355 | 310.973 | (-634.851, 584.141) | 0.935 |
|  | Inverse variance weighted | -34.88 | 271.582 | (-567.171, 497.410) | 0.898 |
|  |  |  |  |  |  |
| **N-ACETYLNEURAMINATE_6.110721154** | Simple median | -3.36E-05 | 9.82E-06 | (-5.286e-05, -1.436e-05) | **0.0006** |
|  | Weighted median | -3.36E-05 | 9.82E-06 | (-5.283e-05, -1.432e-05) | **0.0006** |
|  | MR Egger Intercept | -6.551 | 13.688 | (-33.379, 20.278) | 0.632 |
|  | PRESSO Global test |  |  |  | 1 |
| 6.110721154_ N-ACETYLNEURAMINATE | Simple median | -485.947 | 857.643 | (-2166.896, 1195.002) | 0.571 |
|  | Weighted median | -433.008 | 817.036 | (-2034.369, 1168.353) | 0.596 |
|  | Inverse variance weighted | -631.742 | 610.943 | (-1829.168, 565.684) | 0.301 |
|  |  |  |  |  |  |
| **3-(2-HYDROXYPHENYL)PROPANOATE_6.110721154** | Weighted median | -7.78E-06 | 3.39E-06 | (-1.442e-05, -1.130e-06) | 0.022 |
|  | PRESSO MR | -1.02E-05 | 7.94E-07 | (-1.180e-05, -8.685e-06) | **1.23E-06** |
|  | MR Egger Intercept | -5.128 | 9.478 | (-23.704, 13.449) | 0.589 |
|  | PRESSO Global test |  |  |  | 1 |
| 6.110721154_3-(2-HYDROXYPHENYL)PROPANOATE | Simple median | -4266.807 | 3619.036 | (-11359.988, 2826.374) | 0.238 |
|  | Weighted median | -2925.104 | 3414.916 | (-9618.216, 3768.009) | 0.392 |
|  | Inverse variance weighted | -1848.896 | 2702.464 | (-7145.629, 3447.836) | 0.494 |
|  |  |  |  |  |  |
| **Plasmenyl-LysoPE_6.110721154** | Simple median | -0.0002 | 9.81E-05 | (-0.0004, -1.699e-05) | 0.0329 |
|  | Weighted median | -0.0002 | 9.34E-05 | (-0.0004, -4.863e-06) | 0.044 |
|  | Inverse variance weighted | -0.0002 | 7.15E-05 | (-0.0003, -5.121e-05) | **0.007** |
|  | MR Egger Intercept | -11.226 | 15.133 | (-40.885, 18.434) | 0.458 |
|  | PRESSO Global test |  |  |  | 1 |
| 6.110721154_ Plasmenyl-LysoPE | Simple median | 70.333 | 145.564 | (-214.968, 355.634) | 0.629 |
|  | Weighted median | 233.251 | 130.835 | (-23.181, 489.683) | 0.075 |
|  | Inverse variance weighted | 89.534 | 91.045 | (-88.906, 267.985) | 0.325 |
|  |  |  |  |  |  |
| **ASPARTATE_6.110721154** | Simple median | -1.32E-05 | 6.41E-06 | (-2.578e-05, -6.758e-07) | 0.039 |
|  | Inverse variance weighted | -1.44E-05 | 4.92E-06 | (-2.405e-05, -4.773e-06) | **0.003** |
|  | PRESSO MR | -1.50E-05 | 2.50E-06 | (-1.989e-05, -1.007e-05) | **0.009** |
|  | MR Egger Intercept | 8.321 | 20.419 | (-31.699, 48.342) | 0.684 |
|  | PRESSO Global test |  |  |  | 1 |
| 6.110721154_ ASPARTATE | Simple median | 2747.406 | 2149.372 | (-1465.286, 6960.098) | 0.201 |
|  | Weighted median | 3192.958 | 2031.303 | (-788.323, 7174.240) | 0.116 |
|  | Inverse variance weighted | 1886.213 | 1491.042 | (-1036.176, 4808.602) | 0.206 |
|  |  |  |  |  |  |
| **Isobutyrylcarnitine_6.110721154** | Simple median | 5.08E-06 | 1.71E-06 | (1.727e-06, 8.433e-06) | **0.003** |
|  | Weighted median | 2.55E-06 | 9.14E-07 | (7.549e-07, 4.336e-06) | **0.005** |
|  | Inverse variance weighted | 2.71E-06 | 7.69E-07 | (1.2074e-06, 4.219e-06) | **4.00E-04** |
|  | PRESSO MR | 5.04E-06 | 5.12E-07 | (4.040e-06, 6.048e-06) | **6.33E-05** |
|  | PRESSO Global test |  |  |  | 1 |
| 6.110721154_ Isobutyrylcarnitine | Simple median | -585.729 | 7063.527 | (-14429.988, 13258.530) | 0.934 |
|  | Weighted median | -290.961 | 6658.545 | (-13341.468, 12759.547) | 0.965 |
|  | Inverse variance weighted | -2256.89 | 4665.966 | (-11402.014, 6888.235) | 0.629 |
|  |  |  |  |  |  |
| **GLUCOSAMINE_6.110721154** | Simple median | -1.02E-05 | 3.34E-06 | (-1.676e-05, -3.669e-06) | **0.002** |
|  | Weighted median | -9.78E-06 | 3.36E-06 | (-1.636e-05, -3.201e-06) | **0.004** |
|  | MR Egger Intercept | -0.768 | 13.692 | (-27.604, 26.068) | 0.955 |
|  | PRESSO Global test |  |  |  | 1 |
| 6.110721154_ GLUCOSAMINE | Simple median | 296.302 | 3181.984 | (-5940.271, 6532.876) | 0.926 |
|  | Weighted median | -1827.858 | 2978.241 | (-7665.103, 4009.388) | 0.539 |
|  | Inverse variance weighted | -118.39 | 2096.397 | (-4227.253, 3990.473) | 0.955 |
|  |  |  |  |  |  |
| **GLUCOSAMINE_6.110721139** | Simple median | -9.76E-06 | 3.15E-06 | (-1.592e-05, -3.596e-06) | **0.002** |
|  | Weighted median | -9.66E-06 | 3.16E-06 | (-1.585e-05, -3.473e-06) | **0.002** |
|  | MR Egger Intercept | 11.285 | 10.806 | (-9.895, 32.465) | 0.296 |
|  | PRESSO Global test |  |  |  | 1 |
| 6.110721139_ GLUCOSAMINE | Simple median | -505.214 | 3344.164 | (-7059.654, 6049.227) | 0.88 |
|  | Weighted median | 1776.865 | 3050.824 | (-4202.640, 7756.370) | 0.56 |
|  | Inverse variance weighted | 639.807 | 2284.942 | (-3838.597, 5118.212) | 0.779 |
|  |  |  |  |  |  |
| **Isobutyrylcarnitine_6.110721139** | Simple median | 4.71E-06 | 1.73E-06 | (1.330e-06, 8.099e-06) | **0.006** |
|  | Weighted median | 2.48E-06 | 8.74E-07 | (7.697e-07, 4.194e-06) | **0.004** |
|  | Inverse variance weighted | 2.68E-06 | 8.05E-07 | (1.105e-06, 4.261e-06) | **0.0009** |
|  | PRESSO MR | 4.91E-06 | 6.59E-07 | (3.616e-06, 6.198e-06) | **0.0003** |
|  | PRESSO Global test |  |  |  | 1 |
| 6.110721139_ Isobutyrylcarnitine | Simple median | -2273.413 | 6984.352 | (-15962.491, 11415.665) | 0.745 |
|  | Weighted median | -10617.93 | 6505.875 | (-23369.208, 2133.352) | 0.103 |
|  | Inverse variance weighted | -7808.178 | 4781.639 | (-17180.018, 1563.662) | 0.102 |
|  |  |  |  |  |  |
| **3-(2-HYDROXYPHENYL)PROPANOATE_6.110721139** | Simple median | -9.06E-06 | 2.67E-06 | (-1.429e-05, -3.829e-06) | **0.0007** |
|  | Weighted median | -2.29E-06 | 9.14E-07 | (-4.080e-06, -4.995e-07) | 0.012 |
|  | Inverse variance weighted | -2.64E-06 | 1.02E-06 | (-4.636e-06, -6.358e-07) | **0.01** |
|  | PRESSO MR | -8.95E-06 | 8.54E-07 | (-1.063e-05, -7.280e-06) | **1.03E-06** |
|  | MR Egger Intercept | -2.806 | 3.999 | (-10.643, 5.031) | 0.483 |
|  | PRESSO Global test |  |  |  | 1 |
| 6.110721139_3-(2-HYDROXYPHENYL)PROPANOATE | Simple median | 3523.242 | 3305.316 | (-2955.058, 10001.542) | 0.286 |
|  | Weighted median | 4885.748 | 3192.638 | (-1371.707, 11143.203) | 0.126 |
|  | Inverse variance weighted | 3920.685 | 2403.581 | (-790.246, 8631.617) | 0.103 |
|  |  |  |  |  |  |
| **Phenylalanyl-Threonine_6.110721139** | Simple median | -9.50E-05 | 3.63E-05 | (-0.0002, -2.387e-05) | **0.009** |
|  | Weighted median | -9.47E-05 | 3.77E-05 | (-0.0002, -2.080e-05) | 0.012 |
|  | PRESSO MR | -0.0001 | 1.37E-05 | (-0.0001, -7.315e-05) | **0.0003** |
|  | MR Egger Intercept | 39.558 | 27.077 | (-13.512, 92.628) | 0.144 |
|  | PRESSO Global test |  |  |  | 1 |
| 6.110721139_ Phenylalanyl-Threonine | Simple median | -245.682 | 309.724 | (-852.729, 361.365) | 0.428 |
|  | Weighted median | -277.623 | 310.581 | (-886.350, 331.105) | 0.371 |
|  | Inverse variance weighted | -373.159 | 222.852 | (-809.941, 63.624) | 0.094 |
|  |  |  |  |  |  |
| **UDCA_ 6.110721139** | Simple median | 9.42E-05 | 2.51E-05 | (4.504e-05, 0.0001) | **0.0002** |
|  | Weighted median | 6.39E-05 | 2.37E-05 | (1.738e-05, 0.0001) | **0.007** |
|  | Inverse variance weighted | 6.05E-05 | 1.70E-05 | (2.723e-05, 9.369e-05) | **0.0004** |
|  | MR Egger Intercept | 2.792 | 10.338 | (-17.470, 23.055) | 0.787 |
|  | PRESSO Global test |  |  |  | 1 |
| 6.110721139_ UDCA | Simple median | 6.109 | 393.359 | (-764.860, 777.079) | 0.988 |
|  | Weighted median | 153.329 | 367.49 | (-566.937, 873.596) | 0.677 |
|  | Inverse variance weighted | 237.149 | 276.986 | (-305.733, 780.032) | 0.392 |
|  |  |  |  |  |  |
| **UDCA_ 11.129594021** | Simple median | 7.11E-05 | 2.84E-05 | (1.539e-05, 0.0001) | 0.012 |
|  | Weighted median | 1.44E-05 | 6.84E-06 | (1.037e-06, 2.783e-05) | 0.035 |
|  | PRESSO MR | -6.93E-05 | 1.79E-05 | (-0.0001, -3.427e-05) | **0.003** |
|  | MR Egger Intercept | -0.816 | 4.973 | (-10.563, 8.931) | 0.87 |
|  | PRESSO Global test |  |  |  |  |
| 11.129594021_UDCA | Simple median | -592.903 | 451.808 | (-1478.429, 292.624) | 0.189 |
|  | Weighted median | 399.101 | 318.446 | (-225.041, 1023.243) | 0.21 |
|  | Inverse variance weighted | 37.862 | 303.203 | (-556.405, 632.129) | 0.9 |
|  |  |  |  |  |  |
| **Phenylalanyl-Threonine_1.1129594021** | Simple median | -0.0001 | 4.69E-05 | (-0.0002, -1.768e-05) | 0.019 |
|  | Weighted median | -3.95E-05 | 1.60E-05 | (-7.091e-05, -8.099e-06) | 0.014 |
|  | Inverse variance weighted | -4.67E-05 | 1.28E-05 | (-7.182e-05, -2.156e-05) | **0.0003** |
|  | MR Egger Intercept | -9.921 | 5.378 | (-20.462, 0.620) | 0.065 |
|  | PRESSO Global test |  |  |  | 1 |
| 11.129594021_Phenylalanyl-Threonine | Simple median | 91.624 | 273.162 | (-443.764, 627.012) | 0.737 |
|  | Weighted median | -13.272 | 221.332 | (-447.074, 420.530) | 0.952 |
|  | Inverse variance weighted | 53.297 | 156.618 | (-253.668, 360.263) | 0.734 |
|  |  |  |  |  |  |
| **Isobutyrylcarnitine_11.129594021** | Simple median | 5.76E-06 | 2.09E-06 | (1.655e-06, 9.859e-06) | **0.006** |
|  | Weighted median | 5.37E-06 | 2.13E-06 | (1.200e-06, 9.533e-06) | 0.012 |
|  | MR Egger Intercept | 33.846 | 26.108 | (-17.324, 85.016) | 0.195 |
|  | PRESSO Global test |  |  |  | 1 |
| 11.129594021_Isobutyrylcarnitine | Simple median | 10268.952 | 6236.851 | (-1955.051, 22492.955) | 0.1 |
|  | Weighted median | 6190.513 | 5065.921 | (-3738.508, 16119.535) | 0.222 |
|  | Inverse variance weighted | 3820.387 | 3414.788 | (-2872.475, 10513.249) | 0.263 |
|  |  |  |  |  |  |
| **GLUCOSAMINE_11.129594021** | Simple median | 1.29E-05 | 3.42E-06 | (6.192e-06, 1.960e-05) | **0.0002** |
|  | Weighted median | 1.28E-05 | 3.29E-06 | (6.344e-06, 1.924e-05) | **0.0001** |
|  | PRESSO MR | 1.43E-05 | 9.52E-07 | (1.244e-05, 1.618e-05) | **3.78E-09** |
|  | MR Egger Intercept | 0.166 | 19.155 | (-37.377, 37.709) | 0.993 |
|  | PRESSO Global test |  |  |  | 1 |
| 11.129594021_ GLUCOSAMINE | Simple median | 4197.69 | 3131.415 | (-1939.769, 10335.150) | 0.18 |
|  | Weighted median | 2359.047 | 2849.648 | (-3226.1595, 7944.254) | 0.407 |
|  | Inverse variance weighted | 987.482 | 2270.862 | (-3463.326, 5438.290) | 0.664 |
| **Causality relationship: DMRs → DAMs** |  |  |  |  |  |
| **11.129594021_3-(2-HYDROXYPHENYL)PROPANOATE** | Weighted median | -6037.613 | 2966.197 | (-11851.252, -223.974) | 0.042 |
|  | Inverse variance weighted | -8703.781 | 2671.826 | (-13940.464, -3467.098) | **0.001** |
|  | PRESSO MR | -12089.14 | 2650.463 | (-17284.04, -6894.229) | **0.001** |
|  | MR Egger Intercept | 57902.23 | 206057.625 | (-345963.294, 461767.754) | 0.779 |
|  | PRESSO Global test |  |  |  | 1 |
| 3-(2-HYDROXYPHENYL)PROPANOATE_11.129594021 | Simple median | -8.15E-06 | 4.32E-06 | (-1.663e-05, 3.172e-07) | 0.059 |
|  | Weighted median | -1.68E-06 | 2.31E-06 | (-6.218e-06, 2.851e-06) | 0.467 |
|  | Inverse variance weighted | -1.46E-06 | 2.36E-06 | (-6.072e-06, 3.158e-06) | 0.536 |

*Bold demonstrates significantly causal association (*P* < 0.01).

**Supplementary Table12: Causal pairs between hub genes and differentially expressed metabolites**

| **Causal pairs** | **Method** | **Estimate** | **Standard Error** | **95% CI** | **P-value** |
| --- | --- | --- | --- | --- | --- |
| **Causality relationship: Genes → DAMs** |  |  |  |  |  |
| **ANO6_fructose** | Inverse variance weighted | -13.732 | 4.764 | (-23.070, -4.394) | **0.004** |
|  | PRESSO MR | -18.373 | 4.819 | (-27.818, -8.928) | **0.0005** |
|  | MR-Egger Intercept | -447.069 | 8911.296 | (-17912.889, 17018.751) | 0.96 |
|  | PRESSO Global test |  |  |  | 1 |
| fructose _ANO6 | Simple median | -0.0002 | 0.0003 | (-0.0007, 0.00033) | 0.465 |
|  | Weighted median | -0.0002 | 0.0003 | (-0.0007, 0.00033) | 0.483 |
|  | Inverse variance weighted | -5.63E-05 | 0.0002 | (-0.0004, 0.0003) | 0.76 |
| **ANO6_N-ACETYLNEURAMINATE** | Simple median | -52.611 | 20.907 | (-93.588, -11.633) | 0.012 |
|  | Weighted median | -52.332 | 21.246 | (-93.974, -10.691) | 0.014 |
|  | Inverse variance weighted | -45.74 | 14.999 | (-75.138, -16.342) | **0.002** |
|  | PRESSO MR | -53.508 | 11.046 | (-75.158, -31.858) | **1.94E-05** |
|  | MR-Egger Intercept | -23865.862 | 33026.331 | (-88596.281, 40864.557) | 0.47 |
|  | PRESSO Global test |  |  |  | 1 |
| N-ACETYLNEURAMINATE_ ANO6 | Simple median | -1.32E-05 | 3.91E-05 | (-8.983e-05, 6.348e-05) | 0.736 |
|  | Weighted median | 4.40E-05 | 3.88E-05 | (-3.214e-05, 0.0001) | 0.258 |
|  | Inverse variance weighted | 8.50E-06 | 2.45E-05 | (-3.953e-05, 5.652e-05) | 0.729 |
| **CLU_ASPARTATE** | Inverse variance weighted | 48.779 | 14.669 | (20.028, 77.530) | **0.0009** |
|  | PRESSO MR | 63.674 | 14.031 | (36.173, 91.175) | **1.90E-05** |
|  | MR Egger Intercept | 66883.818 | 86882.181 | (-103402.127, 237169.763) | 0.441 |
|  | PRESSO Global test |  |  |  | 1 |
| ASPARTATE_ CLU | Simple median | 0.0001 | 6.97E-05 | (-3.174e-05, 0.0002) | 0.132 |
|  | Weighted median | 0.0001 | 6.92E-05 | (-2.162e-05, 0.0002) | 0.099 |
|  | Inverse variance weighted | 9.69E-05 | 4.76E-05 | (3.592e-06, 0.0002) | 0.042 |
| **CLU_N-ACETYLNEURAMINATE** | Simple median | -20.399 | 7.554 | (-35.204, -5.593) | **0.007** |
|  | Weighted median | -16.988 | 7.959 | (-32.587, -1.388) | 0.033 |
|  | Inverse variance weighted | -13.348 | 5.426 | (-23.983, -2.712) | 0.014 |
|  | PRESSO MR | -19.537 | 3.495 | (-26.387, -12.687) | **2.82E-07** |
|  | MR Egger Intercept | 6541.39 | 31796.217 | (-55778.050, 68860.830) | 0.837 |
|  | PRESSO Global test |  |  |  | 1 |
| N-ACETYLNEURAMINATE_ CLU | Simple median | -0.0001 | 7.45E-05 | (-0.0003, 7.317e-06) | 0.063 |
|  | Inverse variance weighted | -8.65E-05 | 4.67E-05 | (-0.0002, 5.020e-06) | 0.064 |
| **LUZP6_fructose** | Simple median | -36.393 | 9.856 | (-55.711, -17.076) | **0.0002** |
|  | Weighted median | -35.685 | 9.899 | (-55.088, -16.282) | **0.0003** |
|  | Inverse variance weighted | -30.935 | 6.76 | (-44.183, -17.686) | **4.73E-06** |
|  | MR Egger | -49.501 | 22.762 | (-94.113, -4.890) | 0.03 |
|  | PRESSO MR | -40.167 | 5.384 | (-50.720, -29.614) | **9.83E-09** |
|  | MR Egger Intercept | 7715.57 | 9032.021 | (-9986.866, 25418.006) | 0.392 |
|  | PRESSO Global test |  |  |  |  |
| Fructose_ LUZP6 | Simple median | -0.0002 | 0.0002 | （-0.0006, 0.0002） | 0.41 |
|  | Weighted median | -0.0002 | 0.0002 | (-0.0006, 0.0002) | 0.295 |
|  | Inverse variance weighted | -0.0001 | 0.0001 | (-0.0004, 0.0002) | 0.485 |
| **LUZP6_N-ACETYLNEURAMINATE** | Simple median | -70.521 | 28.854 | (-127.074, -13.967) | 0.015 |
|  | Weighted median | -58.162 | 29.239 | (-115.471, -0.854) | 0.047 |
|  | Inverse variance weighted | -55.215 | 19.647 | -(93.723, -16.707) | **0.005** |
|  | PRESSO MR | -65.483 | 15.877 | (-96.602, -34.364) | **0.0002** |
|  | MR Egger Intercept | 0.543 | 29346.011 | (-75346.130, 39688.120) | 0.543 |
|  | PRESSO Global test |  |  |  | 1 |
| N-ACETYLNEURAMINATE _LUZP6 | Simple median | 1.59E-07 | 3.12E-05 | (-6.090e-05, 6.122e-05) | 0.996 |
|  | Weighted median | 5.06E-05 | 3.11E-05 | (-1.033e-05, 0.0001) | 0.103 |
|  | Inverse variance weighted | 1.46E-05 | 1.91E-05 | (-2.293e-05, 5.206e-05) | 0.447 |
| **MPEG1_Sphingosine** | Weighted median | 141.606 | 68.326 | 7.691, 275.522 | 0.038 |
|  | Inverse variance weighted | 120.31 | 49.685 | 22.929, 217.690 | 0.015 |
|  | MR Egger Intercept | -261221.5 | 169404.273 | (-593247.779, 70804.770) | 0.123 |
|  | PRESSO Global test |  |  |  | 1 |
| Sphingosine_ MPEG1 | Simple median | 0.0001 | 5.75E-05 | 5.629e-06, 0.0002 | 0.04 |
|  | Weighted median | 7.88E-05 | 5.65E-05 | -3.185e-05, 0.0002 | 0.163 |
|  | Inverse variance weighted | 7.51E-05 | 3.97E-05 | -2.668e-06, 0.0002 | 0.058 |
|  | MR Egger | -6.71E-06 | 0.0001 | -0.0003, 0.0003 | 0.962 |
| **PLCB2_Isobutyrylcarnitine** | Simple median | -556.868 | 197.195 | (-943.364, -170.372) | **0.005** |
|  | Weighted median | -419.496 | 195.428 | (-802.528, -36.465) | 0.032 |
|  | Inverse variance weighted | -379.371 | 139.062 | (-651.928, -106.815) | **0.006** |
|  | PRESSO MR | -436.001 | 107.674 | (-647.042, -224.96) | **0.0001** |
|  | MR Egger Intercept | -166322.99 | 237790.478 | (-632383.766, 299737.781) | 0.484 |
| Isobutyrylcarnitine_ PLCB2 | Simple median | 4.81E-06 | 4.17E-06 | (-3.360e-06, 1.297e-05) | 0.249 |
|  | Weighted median | 6.19E-06 | 4.15E-06 | (-1.935e-06, 1.432e-05) | 0.135 |
|  | Inverse variance weighted | 7.23E-06 | 2.82E-06 | (1.713e-06, 1.275e-05) | 0.01 |
|  | MR Egger | 4.41E-07 | 1.34E-05 | (-2.577e-05, 2.665e-05) | 0.974 |
| **PLCB2_Sphingosine** | Simple median | -240.751 | 85.935 | (-409.181, -72.321) | **0.005** |
|  | Inverse variance weighted | -165.011 | 57.619 | (-277.942, -52.079) | **0.004** |
|  | PRESSO MR | -167.607 | 56.863 | (-279.059, -56.156) | **0.004** |
|  | MR Egger Intercept | 20312.477 | 87489.661 | (-151164.108, 191789.062) | 0.816 |
|  | PRESSO Global test |  |  |  | 1 |
| Sphingosine _PLCB2 | Simple median | 2.20E-05 | 2.11E-05 | (-1.939e-05, 6.345e-05) | 0.297 |
|  | Weighted median | 2.29E-05 | 2.08E-05 | (-1.786e-05, 6.359e-05) | 0.271 |
|  | Inverse variance weighted | 1.39E-05 | 1.45E-05 | (-1.448e-05, 4.236e-05) | 0.336 |
| **PLCB2_UDCA** | Simple median | -33.038 | 12.061 | (-56.677, -9.398) | **0.006** |
|  | Weighted median | -35.029 | 12.078 | (-58.702, -11.356) | **0.004** |
|  | Inverse variance weighted | -30.605 | 8.58 | (-47.421, -13.789) | **0.0004** |
|  | PRESSO MR | -32.727 | 6.265 | (-45.006, -20.448) | **1.49E-06** |
|  | MR Egger Intercept | -6737.454 | 12842.62 | (-31908.527, 18433.619) | 0.6 |
|  | PRESSO Global test |  |  |  | 1 |
| UDCA_PLCB2 | Simple median | -3.01E-05 | 5.77E-05 | -0.0001, 8.310e-05 | 0.603 |
|  | Weighted median | -2.20E-06 | 5.80E-05 | -0.0001, 0.0001 | 0.97 |
|  | Inverse variance weighted | -2.37E-05 | 3.38E-05 | -8.989e-05, 4.259e-05 | 0.484 |
| **UGGT1_Phenylalanyl-Threonine** | Inverse variance weighted | 1.328 | 0.422 | (0.501, 2.154) | **0.002** |
|  | PRESSO MR | 1.612 | 0.519 | (0.595, 2.629) | **0.002** |
|  | MR Egger Intercept | 1306.714 | 2237.423 | (-3078.554, 5691.982) | 0.559 |
|  | PRESSO Global test |  |  |  | 1 |
| Phenylalanyl-Threonine _UGGT1 | Simple median | 0.0003 | 0.0005 | -0.0007, 0.001 | 0.527 |
|  | Weighted median | 0.0004 | 0.0005 | -0.0005, 0.001 | 0.354 |
|  | Inverse variance weighted | 0.0005 | 0.0003 | -2.599e-05, 0.001 | 0.062 |
| **UGGT1_UDCA** | Simple median | -2.666 | 0.976 | (-4.579, -0.753) | **0.006** |
|  | Weighted median | -2.415 | 0.943 | (-4.262, -0.568) | **0.01** |
|  | Inverse variance weighted | -1.962 | 0.546 | (-3.032, -0.892) | **0.0003** |
|  | MR Egger | -2.475 | 0.926 | (-4.291, -0.659) | **0.008** |
|  | PRESSO MR | -2.017 | 0.607 | (-3.207, -0.827) | **0.001** |
|  | MR Egger Intercept | 1943.012 | 2837.921 | (-3619.210, 7505.235) | 0.494 |
|  | PRESSO Global test |  |  |  |  |
| UDCA_ UGGT1 | Weighted median | 0.0005 | 0.0004 | (-0.0003, 0.001) | 0.232 |
|  | Inverse variance weighted | -4.81E-05 | 0.0002 | (-0.0005, 0.0004) | 0.822 |
| **IQGAP1_ASPARTATE** | Simple median | -231.99 | 90.042 | (-408.469, -55.510) | 0.01 |
|  | Weighted median | -238.506 | 88.755 | (-412.462, -64.550) | **0.007** |
|  | Inverse variance weighted | -251.296 | 60.29 | (-369.462, -133.131) | **3.07E-05** |
|  | MR Egger | -577.229 | 283.104 | (-1132.105, -22.355) | 0.041 |
|  | PRESSO MR | -306.021 | 55.095 | (-414.007, -198.035) | **6.11E-06** |
|  | MR Egger Intercept | 100205.117 | 85041.218 | (-66472.608, 266882.841) | 0.239 |
|  | PRESSO Global test |  |  |  |  |
| ASPARTATE_IQGAP1 | Simple median | -5.65E-05 | 2.97E-05 | (-0.0001, 1.793e-06) | 0.057 |
|  | Weighted median | -6.28E-05 | 2.99E-05 | (-0.0001, -4.168e-06) | 0.036 |
|  | MR Egger | 5.00E-05 | 8.87E-05 | (-0.0001, 0.0002) | 0.572 |
| **Causality relationship: DAMs → Genes** |  |  |  |  |  |
| **GLUCOSAMINE_ANO6** | Weighted median | 6.09E-05 | 1.55E-05 | (3.042e-05, 9.130e-05) | **8.91E-05** |
|  | Inverse variance weighted | 4.10E-05 | 9.86E-06 | (2.1626e-05, 6.028e-05) | **3.29E-05** |
|  | MR-Egger | 0.0001 | 2.48E-05 | (5.526e-05, 0.0002) | **2.82E-05** |
|  | PRESSO MR | 2.94E-05 | 7.90E-06 | (1.389e-05, 4.485e-05) | **0.0003** |
|  | PRESSO Global test |  |  |  | 1 |
| ANO6_GLUCOSAMINE | Simple median | 26.54 | 84.679 | (-139.427, 192.507) | 0.754 |
|  | Weighted median | -62.256 | 82.361 | (-223.681, 99.169) | 0.45 |
|  | Inverse variance weighted | -11.024 | 57.626 | (-123.969, 101.920) | 0.848 |
|  |  |  |  |  |  |
| **Isobutyrylcarnitine_ANO6** | Simple median | -2.63E-05 | 7.48E-06 | (-4.092e-05, -1.160e-05) | **0.0004** |
|  | Weighted median | -2.50E-05 | 8.55E-06 | (-4.180e-05, -8.282e-06) | **0.003** |
|  | Inverse variance weighted | -1.50E-05 | 4.99E-06 | (-2.478e-05, -5.237e-06) | **0.003** |
|  | MR-Egger | -5.26E-05 | 1.99E-05 | (-9.159e-05, -1.366e-05) | **0.008** |
|  | PRESSO MR | -1.75E-05 | 5.28E-06 | (-2.784e-05, -7.157e-06) | **0.001** |
|  | MR-Egger Intercept | 85.675 | 43.859 | (-0.286, 171.637) | 0.05 |
|  | PRESSO Global test |  |  |  | 1 |
| ANO6_ Isobutyrylcarnitine | Simple median | -213.017 | 173.463 | (-552.998, 126.964) | 0.219 |
|  | Weighted median | -140.596 | 167.666 | (-469.215, 188.023) | 0.402 |
|  | Inverse variance weighted | -85.096 | 111.926 | (-304.467, 134.275) | 0.447 |
| **NMDA_ANO6** | Simple median | -3.29E-06 | 1.51E-06 | (-6.252e-06, -3.266e-07) | 0.03 |
|  | Weighted median | -3.94E-06 | 1.41E-06 | (-6.690e-06, -1.183e-06) | **0.005** |
|  | Inverse variance weighted | -2.23E-06 | 1.06E-06 | (-4.301e-06, -1.639e-07) | 0.034 |
|  | PRESSO MR | -4.08E-06 | 1.04E-06 | (-6.124e-06, -2.032e-06) | **0.001** |
|  | MR-Egger Intercept | -16.87 | 63.738 | (-141.794, 108.054) | 0.791 |
|  | PRESSO Global test |  |  |  | 1 |
| ANO6_NMDA | Simple median | -2856.17 | 2118.546 | (-7008.444, 1296.104) | 0.178 |
|  | Weighted median | -2618.638 | 2075.897 | (-6687.322, 1450.045) | 0.207 |
|  | Inverse variance weighted | -3510.589 | 1412.926 | (-6279.874, -741.305) | 0.013 |
|  | MR Egger | -309.551 | 5261.792 | (-10622.473, 10003.371) | 0.953 |
|  |  |  |  |  |  |
| **Phenylalanyl-Threonine_ANO6** | Simple median | -0.0003 | -0.0005 | (-0.00054, -0.00052) | 0.037 |
|  | Inverse variance weighted | -0.0002 | -0.0003 | (-0.0003, -9.480e-06) | 0.037 |
|  | PRESSO MR | -0.0004 | 8.44E-05 | (-0.0006, -0.0002) | **2.36E-06** |
|  | MR-Egger Intercept | -28.067 | 17.761 | (-62.878, 6.744) | 0.114 |
|  | PRESSO Global test |  |  |  | 1 |
| ANO6_Phenylalanyl-Threonine | Simple median | 2.746 | 7.56 | (-12.072, 17.564) | 0.716 |
|  | Weighted median | -8.34 | 7.248 | (-22.545, 5.865) | 0.25 |
|  | Inverse variance weighted | -0.726 | 4.894 | (-10.318, 8.866) | 0.882 |
|  |  |  |  |  |  |
| **Plasmenyl-LysoPE_ANO6** | MR Egger | -0.003 | 0.001 | (-0.006, -0.001) | **0.005** |
|  | PRESSO MR | -0.0008 | 0.0002 | (-0.001, -0.0004) | **0.001** |
|  | PRESSO Global test |  |  |  | 1 |
| ANO6_ Plasmenyl-LysoPE | Simple median | -3.506 | 3.932 | (-11.212, 4.200) | 0.373 |
|  | Weighted median | -2.431 | 3.79 | (-9.859, 4.997) | 0.521 |
|  | Inverse variance weighted | -4.835 | 2.516 | (-9.765, 0.096) | 0.055 |
| **3-(2-HYDROXYPHENYL)PROPANOATE_ANO6** | Simple median | -4.60E-05 | 1.54E-05 | (-7.616e-05, -1.586e-05) | 0.003 |
|  | Inverse variance weighted | -1.64E-05 | 7.84E-06 | (-3.175e-05, -1.010e-06) | 0.037 |
|  | PRESSO MR | -4.00E-05 | 9.17E-06 | (-5.796e-05, -2.204e-05) | **2.91E-05** |
|  | PRESSO Global test |  |  |  | 1 |
| ANO6_3-(2-HYDROXYPHENYL)PROPANOATE | Simple median | -92.54 | 83.879 | (-256.940, 71.860) | 0.27 |
|  | Weighted median | -98.287 | 84.712 | (-264.319, 67.745) | 0.246 |
|  | Inverse variance weighted | -107.277 | 56.02 | (-217.074, 2.520) | 0.055 |
| **UDCA_ANO6** | Weighted median | -0.0003 | 0.0001 | (-0.0005, -5.106e-05) | 0.016 |
|  | PRESSO MR | -0.0002 | 6.41E-05 | (-0.0003, -7.432e-05) | **0.001** |
|  | MR Egger Intercept | 3793.787 | 15768.28 | (-27111.472, 334699.048) | 0.81 |
|  | PRESSO Global test |  |  |  | 1 |
| ANO6_ UDCA | Simple median | -9.247 | 9.792 | (-28.438, 9.945) | 0.345 |
|  | Weighted median | -9.508 | 9.491 | (-28.109, 9.093) | 0.316 |
|  | Inverse variance weighted | -4.626 | 6.719 | (-17.796, 8.543) | 0.491 |
|  |  |  |  |  |  |
| **Plasmenyl-LysoPE_CLU** | Simple median | -0.002 | 0.0008 | (-0.0032, -0.0001) | 0.036 |
|  | PRESSO MR | -0.002 | 0.0003 | (-0.003, -0.001) | **1.41E-05** |
|  | MR Egger Intercept | -60.705 | 53.461 | (-165.486, 44.077) | 0.256 |
|  | PRESSO Global test |  |  |  | 1 |
| CLU_Plasmenyl-LysoPE | Simple median | -0.77 | 1.433 | (-3.578, 2.038) | 0.591 |
|  | Weighted median | -0.868 | 1.427 | (-3.665, 1.930) | 0.543 |
|  | Inverse variance weighted | -0.427 | 0.92 | (-2.230, 1.377) | 0.643 |
| **GLUCOSAMINE_IQGAP1** | Weighted median | -3.35E-05 | 1.18E-05 | (-5.658e-05, -1.034e-05) | **0.005** |
|  | Inverse variance weighted | -2.09E-05 | 7.10E-06 | (-3.484e-05, -7.008e-06) | **0.003** |
|  | MR Egger | -3.85E-05 | 1.30E-05 | (-6.392e-05, -1.313e-05) | **0.003** |
|  | PRESSO MR | -2.48E-05 | 6.57E-06 | (-3.768e-05, -1.192e-05) | **0.0003** |
|  | MR Egger Intercept | 27.993 | 17.236 | (-5.788, 61.774) | 0.104 |
|  | PRESSO Global test |  |  |  | 1 |
| IQGAP1_GLUCOSAMINE | Simple median | 148.776 | 126.235 | (-98.640, 396.192) | 0.239 |
|  | Weighted median | 171.942 | 122.933 | (-69.002, 412.886) | 0.162 |
|  | Inverse variance weighted | 112.832 | 83.689 | (-51.196, 276.861) | 0.178 |
|  |  |  |  |  |  |
| INDOLE-3-ACETATE_IQGAP1 | Inverse variance weighted | 0.0002 | 0.0001 | (1.790e-05, 0.0004) | 0.033 |
|  | PRESSO MR | 0.0002 | 9.64E-05 |  | 0.012 |
|  | MR Egger Intercept | -55.753 | 41.727 | (-137.536, 26.030) | 0.182 |
|  | PRESSO Global test |  |  |  | 1 |
| IQGAP1_ INDOLE-3-ACETATE | Simple median | 11.865 | 12.211 | (-12.067, 35.798) | 0.331 |
|  | Weighted median | 14.713 | 12.119 | (-9.040, 38.465) | 0.225 |
|  | Inverse variance weighted | 17.449 | 8.641 | (0.513, 34.385) | 0.043 |
|  | MR Egger | 16.929 | 41.878 | (-65.150, 99.007) | 0.686 |
| **N-ACETYLNEURAMINATE_IQGAP1** | Weighted median | -9.96E-05 | -0.0002 | (-0.0002, -2.838e-05) | **0.006** |
|  | MR Egger | -0.0001 | 5.77E-05 | (-0.0002, -4.021e-06) | 0.042 |
|  | PRESSO MR | 6.75E-05 | 2.05E-05 | (2.737e-05, 0.0001) | **0.001** |
|  | MR Egger Intercept | 29.925 | 18.221 | (-5.789, 65.638) | 0.101 |
|  | PRESSO Global test |  |  |  | 1 |
| IQGAP1_ N-ACETYLNEURAMINATE | Simple median | 52.846 | 32.645 | (-11.136, 116.829) | 0.105 |
|  | Weighted median | 48.339 | 31.793 | (-13.974, 110.653) | 0.128 |
|  | Inverse variance weighted | 36.22 | 23.019 | (-8.896, 81.336) | 0.116 |
|  |  |  |  |  |  |
| **Phenylalanyl-Threonine_IQGAP1** | Simple median | -0.0005 | 0.0001 | (-0.0007, -0.0002) | **0.0002** |
|  | Inverse variance weighted | -0.0002 | 6.38E-05 | (-0.0003, -7.942e-05) | **0.001** |
|  | MR Egger | 0.0003 | 0.0001 | (6.427e-05, 0.0006) | 0.013 |
|  | PRESSO MR | -0.0005 | 7.75E-05 | (-0.0007, -0.0003) | **1.97E-08** |
|  | MR Egger Intercept |  |  |  | 1 |
|  | PRESSO Global test |  |  |  |  |
| IQGAP1_ Phenylalanyl-Threonine | Simple median | -13.335 | 11.148 | (-35.184, 8.514) | 0.232 |
|  | Weighted median | -23.526 | 11.039 | (-45.163, -1.889) | 0.033 |
|  | Inverse variance weighted | -15.855 | 8.124 | (-31.779, 0.068) | 0.051 |
|  | MR Egger | -61.043 | 42.534 | -144.408, 22.322 | 0.151 |
| **Sphingosine_IQGAP1** | Simple median | 6.47E-05 | 3.02E-05 | (5.480e-06, 0.0001) | 0.032 |
|  | Inverse variance weighted | 5.26E-05 | 2.11E-05 | (1.126e-05, 9.396e-05) | 0.013 |
|  | PRESSO MR | 7.44E-05 | 1.93E-05 | (3.667e-05, 0.0001) | **0.0005** |
|  | MR Egger Intercept | 21.986 | 56.54 | (-88.831, 132.803) | 0.697 |
|  | PRESSO Global test |  |  |  | 1 |
| IQGAP1_ Sphingosine | Simple median | 166.485 | 104.589 | (-38.506, 371.476) | 0.111 |
|  | Weighted median | 197.619 | 102.769 | (-3.804, 399.041) | 0.054 |
|  | Inverse variance weighted | 179.081 | 72.029 | (37.907, 320.256) | 0.013 |
|  |  |  |  |  |  |
| **GLUCOSAMINE_LUZP6** | Simple median | 2.52E-05 | 1.25E-05 | (6.350e-07, 4.968e-05) | 0.044 |
|  | Weighted median | 5.23E-05 | 1.35E-05 | (2.573e-05, 7.882e-05) | **0.0001** |
|  | Inverse variance weighted | 3.04E-05 | 7.78E-06 | (1.516e-05, 4.568e-05) | **9.31E-05** |
|  | MR Egger | 6.35E-05 | 1.98E-05 | (2.476e-05, 0.0001) | **0.001** |
|  | PRESSO MR | 1.97E-05 | 6.41E-06 | (7.186e-06, 3.229e-05) | **0.003** |
|  | MR Egger Intercept | -42.206 | 23.183 | (-87.643, 3.232) | 0.069 |
|  | PRESSO Global test |  |  |  |  |
| LUZP6_ GLUCOSAMINE | Simple median | -61.84 | 106.019 | (-269.634, 145.953) | 0.56 |
|  | Weighted median | -24.964 | 102.962 | (-226.766, 176.838) | 0.808 |
|  | Inverse variance weighted | -1.023 | 70.953 | (-140.089, 138.042) | 0.988 |
|  |  |  |  |  |  |
| **Isobutyrylcarnitine_LUZP6** | Simple median | -2.21E-05 | 5.94E-06 | (-3.377e-05, -1.048e-05) | **0.0002** |
|  | Weighted median | -2.42E-05 | 6.31E-06 | (-3.654e-05, -1.181e-05) | **0.0001** |
|  | Inverse variance weighted | -1.40E-05 | 4.17E-06 | (-2.213e-05, -5.799e-06) | **0.0008** |
|  | MR Egger | -4.71E-05 | 1.84E-05 | (-8.305e-05, -1.113e-05) | **0.01** |
|  | PRESSO MR | -1.59E-05 | 4.34E-06 | (-2.441e-05, -7.408e-06) | **0.0004** |
|  | MR Egger Intercept | 73.845 | 39.852 | (-4.264, 151.954) | 0.064 |
|  | PRESSO Global test |  |  |  | 1 |
| LUZP6_Isobutyrylcarnitine | Simple median | -176.35 | 218.633 | (-604.862, 252.167) | 0.42 |
|  | Weighted median | -134.219 | 219.712 | (-564.845, 296.408) | 0.541 |
|  | Inverse variance weighted | -9.395 | 160.399 | (-323.770, 304.981) | 0.953 |
| **Plasmenyl-LysoPE_LUZP6** | Inverse variance weighted | -0.0006 | 0.0002 | (-0.001, -0.0001) | **0.009** |
|  | MR Egger | -0.004 | 0.0009 | (-0.006, -0.002) | **3.40E-05** |
|  | PRESSO MR | -0.0008 | 0.0002 | (-0.001, -0.0004) | **6.96E-05** |
|  | PRESSO Global test |  |  |  | 1 |
| LUZP6_Plasmenyl-LysoPE | Simple median | -9.362 | 5.15 | (-19.456, 0.732) | 0.069 |
|  | Weighted median | -2.09 | 5.024 | (-11.936, 7.756) | 0.677 |
|  | Inverse variance weighted | -6.518 | 3.398 | (-13.178, 0.142) | 0.055 |
|  |  |  |  |  |  |
| **3-(2-HYDROXYPHENYL)PROPANOATE_LUZP6** | Simple median | -2.59E-05 | 1.18E-05 | (-4.897e-05, -2.774e-06) | 0.028 |
|  | Inverse variance weighted | -1.53E-05 | 6.39E-06 | (-2.783e-05, -2.783e-06) | 0.017 |
|  | PRESSO MR | -3.14E-05 | 7.33E-06 | (-4.571e-05, -1.699e-05) | **4.07E-05** |
|  | MR Egger Intercept | -25.611 | 15.996 | (-56.962, 5.741) | 0.109 |
|  | PRESSO Global test |  |  |  | 1 |
| LUZP6_3-(2-HYDROXYPHENYL)PROPANOATE | Weighted median | -78.944 | 122.662 | (-319.3567, 161.468) | 0.52 |
|  | Inverse variance weighted | -112.16 | 77.958 | (-264.954, 40.635) | 0.15 |
|  | MR Egger | 161.811 | 273.373 | (-373.990, 697.611) | 0.554 |
|  |  |  |  |  |  |
| **UDCA_LUZP6** | Weighted median | -0.0002 | 8.31E-05 | (-0.0004, -8.561e-05) | **0.003** |
|  | PRESSO MR | -0.0001 | 5.13E-05 | (-0.0002, 4.5e-07) | **0.006** |
|  | MR Egger Intercept | 19.265 | 15.84 | (-11.780, 50.310) | 0.224 |
|  | PRESSO Global test |  |  |  | 1 |
| LUZP6_UDCA | Simple median | -1.315 | 13.116 | (-27.022, 24.391) | 0.92 |
|  | Weighted median | -8.159 | 13.2 | (-34.030, 17.712) | 0.537 |
|  | Inverse variance weighted | -1.828 | 8.847 | (-19.167, 15.512) | 0.836 |
| **GLUCOSAMINE _MPEG1** | Weighted median | -5.99E-05 | 2.25E-05 | (-0.0001, -1.578e-05) | **0.008** |
|  | Inverse variance weighted | -4.61E-05 | 1.35E-05 | (-7.258e-05, -1.959e-05) | **0.0007** |
|  | MR Egger | -6.59E-05 | 2.49E-05 | (-0.0001, -1.717e-05) | **0.008** |
|  | PRESSO MR | -5.81E-05 | 1.25E-05 | (-8.253e-05, -3.357e-05) | **9.39E-06** |
|  | MR Egger Intercept | 31.4837 | 33.138 | (-33.465, 96.432) | 0.342 |
| MPEG1_ GLUCOSAMINE | Simple median | -4.439 | 87.021 | (-174.998, 166.119) | 0.959 |
|  | Weighted median | 28.114 | 83.919 | (-136.365, 192.593) | 0.738 |
|  | Inverse variance weighted | 30.651 | 60.976 | (-88.859, 150.162) | 0.615 |
|  |  |  |  |  |  |
| **INDOLE-3-ACETATE _MPEG1** | Simple median | 0.0008 | 0.0003 | (0.0002, 0.001) | **0.013** |
|  | Weighted median | 0.0009 | 0.0003 | (0.0003, 0.001) | **0.004** |
|  | Inverse variance weighted | 0.0007 | 0.0002 | (0.0003, 0.001) | **0.0005** |
|  | MR Egger | 0.002 | 0.0008 | (0.0002, 0.003) | 0.028 |
|  | PRESSO MR | 0.0009 | 0.0002 | (0.0005, 0.001) | **2.54E-06** |
|  | MR Egger Intercept | -100.643 | 75.904 | (-249.412, 48.126) | 0.185 |
| MPEG1_INDOLE-3-ACETATE | Simple median | -3.41 | 8.581 | (-20.228, 13.408) | 0.691 |
|  | Weighted median | -3.269 | 8.289 | (-19.514, 12.977) | 0.693 |
|  | Inverse variance weighted | 0.011 | 6.033 | (-11.814, 11.835) | 0.999 |
| **N-ACETYLNEURAMINATE_MPEG1** | Weighted median | -0.0002 | 6.73E-05 | (-0.0003, -2.812e-05) | 0.017 |
|  | PRESSO MR | 0.0001 | 3.84E-05 | (2.481e-05, 0.0002) | **0.006** |
|  | MR Egger Intercept | 52.408 | 34.831 | (-15.860, 120.675) | 0.132 |
| MPEG1_N-ACETYLNEURAMINATE | Simple median | 27.418 | 23.044 | (-17.749, 72.584) | 0.234 |
|  | Weighted median | 33.592 | 22.463 | (-10.435, 77.618) | 0.135 |
|  | Inverse variance weighted | 42.647 | 16.284 | (10.731, 74.563) | **0.009** |
|  | MR Egger | 28.501 | 105.773 | (-178.809, 235.811) | 0.788 |
|  |  |  |  |  |  |
| **3-(2-HYDROXYPHENYL)PROPANOATE_MPEG1** | Simple median | 8.17E-05 | 2.51E-05 | (3.242e-05, 0.0001) | **0.001** |
|  | Inverse variance weighted | 5.00E-05 | 1.48E-05 | (2.095e-05, 7.905e-05) | **0.0007** |
|  | PRESSO MR | 0.0001 | 1.48E-05 | (7.105e-05, 0.0001) | **2.25E-11** |
|  | PRESSO Global test |  |  |  | 1 |
| MPEG1_3-(2-HYDROXYPHENYL)PROPANOATE | Simple median | 95.246 | 87.729 | -76.701, 267.192 | 0.278 |
|  | Weighted median | 97.054 | 85.817 | -71.145, 265.253 | 0.258 |
|  | Inverse variance weighted | 110.527 | 63.691 | -14.306, 235.360 | 0.083 |
| **INDOLE-3-ACETATE_PLCB2** | Simple median | -0.0002 | 0.0001 | (-0.0005, -2.174e-05) | 0.032 |
|  | Weighted median | -0.0002 | 0.0001 | (-0.0005, -2.64e-05) | 0.028 |
|  | PRESSO MR | -0.0002 | 6.74E-05 | (-0.0003, -6.795e-05) | **0.008** |
|  | MR Egger Intercept | 28.923 | 27.597 | (-25.166, 83.011) | 0.295 |
|  | PRESSO Global test |  |  |  | 1 |
| PLCB2_INDOLE-3-ACETATE | Simple median | 16.523 | 9.962 | -3.002, 36.047 | 0.097 |
|  | Weighted median | 16.446 | 9.817 | -2.794, 35.687 | 0.094 |
|  | Inverse variance weighted | 6.502 | 6.865 | -6.953, 19.957 | 0.344 |
| **3-(2-HYDROXYPHENYL)PROPANOATE_PLCB2** | Simple median | -2.16E-05 | 9.06E-06 | -3.934e-05, -3.808e-06 | 0.017 |
|  | MR Egger | 1.8 | 8.84E-06 | (6.803e-07, 3.533e-05) | 0.042 |
|  | PRESSO MR | -1.77E-05 | 5.47E-06 | (-2.846e-05, -7.023e-06) | **0.002** |
|  | PRESSO Global test |  |  |  | 1 |
| PLCB2_3-(2-HYDROXYPHENYL)PROPANOATE | Simple median | 113.923 | 105.154 | (-92.174, 320.021) | 0.279 |
|  | Weighted median | 42.027 | 104.083 | (-161.972, 246.026) | 0.686 |
|  | Inverse variance weighted | 51.701 | 72.538 | (-90.470, 193.872) | 0.476 |
| **GLUCOSAMINE_PTGS1** | Weighted median | 7.35E-05 | 2.50E-05 | (2.450e-05, 0.0001) | **0.003** |
|  | Inverse variance weighted | 5.18E-05 | 1.66E-05 | (1.932e-05, 8.419e-05) | **0.002** |
|  | PRESSO MR | 0.0002 | 4.17E-05 | (8.407e-05, 0.0002) | **7.00E-05** |
|  | PRESSO Global test |  |  |  | 1 |
| PTGS1_GLUCOSAMINE | Simple median | -29.163 | 35.368 | (-98.484, 40.158) | 0.41 |
|  | Weighted median | -25.446 | 35.297 | (-94.626, 43.734) | 0.471 |
|  | Inverse variance weighted | -2.984 | 24.04 | (-50.101, 44.134) | 0.901 |
|  |  |  |  |  |  |
| **INDOLE-3-ACETATE_PTGS1** | Weighted median | 0.0008 | 0.0003 | (0.0002, 0.001) | **0.01** |
|  | Inverse variance weighted | 0.0007 | 0.0002 | (0.0003, 0.001) | **0.001** |
|  | PRESSO MR | 0.0006 | 0.0002 | (0.0002, 0.001) | **0.003** |
|  | MR Egger Intercept | 56.099 | 86.968 | (-114.355, 226.554) | 0.519 |
|  | PRESSO Global test |  |  |  | 1 |
| PTGS1_INDOLE-3-ACETATE | Simple median | 5.132 | 3.505 | (-1.738, 12.001) | 0.143 |
|  | Weighted median | 4.322 | 3.502 | (-2.542, 11.185) | 0.217 |
|  | Inverse variance weighted | 7.478 | 2.395 | (2.785, 12.171) | **0.002** |
|  | MR Egger | 16.16 | 12.376 | (-8.096, 40.416) | 0.192 |
|  |  |  |  |  |  |
| **3-(2-HYDROXYPHENYL)PROPANOATE_PTGS1** | Simple median | -7.87E-05 | 2.44E-05 | (-0.0001, -3.083e-05) | **0.001** |
|  | Inverse variance weighted | -4.62E-05 | 1.31E-05 | (-7.188e-05, -2.055e-05) | **0.0004** |
|  | PRESSO MR | -7.65E-05 | 1.48E-05 | (-0.0001, -4.74e-05) | **1.14E-06** |
|  | MR Egger Intercept | -35.629 | 33.26 | (-100.817, 29.559) | 0.284 |
|  | PRESSO Global test |  |  |  | 1 |
| PTGS1_3-(2-HYDROXYPHENYL)PROPANOATE | Simple median | -55.593 | 35.434 | (-125.042, 13.857) | 0.117 |
|  | Weighted median | -58.287 | 35.936 | (-128.721, 12.147) | 0.105 |
|  | Inverse variance weighted | -54.748 | 24.554 | (-102.872, -6.623) | 0.026 |
|  | MR Egger | -76981.59 | 136580.254 | (-344673.968, 190710.788) | 0.573 |
| **GLUCOSAMINE_UGGT1** | Simple median | -0.0001 | 6.25E-05 | (-0.0003, -6.897e-06) | 0.038 |
|  | Weighted median | 0.0002 | 5.73E-05 | (9.381e-05, 0.0003) | **0.0003** |
|  | Inverse variance weighted | 7.41E-05 | 3.63E-05 | (2.955e-06, 0.0001) | 0.041 |
|  | MR Egger | 0.0004 | 7.88E-05 | (0.0002, 0.0005) | **7.22E-06** |
|  | PRESSO Global test |  |  |  | 1 |
| UGGT1_GLUCOSAMINE | Simple median | 10.447 | 8.499 | -6.210, 27.105 | 0.219 |
|  | Weighted median | -11.95 | 9.333 | -30.242, 6.342 | 0.2 |
|  | Inverse variance weighted | 6.982 | 4.372 | -1.588, 15.551 | 0.11 |
| **Plasmenyl-LysoPE_UGGT1** | Simple median | -0.004 | 0.001 | (-0.007, -0.0009) | 0.011 |
|  | Inverse variance weighted | -0.002 | 0.001 | (-0.004, -0.0003) | 0.02 |
|  | PRESSO MR | -0.002 | 0.0007 | (-0.003, -0.0006) | **0.0005** |
|  | MR Egger Intercept | -50.321 | 93.451 | (-233.481, 132.839) | 0.59 |
|  | PRESSO Global test |  |  |  | 1 |
| UGGT1_Plasmenyl-LysoPE | Simple median | -1.31 | 0.404 | (-2.102, -0.517) | 0.001 |
|  | Weighted median | 0.709 | 0.401 | (-0.078, 1.495) | 0.077 |
|  | Inverse variance weighted | -0.37 | 0.219 | (-0.798, 0.059) | 0.091 |
|  | MR Egger | 0.525 | 0.414 | (-0.286, 1.337) | 0.205 |

*Bold demonstrates significantly causal association (P < 0.01).
